# Mechanism-anchored profiling derived from epigenetic networks predicts outcome in acute lymphoblastic leukemia

**DOI:** 10.1186/1471-2105-10-S9-S6

**Published:** 2009-09-17

**Authors:** Xinan Yang, Yong Huang, James L Chen, Jianming Xie, Xiao Sun, Yves A Lussier

**Affiliations:** 1 Center for Biomedical Informatics and Section of Genetic Medicine, Department of Medicine, The University of Chicago, Chicago, IL 60637, USA; 2 State Key Laboratory of Bioelectronics, Southeast University, 210096 Nanjing, PR China; 3 The University of Chicago Cancer Research Center, and The Ludwig Center for Metastasis Research, The University of Chicago, Chicago, IL 60637, USA; 4 The Institute for Genomics and Systems Biology, and the Computational Institute, The University of Chicago, Chicago, IL 60637, USA

## Abstract

**Background:**

Current outcome predictors based on "molecular profiling" rely on gene lists selected without consideration for their molecular mechanisms. This study was designed to demonstrate that we could learn about genes related to a specific mechanism and further use this knowledge to predict outcome in patients – a paradigm shift towards accurate "mechanism-anchored profiling". We propose a novel algorithm, PGnet, which predicts a tripartite mechanism-anchored network associated to epigenetic regulation consisting of phenotypes, genes and mechanisms. Genes termed as GEMs in this network meet all of the following criteria: (i) they are co-expressed with genes known to be involved in the biological mechanism of interest, (ii) they are also differentially expressed between distinct phenotypes relevant to the study, and (iii) as a biomodule, genes correlate with both the mechanism and the phenotype.

**Results:**

This proof-of-concept study, which focuses on epigenetic mechanisms, was conducted in a well-studied set of 132 acute lymphoblastic leukemia (ALL) microarrays annotated with nine distinct phenotypes and three measures of response to therapy. We used established parametric and non parametric statistics to derive the PGnet tripartite network that consisted of 10 phenotypes and 33 significant clusters of GEMs comprising 535 distinct genes. The significance of PGnet was estimated from empirical p-values, and a robust subnetwork derived from ALL outcome data was produced by repeated random sampling. The evaluation of derived robust network to predict outcome (relapse of ALL) was significant (p = 3%), using one hundred three-fold cross-validations and the shrunken centroids classifier.

**Conclusion:**

To our knowledge, this is the first method predicting co-expression networks of genes associated with epigenetic mechanisms and to demonstrate its inherent capability to predict therapeutic outcome. This PGnet approach can be applied to any regulatory mechanisms including transcriptional or microRNA regulation in order to derive predictive molecular profiles that are mechanistically anchored. The implementation of PGnet in R is freely available at .

## Background

By design, predictors of outcome based on gene expression profiles are based on gene lists that do not require knowledge of biological processes or molecular mechanisms [1]. Though expression arrays have been widely studied to improve prediction of clinical outcome and to aid the decision of treatment strategy for cancer, the resulting long list of genes lacking mechanistic background is thus difficult to interpret to infer their biological or clinical implications. Additionally, a poor outcome may be caused by a diversity of molecular disorders, for which the individual contribution may vary in different patients suffering from the same cancer [2]. In some cases, profiles are accompanied with follow-on enrichment studies or curated annotations that predict their possible mechanisms; while in other cases, functional clustering has been proposed to understand microarray data profiles [3]. In this manuscript, we propose a novel computational strategy based on genes associated to known biological mechanisms to derive mechanism-anchored expression profiles *ab initio *that can accurately predict disease outcome.

We hypothesized that those co-expression modules, which are predictive of outcome, can be computationally derived from genes known to regulate or to be regulated by epigenetic mechanisms in previous studies and from novel microarray expression specifically designed for a new phenotype for which the epigenetic mechanisms may not be well understood [4,5]. Nearly every cancer consists of genetic mutations of the transformed cells as well as epigenetic abnormalities of non-mutational changes to DNA that lead to alterations in gene expression [6]. While genetic abnormalities found in cancer typically affect cancer-promoting oncogenes and tumor suppressor genes, the epigenetic regulation of molecular functions involves reversible interactions which can affect gene expression such as (i) DNA methylation [7], (ii) histone modification [8], (iii) RNA transcription and the resulting proteins [9,10] or miRNAs [11], that influence chromatin structure. For example, histone deacetylation and the methylation of the promoter region can affect binding of transcriptional factors to these DNA regions and result in transcriptional silencing partly due to chromatin remodeling [12]. Indeed, combination therapy with inhibitors of DNA methyltransferase and histone deacetylase is under investigation in cancer [13-15]. Additionally, epigenetic events occur in the coordinated behavior of epigenetic proteins that regulate gene expressions [15]. To demonstrate the applicability of the proposed phenotype-genotype-network" method (PGnet), a set of known biological mechanism-anchored genes are required as the "seed" (input). Although this method can be generalized to other molecular mechanisms and other diseases, we focused this study on epigenetic alterations in acute lymphoblastic leukemia (ALL).

To compute co-expression modules of genes in disease and to infer their interplay generally require the integration of data from a wide variety of sources [16,17]. For example, some computational methods have been developed to indentify shared regulatory inputs, functional pathways and genetic interactions [2,18-21]. We have also previously shown that co-expression patterns of genes found in expression arrays designed around specific phenotypes can be recapitulated in gene-phenotype relationships derived from database/literature mining [22]. Further, genome-scale reverse engineering of regulatory mechanisms in expression arrays have been developed and successfully applied in mammalian cells [23]. For example, A method called *ARACNE *has been shown to be effective in practice by using a mutual information theoretic approach which focuses on direct co-expression of genes [24,25]. However, unsupervised combinations of every molecular element that may interact via one or more intermediaries can lead to a problem of multiplicity due to the escalating number of comparisons and thus to a loss of statistical power. Another method, *FunNet*, addresses multiplicity by combining gene expression data with Gene Ontology [26,27] or KEGG [28] annotations and further performs transcriptional functional analysis over co-expression [29]. Another method, *StAM*, identifies expression signature by focusing on biological processes which can characterize subgroup of patients [2]. However, these methods are not designed to compute regulatory networks that would also be differentially expressed in multiple phenotypic contexts as well as co-expressed in each individual. Our approach differs from these previous methods in that genes are integrated to the profile signature if: **i) **they are associated *ab initio *to the biological mechanism of interest (here epigenetics), and **ii) **they are derived from the a non-parametric statistic taking into account comprehensive expression patterns of the every gene in the microarray rather than from a subset of the differential-expressed ones.

We hypothesize that using supervised pair-wise measurements from microarray data together with robust feature selection technology [1], we are more likely to construct meaningful, epigenetic mechanism-anchored, co-expression networks that are predictive of leukemia outcome. To this end, we propose a novel supervised non-parametric algorithm (PGnet) that builds a tripartite network derived from (i) microarray expression profiles, and (ii) prior knowledge about biological mechanisms. PGnet is designed to identify sets of mechanism-anchored genes that are both consistently co-expressed across arrays and differentially expressed between phenotypic conditions.

## Methods

### Arrays and phenotypes

We selected a large array dataset published by the Downing research group (ALL arrays) [30] that comprises well characterized subtypes of ALL and other clinical phenotypes, including cytogenetic characteristics, molecular status and patient outcomes. The details for leukemia phenotypes and sample size are provided in Suppl. Methods (Additional file 1).

### Epigenetic Seed Genes – ESGs (Suppl. Methods (Additional file 1) and Suppl. Table 1 (Additional file 2))

Gene Ontology terms and PubMed were used to identify genes with epigenetic effects. Genes were subsequently mapped to Affymetrix probe-sets and curated into eight categories.

### Array analyses

The 132 ALL arrays were normalized with the variance stabilization and calibration normalization (vsn) method [31] using Bioconductor [32,33] package *compdiagTools *[34]. We then applied an additional inter quartile range (IQR) filter [35] to eliminate genes lacking sufficient variation across samples in expression (Suppl. Methods (Additional file 1)).

### Building a phenotype – gene network (PGnet)

**Figure 1:**
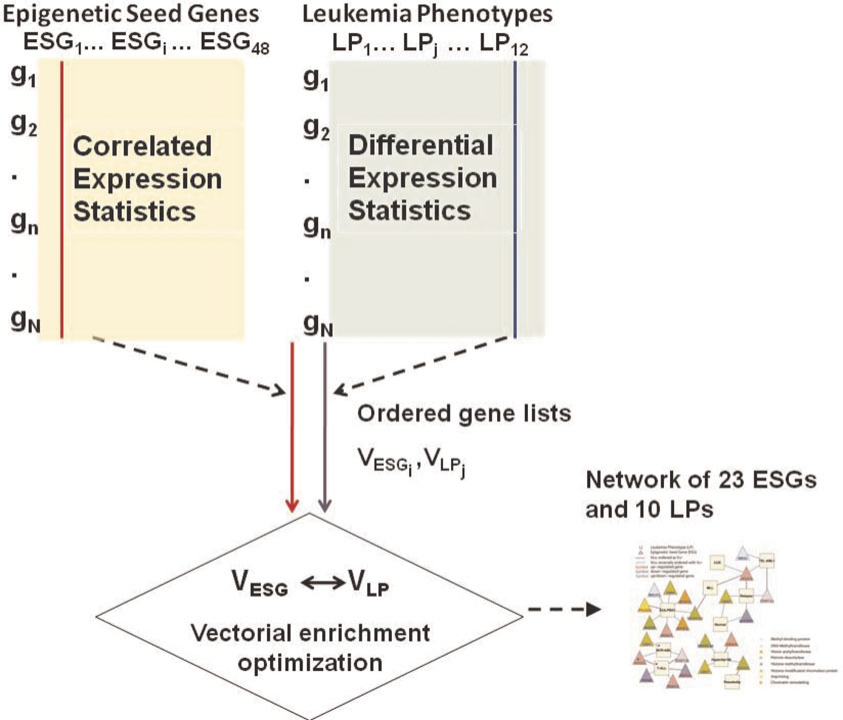
**Design of PGnet methods**. Two sets of data are required to construct the network: **(i) ***V*_*ESG *_*lists *are produced by analyzing the known epigenetic "seed genes" using the pairwise standard Pearson correlation coefficient of the vsn normalized gene expression levels between ESG and all genes *g*_*n *_that meet the IQR filter criteria across all samples where genes *g*_*n *_(n = 1,..., N), **(ii) ***V*_*LP *_*lists *are derived by analyzing the gene's differential expression in each phenotype of interest and evaluating its significance with an adjusted t-test. The rows in both *V*_*ESG *_and *V*_*LP *_*lists *represent every genes in the microarray that meet the IQR filters, whereas the columns are either epigenetic seed genes in the *V*_*ESG *_*lists *or phenotypes in the *V*_*LP *_*lists*. We then use the PGnet methodology to develop a similarity vector between the epigenetic seed gene and a phenotype. We calculated the vectorial similarity between each pair of ordered expression of gene lists using a previously algorithm that we published, *orderedlist *(Suppl. Methods (Additional file 1)) [37]. The result is a ranked list of genes that are significantly associated based on their respective *V*_*ESGi *_and *V*_*LPj*_. We build a gene-phenotype network where relationships are similarity scores (**Fig. 2**). Legend: *g: *gene with microarray expression; *ESG: *epigenetic seed gene; *LP: *leukemia phenotype.

The construction of a network comprising "Leukemia Phenotypes" (LPs), "epigenetic seed Genes" (ESGs) and co-expressed genes required six steps (see Figure 1 and Suppl. Methods (Additional file 1) for details on steps and equations).

#### Step 1a: Vector of genes co-expressed with the mechanism seed genes

All genes that co-expressed with ESGs were sorted in a vector based on the Pearson correlation coefficient (PCC). Results were denoted as .

#### Step 1b: Vector of differentially expressed genes in ALL

At the same time, all genes were also sorted based on their adjusted Student t-test conducted between the phenotype of interest against the remaining pooled phenotypes. Results were denoted as . Bioconductor [32,33] package *stats *was used to calculate the PCC and the package *Twilight *[36] was used to calculate the adjusted t-score parameter.

#### Step 2

To compare two ordered lists of gene expressions, we used *OrderedList *[37-39] from Bioconductor [32,33], a non-parametric quantitative vectorial enrichment method that we previously published, and has been shown more sensitive to detect significant departure from a predicted distribution than semi-quantitative enrichment approaches such as the Fisher's Exact Test or the the Chi-square test. We calculated a matrix of similarity scores *M*_*s *_= (*s*_*i*, *j*_), where each score *s*_*i*, *j *_assessed the pair-wise similarities between two vectors. The fist vector is the ordered co-expression coefficients *V*_*ESGi *_and the second one is the ordered differential expression statistics *V*_*LP*j_. The similarity score gives higher weights to ranking extremes: the top and bottom ranks in both lists. In our method, we compared the ranking of genes in the co-expression set with those gene ranks from the phenotypic set. This resulted in a total of two comparisons for each phenotype/"seed gene" combination (correlation and anti-correlation).

#### Step 3: Vectorial Enrichment Optimization (VEO)

**Figure 2:**
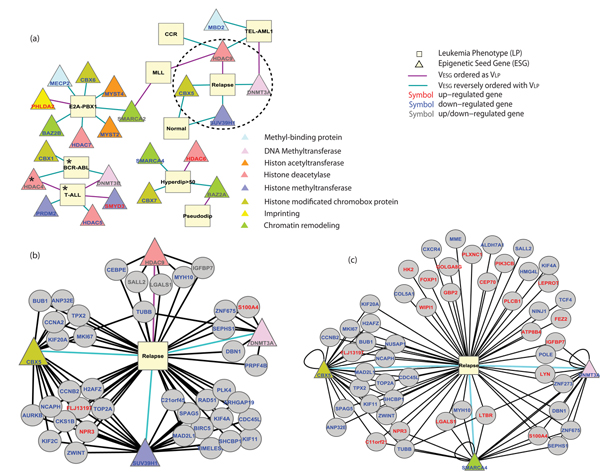
**Network modelling of epigenetic genes – phenotypes in ALL derived from PGnet**. The complete tripartite network produced by PGNet is available as Suppl. Fig. 1 (Additional file 14) and Suppl. Table 2 (Additional file 3). Here, panel (a) shows a bipartite subset of 33 statistically significant associations linking 23 distinct epigenetic seed genes (ESGs, yellow triangle) to 10 distinct leukemia phenotypes (yellow squares). Distinct background colors of ESG gene names indicate distinct molecular function or biological process related to or targeted by epigenetic regulation (Suppl. Table 1 (Additional file 2)). Two LPs and one ESG with asterisk were selected to show the details of a biomodule derived by PGnet (Figure 4). Red genes are those for which the expression is up-regulated in the associated ALL phenotype, while blue ones are with down-regulated expression, and grey ones are related to more than one phenotype with alternate up-down regulations (details of the full network in Suppl. Table 2 (Additional file 3)). Panel (b) shows a tripartite network that includes GEMs (grey circles) and focuses on the circled subset of Panel a: the "ALL relapse". Panel (c) is a robust sub-network associated to ALL outcome (relapse vs. continuous complete remission (CCR)). Three ESGs and 53 GEMs were obtained by 100 repetitions of 3-fold cross-validation of PGnet operating on the subset of ALL arrays comprising 87 patients experiencing either "CCR" or "relapse" (n > 32, details in Suppl. Methods (Additional file 1)). Note that ALL subtypes associated PGnet in panel a) and b) were derived from all 132 patients (in this case the biomodules of the sub-network 2b pertain to patients with relapse and not everything else). The robust sub-network in panel c) was conducted for training a predictor of outcome. Only 87 out of the 132 samples contained outcome information related to ALL relapse ("relapse" or "CCR" of ALL). Please note that some patients did have an outcome of secondary AML, a distinct form of leukemia, and were excluded from panel 2c because this disease occurs at a later stage and the authors of the dataset did not disclose the outcome of ALL for these patients. Thus the biomodules of Figure 2c overlap partially with those of Figure 2b.

To evaluate the statistical significance of the similarity score, we generated 2,000 controls through the permutation of each list of gene ranks and calculated empirical p-values based on random scores. Two networks were generated. In this proof-of-concept study, the arbitrary but uniform significance threshold (T = 200, Suppl. Methods (Additional file 1)) of included ranks was chosen to define a set of GEMs with higher differential expression in VEO that would yield a small network (Suppl. Table 2 (Additional file 3) and Figure 2), where the co-expressed with known "epigenetic seed genes" and phenotype-specific genes are the genes within the top 200 or bottom 200 in either of the two lists. An optimal length for these ordered gene lists can also be determined by unbiased optimization methods and can generate a larger network (Suppl. Table 3 (Additional file 4)), however for the purpose of simplicity of presentation – we kept the list at T = 200 for the main figures, which is within the range of length considered biologically significant in our unbiased and more comprehensive studies (Suppl. Table 3 (Additional file 4)). In both the arbitrary threshold (T = 200) and the optimal threshold cases, the significance of PGnet were estimated by an empirical p-value of similarity scores by permutation the ranks (number of permutations = 1000, Suppl. Methods). And the adjustment threshold of significance for vectorial similarity was conducted by controlling the false discovery rates (q-value = 0.02) [40,41] (Suppl. Methods (Additional file 1)).

#### Step 4

For each significant seed gene/phenotype pair we considered these to be "linked." By aggregating these seed gene/phenotype pairs, we developed a tripartite network PGnet.

#### Step 5: Visualization of the Tripartite Network

Meaning of shapes and colors in the network: triangle (epigenetic seed genes), circle (predicted GEMs) and box (phenotypes); red (up-regulated), blue (down-regulated) and grey for vertex of a gene had more than one linkage and was up-regulated in one condition but down-regulated in a different condition (Suppl. Methods (Additional file 1)). By color-coding the edges of the graph, we are providing a direction to each similarity vector, magenta line for correlation whereas turquoise for anti-correlation. With these vectors, one can judge how these genes express in a specific condition.

#### Step 6: Biological meaning of the network

Using Gene ontology, we conducted an enrichment of the molecular function and biological processes among the genes identified in the PGnet biomodules in order to characterize biologically the network and we also reviewed the literature for the genes involved in the biomodules associated to BCR-ABL, T-ALL and hyperdiploidy. Thus the resulting set of genes termed as Genes significantly Expressed with the Mechanism (GEMs) in the epigenetic context of this network meet all of the following criteria: (i) they are co-expressed with genes known to be involved in the biological mechanism of interest, (ii) they are also differentially expressed between distinct phenotypes relevant to the study, and (iii) as a biomodule, genes correlate with both the mechanism and the phenotype.

### Robust predictive network and evaluation

The predictive capabilities of the derived network were evaluated with two approaches: (a) quantitative computational studies of the accuracy of the predictor of outcome, and (b) qualitative comparison of the PGnet method to that of another reverse engineering one.

**Figure 3:**
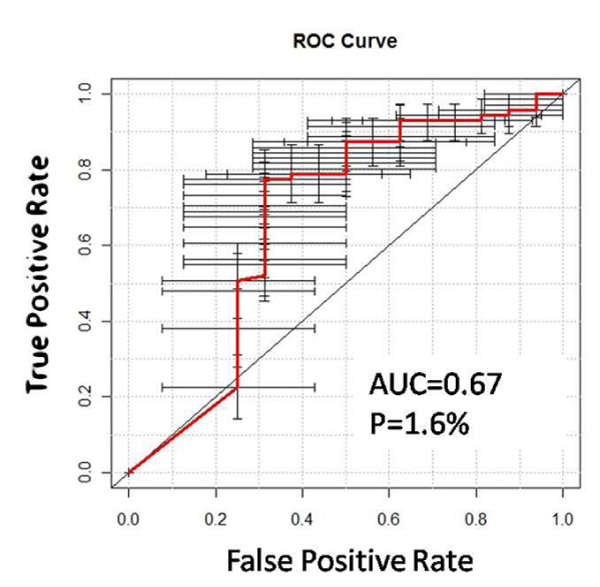
**Evaluation of the outcome predictor of "ALL Relapse" derived from the mechanism-anchored expression profile obtained by PGnet**. The 87 leukemia patients with "CCR" or "relapse" information were randomly divided into three folds, two of which were used to identify the predictor of outcome ("CCR" vs. "relapse", Figure 2c). The predictor consisted of GEMs and ESGs associated to "relapse" to train a linear SVM model, and the remaining one was used as a blinded test set. Three-fold cross-validations were repeated 100 times. The resulting Receiver Operating Characteristic (ROC) curve, the area under the curve (AUC) and corresponding p-values were calculated by Bioconductor package *verification*. Horizontal and vertical "error bars" represent the 95% confidence intervals of the predictor. Regions where the error bars are above the diagonal line represent a better prediction than chance. Overall, the AUC was significantly different than that of a random predictor of "ALL relapse" (P = 1.6%).

(a) To demonstrate the accuracy of the derived network to predict relapse, we performed a conservative evaluation consisting of one hundred three-fold cross-validation (CV) studies of the PGnet method. In other words, as shown in Figure 2c, the network was derived from 2/3 of the randomly selected patients and the evaluation was conducted on the remaining third. The random selection was conducted to conserve the respective group sizes (normal, cancer) and was considered a more adequate and severe control [42]. This procedure was repeated one hundred times on different random resamplings. Two different predictive methods were used as well: (i) Prediction Analysis for Microarrays Class Prediction [43] (PAM) that does not involve any machine learning and (ii) the Support Vector Machine [44] (SVM) (Suppl. Methods (Additional file 1) and Suppl. Fig. 2 (Additional file 5)). The resulting receiver operating characteristic (ROC) curve, area under the curve (AUC) and corresponding p-values were calculated by the Bioconductor [32,33] package *verification *[45]. A robust molecular signature is one that repeatedly appears by random sampling [1]. We further identified the GEMs correlated with the ESGs that were identified as robust [1]. The robust ESGs refer to those GEMs that were among the top 5% frequencies in the one hundred iterations of the 3-fold cross-validation (Figure B in Suppl. Methods (Additional file 1)). Figure 3 illustrates the sub-network associated to the comparison between "Relapse" and the "continuous complete remission – CCR" phenotypes.

(b) Finally we compared our results to those obtained by a straightforward reverse engineering method (ARACNE).

## Results

Seventy-one distinct epigenetic seed genes were identified in the literature review and denoted as "seed gene" candidates for input in PGnet (Suppl. Table 1 (Additional file 2)). In the 132 ALL arrays, 7,256 out of 12,997 unique genes and 48 out of the 71 subset of ESGs satisfy the IQR filter (bolded genes in Suppl. Table 1 (Additional file 2)). The 48 ESGs are thus enriched *ab initio *among genes differentially expressed in ALL samples suggesting a biological relevance in ALL (p = 5%, Fisher's Exact Test).

We built a gene-phenotype network specific for epigenetic genes clusters in ALL as shown in Figures 2a and 2b. The derived network comprises 33 significant nodes, including eight clinical subtypes of ALL and two outcome conditions (LP, vertex in yellow box), 23 epigenetic seed genes (ESG, yellow triangle) and 535 genes that co-express with ESGs (GEM, grey circle). Three of these ESGs and 299 GEMs are up-regulated in association to their phenotype(s) as compared with their expression associated to the remaining pooled phenotypes, while 14 ESGs and 203 GEMs are down-regulated. In addition, 6 ESGs and 33 GEMs were up-regulated in one phenotype but down-regulated in a different phenotype, which are phenotype specifically differential expressions (see Step 3 of **Methods**). A summary of predictions is provided in Suppl. Table 2 (Additional file 3). These 23 ESGs genes are highly co-expressed with epigenetic genes and also highly differentially expressed in distinct ALL phenotypes groups (*CBX1, CBX5, CBX6, CBX7, PHLDA2, BAZ2A, BAZ2B, MYST2, MYST4, MECP2, SMARCA2, HDAC4, HDAC5, HDAC6, HDAC7A, HDAC9, SMARCA4, SMYD3, SUV39H1, DNMT3A, DNMT3B, PRDM2 *and *MBD2*).

To validate the prognostic ability of the genes in PGnet associated with leukemia relapse, we performed one hundred three-fold cross-validations in two ways (Suppl. Methods (Additional file 1) and Suppl. Fig. 2 (Additional file 5)). Using the PAM classification that does not require machine-learning, the predictions were accurate (AUC = 0.65, p = 3%, Suppl. Fig. 3 (Additional file 6)). We also conducted a severe control by randomly selecting genes differentially expressed in the array and the p-values of the derived predictors ranged from 12% to 67%, further corroborating that the epigenetic network derived by PGnet is associated to the relapse outcome. Using SVM machine-learning to improve the predictions in a 3-fold cross over design, PGnet achieved a AUC = 0.67 (p = 1.6%) (Figure 3 in this manuscript). Precision and recall of the predictor in cross-validation studies are also significant (Suppl. Fig. 4 (Additional file 7)). The evaluation confirmed the detection of co-expression biomodules associated to epigenetic alterations can be utilized in the identification of ALL with poor prognosis [46]. Supplementary Table 5 (Additional file 8) reports 52 robust GEMs together with 3 robust ESGs *(CBX5, SMARCA4 *and *DNMT3A*) associated with leukemia relapse (Suppl. Methods (Additional file 1)).

We further proceeded to identify biological enrichment in the distinct sets of genes associated with response to therapy and long-term maintenance of disease remission. There were 4 ESGs and a total of 39 GEMs associated with the phenotype "Relapse", such as *CCNA2, BUB1, MAD2L1, CDC45L *and *CCNB2*, etc, which were significantly enriched in one GO term: ATP binding (hypergeometric p = 3.5 × 10^-6^, Suppl. Table 4 (Additional file 9)). Interesting, ATP has been reported as treatment target of murine leukemic cells in vitro to reduce the number of leukemic clonogenic cells [47].

**Figure 4:**
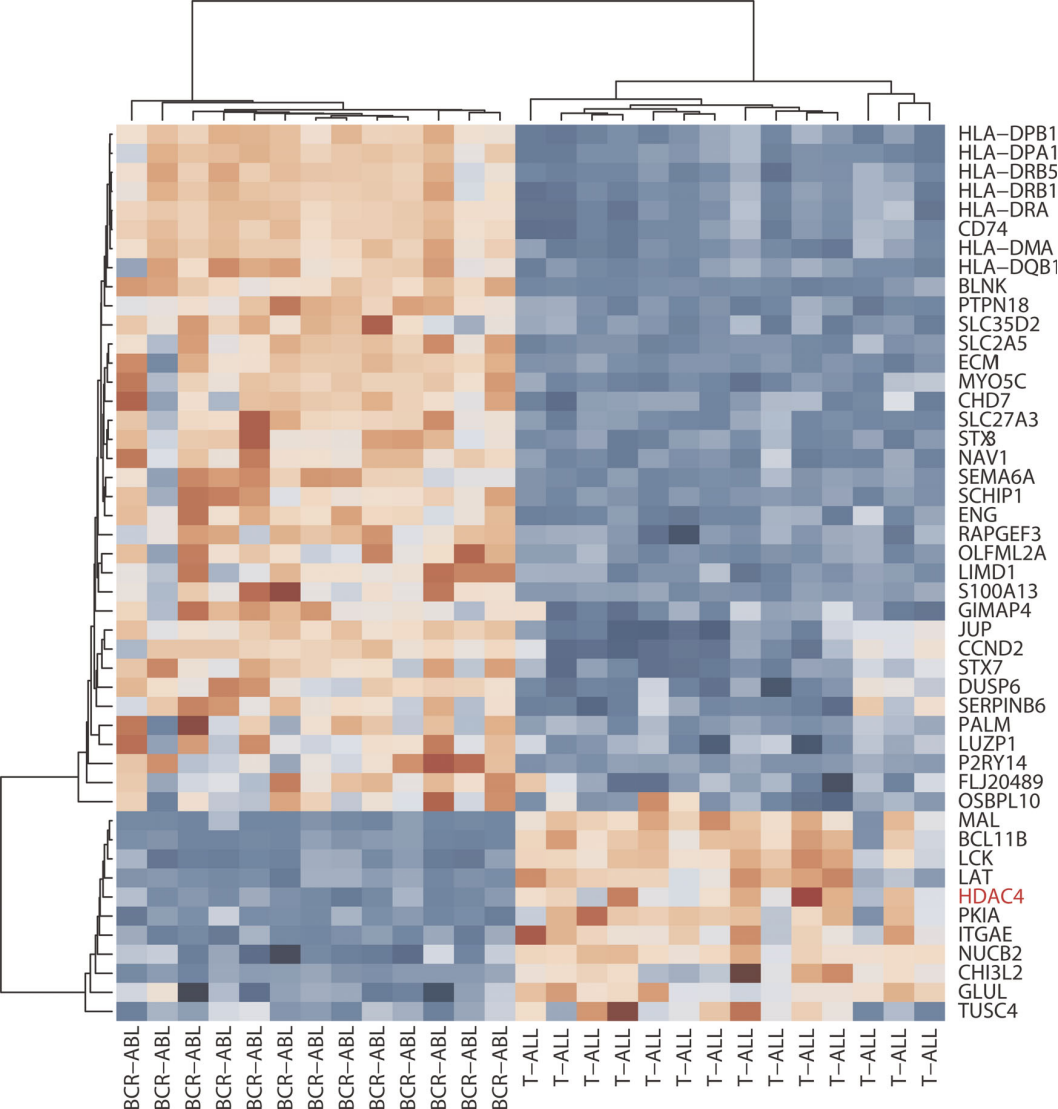
**Details on the expression biomodule associated with BCR-ABL and T-ALL phenotypes and the epigenetic mechanism of HDAC4**. The ESG (gene symbol in red) is positively correlated with part of its GEMs (n = 10) as down-regulated in "BCR-ABL" and up-regulated in "T-ALL", and negatively correlated with another parts of its GEMs (n = 36). 47 genes were derived from PGnet under optimal threshold (Suppl. Table 3 (Additional file 4)). The standard full agglomerative hierarchical clustering was performed on Spearman's rank correlation distance of normalized expression levels. The resulting heatmap was drawn using Bioconductor package *made4*.

Of note, PGnet is designed to discover mechanism-anchored biomodules that are phenotype-specific and that are consistently co-expressed across every patient. Figure 4 shows genes that are down-regulated in one phenotype and up-regulated in another phenotype and vice-versa (Suppl. Table 3 (Additional file 4)). In PGnet, 67 genes are involved in the expressional biomodules that are co-expressed with *HDAC4 *and *DNMT3B*, and yet are differentially expressed between "T-ALL" and "BCR-ABL" phenotypes. Enrichment analysis of these genes revealed only one significant enrichment: "MHC class II receptor activity" (GO:0032395, hypergeometric p-value = 1.6 × 10^-11^, Suppl. Table 4 (Additional file 9)). Further identification of the intersected regulation function of these epigenetic bio-modules requires experiments in vitro.

The GEMs derived from PGnet can distinguish their associated phenotype in addition to be co-expressed with their associated seed gene on transcript level. As an example in this study, Supplementary Figure 5 (Additional file 10) shows that the expression of 61 GEMs can clearly distinguish samples of "Hyperdiploid>50" from other ALL samples. There were 4 ESGs associated with the hyperdiploid karyotypes by PGnet. *HDAC6 *is a class IIB histon deacetylase and identified as target of anti-leukemia therapy [48]. Inhibition of *HDAC6 *disrupts the association of *HSP90 *with its chaperon proteins, resulting in ubiquitylation of certain oncogenes, such as *Bcr-Ab*l [49]. Three other genes were also down-regulated (*SMARCA4, BAZ2A *and *SMARCC2*): *SMARCA4 *is a drug target candidates in hyperdiploid multiple myeloma [50]; *BAZ2A *is a novel nucleolar chromatin remodeling machine [51], and *SMARCC2 *was among the top discriminating genes in the good prognosis subgroup of MLL [52]. Moreover, GEMs identified by PGnet significantly enriched among the top-100 marker genes in previous genome-scale studies of ALL (Fisher's test p < 2 × 10^-16^, Suppl. Table 6 (Additional file 11)).

## Discussion

### Comparison of the derived network with other computational methods

ARACNE [24,53] software was used to reverse engineer the transcriptional network in two ways. First, by providing the genes expression data for entire ALL samples as input, we compared our GEMs with genes identified by ARACNE (Result is given in Suppl. Table 7 (Additional file 12)). We provided ARACNE our 48 epigenetic seed genes and the expression data for samples in each phenotype as input, irrespectively. Subsequently, we got 12 different phenotype specific gene-gene networks. Each ARACNE network detects thousands of genes that with significant (p < 0.05) mutual information (MI) with inputted ESGs. By design, a majority of the genes detected by ARACNE are not phenotype specific. GEMs predicted by PGnet overlap with ARACNE's prediction for 31 of 33 ESGs (Suppl. Table 8 (Additional file 13)). However, PGnet differs from ARACNE in that it provides phenotypic information left out in ARACNE.

ARACNE, FunNet and PGnet provide co-regulation networks as an output and are thus "related"; however, they differ in several important ways: (i) PGnet and FunNet combine supervised technology and non-parametric methodology while ARACNE uses information theory; (ii) inputs to PGnet are expression levels and phenotypic associations of interest such as seed genes whereas FunNet requires full expression together with a reference list of all transcripts to be analyzed and ARACNE uses expression data exclusively; (iii) FunNet abstracts transcriptional functions from co-expression layer; and consequently (iv) PGnet's output is a tripartite network consisting of co-regulated genes and clinical/genetic characteristics of interest while ARACNE's or FunNet's outputs are uni-partite graphs. (v) The significant threshold of PGnet relates to the complete ordering of all genes to be analyzed whereas the significant threshold of FunNet is related to the co-expression of single gene. (vi) PGnet not only provides a degree of association between phenotypes but also sheds light on whether there was concordance in the directionality of the changes in expression level.

PGnet parallelizes two input vectors and finds sets of GEMs via vectorial enrichment optimization. Using measurements of differential expression and co-expression together, PGnet is more reliable in discovering phenotype-specific biomodules that are consistent across every patient than a simplified method that analyzes the expressed pattern of the epigenetic seed genes (ESGs) alone. First, a simplified method identifies none of GEMs from PGnet. However, we have shown some evidence indicating that the GEMs are more likely to be involved in specific epigenetic events than those directly calculated to be correlated to a phenotype of interest. Second, simpler alternate methods relying on co-expression or differential expression separately would identify only a the subset of ESGs from PGnet (data not shown), because these methods use an arbitrary threshold for significance of each gene and neglect the joint analysis of co-expression patterns with those of differential expression. In contrast, the "ESG-phenotype" linkage, which we proposed in PGnet, would be significant even if the epigenetic seed gene itself is not "significantly" differentially expressed in the linked phenotype (for instance, the seed genes that are not self-linked in Suppl. Fig. 1 (Additional file 14)).

Biologically, PGnet is an attractive technique as we know that mechanism-related genes have similar patterns of expression [4,20], and pathological mechanisms are easier to understand than genes by clinicians. Additionally, the non-parametric rank-correlation algorithm that we previously developed for Bioconductor can use the full range of the expression data for discovery instead of arbitrary statistical cut-offs [37-39]. We have extended it to derive phenotype-genotype correlations based on prior knowledge in addition to gene expression. Moreover, this tri-partite network allows to view genes for which the expression is specific to a phenotype of interest and also anchored to a biological mechanism.

### Future studies and limitations

Epigenetic gene regulation is one among many possible mechanisms involved in disease-specific gene aberrant activation. Better predictors of outcome can be developed using a more comprehensive number of biological mechanisms. The PGnet method could be expanded to a broader variety of biological mechanisms in order to provide more accurate mechanism-anchored profiles that predict therapeutic outcome (e.g. transcriptional and microRNA networks [54], Gene Ontology terms, KEGG, etc), however additional methods are required to control for multiplicity of mechanism while preserving accuracy of the derived tripartite networks. In addition, this PGnet is a supervised method that relies on prior knowledge about seed genes or gene products that regulate epidemic processes. Therefore, PGnet may "skew" the network accordingly, which may reflect only subset of the real regulatory relationship. Further improvement for finding disease associated and seed-gene regulated genes will likely require a refined assessment of co-expression, e.g. mutual information [24,55], instead of linear Pearson coefficient [37]. By design, PGnet identifies biomodules that are consistently co-expressed with the mechanism seed genes across all patient samples. However, there could exist mechanisms that are only co-expressed in some specific phenotypes and otherwise the co-expression patterns are lost. These particular biomodules may also contribute to mechanism-anchored predictors and require further methodological developments for their ascertainment. Future evaluations comparing the PGnet-derived predictors in other datasets are required, and we intend to proceed with multi-mechanism profiling that would in theory achieve higher precision and recall.

## Conclusion

We introduced and evaluated a novel algorithm, PGnet, to identify mechanism-anchored co-expression networks and to predict therapeutic outcome. PGnet differs from previous reverse engineering methods in that it provides a more comprehensive output consisting of a tripartite network of expression similarity between genes, biological mechanisms and clinical phenotypes. Additionally, statistical significance is conducted over expression ordering inclusive of the complete array.

Trained on epigenetic mechanisms, PGnet accurately classified patients in the leukemia subtype and the relapse group, and these results suggest that a more comprehensive multi mechanism-based profile may achieve higher accuracy scores. The proposed method is scalable, in principle, to other mechanisms such as transcriptional networks, microRNA-regulated or Gene Ontology classes. In addition, the produced "similarity linkages" between mechanisms and genes comprise magnitude and direction (correlated or anti-correlated), which could also be utilize to infer regulation (activation or suppression) [15].

## Competing interests

The authors declare that they have no competing interests.

## Authors' contributions

Lussier was responsible of the overall experimental design and contributed to the R&D of the methods. Yang contributed significantly to the design, methods development and implementation, as well as the majority of the computations. Xie and Sun contributed to the design, methods and epigenetic gene lists. Yang, Huang, Chen, and Lussier contributed to the evaluation, discussion and conclusion.

## Supplementary Material

Additional file 1 Supplementary Methods – Supplementary Methods. Click here for file

Additional file 2 Supplementary Table 1 – The eight categories of epigenetic key terms and their corresponding 71 epigenetic seed genes. Click here for file

Additional file 3 Supplementary Table 2 – Thirty-three significant "LP-ESG" linkages contributed by corresponding GEMs (parameter T = 200) Click here for file

Additional file 4 Supplementary Table 3 – The predicted GEMs of 117 significant "LP-ESG" linkages based on optimal parameters (Ts) Click here for file

Additional file 5 Supplementary Figure 2 – The Design of the two Evaluations (A and B). Click here for file

Additional file 6 Supplementary Figure 3 – The ROC curve of the computational evaluation method B of PGnet-predicted GEMs and ESGs signature associated with ALL relapse. Click here for file

Additional file 7 Supplementary Figure 4 – The precision_recall results of the computational evaluation A of PGnet-predicted GEMs and ESGs signature associated with ALL relapse. Click here for file

Additional file 8 Supplementary Table 5 – Robust GEMs and ESGs Predictive of Leukemia Relapse. Click here for file

Additional file 9 Supplementary Table 4 – The significantly enriched GO items among the predicted GEMs that linking to phenotypes of interested. Click here for file

Additional file 10 Supplementary file Figure 5 – Expression pattern of ALL phenotype "Hyperdiploid>50" specific GEMs by PGnet. Click here for file

Additional file 11 Supplementary Table 6 – Previously reported facts about predicted GEMs. Click here for file

Additional file 12 Supplementary Table 7 – Comparison GEMs in PGnet with genes identified by ARACNE. Click here for file

Additional file 13 Supplementary Table 8 – Comparison of the results from ARACNE and PGnet for leukemia phenotypes. Click here for file

Additional file 14 Supplementary Figure 1 – PGnet: significant "phenotypes in ALL – GEMs – epigenetic seed genes" Click here for file
