# Supplementary material for: Mechanism-anchored profiling derived from epigenetic networks predicts outcome in acute lymphoblastic leukemia
Source: BMC Bioinformatics. 2009 Sep 17;10(Suppl 9):S6. doi: 10.1186/1471-2105-10-S9-S6 (PMC2745693; doi:10.1186/1471-2105-10-S9-S6)
Supplement: Additional file 1 — Supplementary Methods – Supplementary Methods. [file 1471-2105-10-S9-S6-S1.doc]

**Supplementary Methods**

# Dataset

We selected a well-studied public gene expression dataset[1] to check the global epigenetic protein behaviors in pediatric acute lymphoblastic leukemia (ALL). The leukemia phenotypes (LPs) in this dataset comprise of clinical subtypes, cytogenetic characteristics, molecular status and patient outcome. In this study, only the phenotypes with more than 3 samples were included which are: **i)** ALL genetic markers and subtypes, including t(1;19)(E2A-PBX1), t(12;21) (TEL-AML1), t(9;22) (BCR-ABL), leukemia with less diploid (Pseudodip), hypodiploid 47-50 (hypodip), more than 50 chromosomes (hyperdiploid>50), and with normal diploid (hereafter referred as “normal”). **ii)** The patient outcome phenotypes, including complete clinical remission (CCR), relapse and treatment-induced AML (2nd AML). The sample size in each phenotype is given in bellow **Table A**. Expression raw data were downloaded from: <http://www.stjuderesearch.org/data/ALL3> and the phenotypes of samples were downloaded from a previous study of the author[2]: [http://www.stjuderesearch.org/data/ALL1/all_section2.html#section2_top](http://www.stjuderesearch.org/data/ALL1/all_section2.html#section2_top).

**Table A. Leukemia phenotypes and sample sizes (n>3)**

| T-ALL | MLL | BCR-ABL | E2A-PBX1 | TEL-AML1 | Normal | Pseudodip | Hypodip | Hyperdip>50 | CCR | Relapse | 2ndAML |
| --- | --- | --- | --- | --- | --- | --- | --- | --- | --- | --- | --- |
| 14 | 20 | 13 | 18 | 20 | 10 | 11 | 4 | 18 | 71 | 16 | 6 |

# Finding Epigenetic Seed Genes (ESGs)

It has been proven *in vitro* that promoter methylation of regulatory genes can result in dys-expression of other genes and contribute to disease-specific phenotype[3]. To derive the epigenetic protein’s regulation of gene expression, we focused on genes whose translation products were associated with four major types of epigenetic modification by literature reviewing:

• ***DNA methylation****,* including 7 gene symbols: *MBD*[4], *SETDB*[4], *MECP*[4], *ZBTB33*[5], *DNMT*[6] and *SMARC*[7];

• ***genomic imprinting***, including *IGF2* [8] and genes in GO category “GO:0006349”;

•***histone modification****,* including 10 symbols of genes: *HAT1*[9], *DOT1L*[9], *MYST*[9], *HDACs*[8], *SUV39Hs*[10], SET[10], *EHMT* [10], *PRDMs*[10], *SMYD3*[11], *CBX*[12], “*histone acetyltransferase*” and *ash1*^[10]^ ;

• ***chromatin remodeling***: ***SWI/SNF***[13] enzymes and *BAZ2*[14].

A total of 17 symbols (**Suppl. Table1, column 2**) involving eight categories of epigenetic modification (**Suppl. Table1, column 1**) were used to search epigenetic seed genes in this ALL array data[1].

# Mapping Epigenetic Seed Genes to Affymatrix Probe-sets

The mapping between epigenetic seed genes and affymatrix probe-sets was based on LocusLink information and GO annotation (GO db 2.2.0), using the R-package[15] *hgu133a* (version 1.16.0). A total of 71 unique genes were found within the array (**Suppl. Table 1**).

# Preprocessing the Expression Profiles

The expression CEL files were scaled with asinh-transformation after variance stabilization and calibration normalization (vsn) [16], using Bioconductor package *compdiaglTools*[17]. The asinh-transformation is similar to the log2-transformation for large intensities, but it is less steep for low intensities.

We then applied the **inter quartile range (IQR)** filter[18] which eliminated genes leacking sufficient variation in expression across samples. The variation filter involved the following four steps: **1)** removing probe-sets whose average expression intensities were below than the average background intensities; **2)** removing probe-sets that could not be mapped to any Entrez gene ID, which resulted in 21,382 probe-sets; **3)** removing probe-sets with a IQR measurement lower than the median IQR values of remaining probe-sets, which resulted in 10,691 probe-sets; **4)** selecting the unique probe-sets for each gene (Entrez) with the largest IQR value, using the Bioconductor package *genefilter*. The last step reduced the number of our hypothesis testing to 7,256 genes, and improved the precision of test on microarray profile with fewer samples by eliminating thousands of variables. It precision improvement also due to that our “seed gene - phenotype” relation was derived from comparing two vectors (ordered gene lists), and the probe-sets for the same genes might perform similar and thus bias the whole compared vector, for example, all in the top ranks.

# Construction of the ESG-GEMs-LP Network

The construction of the gene-condition network required a series of five steps. **Step 1a**: After inputting the 48 out of 71 epigenetic seed genes (**ESGs**) that passed the IQR filter, the pairwise standard Pearson correlated coefficients of the gene expression levels between each gene in the array and every one of the 48 epigenetic genes were then calculated. Thus we got 48 vectors of co-expression coefficients *V_ESGi_, i*=1,2,…48. Note that the statistics of the probe-sets that designed for the same gene were not independently measured, but were measured by selecting the probe-set with the largest value of IQR for every gene. We did this selective measurement for each gene with multiple probe-sets because probes in Affymetrix chips do not work equally well, and we did not expect the measurement of the same gene bias the ranks on one hand or appear controversial up-/down-regulated on the other.

**Step 1b**: A one-vs.-all differential expression test was performed for each of the 12 leukemia phenotypes (**LPs**) with a sample size more than three. The “one-vs.-all” means after dividing the leukemia data into two sets based on whether they contained the phenotypic condition of interest, a two-group statistic was then calculated, which resulting in 12 vectors termed as *V_LPj_, j*=1,2,…12. Each of such vector was the adjusted t-statistics for differential expression in one LP. Note that only 93 samples had outcome information and were used when evaluating the differential expression in the conditions of outcome (Relapse, CCR and 2ndAML).

**Step 2**: Then a matrix of similarity scores *M_s_=(s_i,j_)* was calculated, where each score *s_i,j_* assesses the pair-wised similarities between the ordered co-expression coefficients and the ordered differential expression statistics. The similarity was tested based on a previous published algorithm[19, 20] which compares first order with second order, and with second reverse order as well. A preliminary similar score $s_{i,j}^{'}$ is given in formula 1:

$s_{i,j}^{'}=s'(V_{ESG_{i}},V_{LP_{j}})=\frac{1}{2}\sum w_{n}^{\alpha}[O_{n}(V_{ESG_{i}},V_{LP_{j}})+O_{n}(f(V_{ESG_{i}}),f(V_{LP_{j}}))]$ , (1)

where function $O_{n}$*(V_1_,V_2_)* counts the number of overlapping between two ordered gene lists (vectors) within their n leading orderings, and function *f* *(V)* flipps the vector *V* upside-down. The weight $w_{n}^{\alpha}=e^{-n\alpha}$ is (not necessarily strictly) decreasing along ranks *n* and hence how a partial intersection sizes on both ends of the orders can be controled by turning the parameter *α*[19].

The consequence enrichment analysis was performed not only for any pair of vectors (**straight similar**, the first half of the formula 2), but also for one vector with another reserved vector (**reversed similar**, the second half of the formula 2). This resulted in a total of two comparisons for each phenotype/seed gene combination. Thus the general delineation of similarity score *S_i,j_* considering about both ordering and reverse ordering is:

$s_{i,j}=s(V_{ESG_{i}},V_{LP_{j}})=\max(s'(V_{ESG_{i}},V_{LP_{j}}),s'(V_{ESG_{i}},f(V_{LP_{j}})))$ . (2)

If a “ESG_i_-LP_j_” pair was significantly straight similar, there must be a group of genes co-xpressed with seed genes (ESG) whose up-regulated genes in LP_j_ were enriched with the co-expressed genes with ESG_i_, and (or) whose down-regulated genes in LP_j_ were enriched with the anti-coexpressed genes with ESG_i_. Vice verse, for a reversed similarity, the enrichment were come between up-regulated genes in LP_j_ with the anti-coexpressed genes with ESG_i_, and (or) whose down-regulated genes in LP_j_ with the co-expressed genes with ESG_i_.

**Step 3**: **Vectorial Enrichment Optimization (VEO)**. After performing random permutations (permutation times were 2000) by randomly assigning the actual ranks to all genes, 1000 random scores were generated, and then an empirical p-value was calculated.

There were two parameters’ optimizations, one is the threshold of significance (*TS*), and another is the threshold of included ranks (*T*). A significance threshold (*TS* =0.01) was chosen to define significant similar pairs. The optimization of included ranks (*Opt.T*) was discussed in our previous publication[19], which is the one that achieves the lowest empirical p for a given range of candidates (100, 150, 200, 300, 400, 500, 750, 1000, 2000, 2500) based on 1000 random scores. The VOE is applied for each pair of vectors, and adjustment threshold of significance (*TS*) for vectorial similarity was conducted by controlling the false discovery rates.

Two networks were subsequently generated. The uniform significance threshold of included ranks (*T*=200) was chosen to define a highly significant score in VEO that would yield a small network consisting of 23 ESGs and 10 LPs (**Suppl. Table 2** and **Fig. 2 of the main document**). An unbiased data-driven optimized parameter leading to a larger network consisting of 46 ESGs and 11 LPs (**Suppl. Table 3**) were also generated. Q-value[21, 22] was calculated using the *Bioconductor* package *Qvalue* to estimate false discovery rate (proportion of false positives incurred) at each level of p-value to be considered as “significant”. The estimated q-values were given in **Suppl.** **Table 3**. Our analysis showed that a cut-off of q-value smaller than 0.02 would identify significant similar pairs of vectors at a zero false discovery rate (**Figure A**).

**Step 4**: For each significant pair of vectors that one corresponding to seed gene and another correspond to phenotype, we considered the epigenetic seed gene (ESG) and leukemia phenotype (LP) to be “linked.” For simplification, we hereinafter called the similarity between two ordered lists as a “similarity between the corresponding ESG of and LP”.

**Step 5**: Visualization. The hubs of our tripartite network are either epigenetic seed genes (triangle colored according to the ESG category) or leukemia phenotypes (box colored in yellow). The predicted epigenetically regulated genes (circle) were colored in grey. As mentioned in Step 2, formula 2, we compared the “straight similar” with “reversed similar” for any pair of vectors and took the maximum. If one vector significantly ordered as another vector, we linked them with magenta line while a turquoise line links the reversely ordered twos. In addition, every vertex of gene was colorful annotated according to its average change in expression to its linked phenotype (Red indicated up-regulation and blue for down-regulation). If a vertex of a gene had more than one linkage and was up-regulated in one condition but down-regulated in a different condition, it was annotated by grey. By color-coding the graph, in essence we are providing a direction to the similarity vectors. With these vectors, one can judge how these genes express in a specific condition. For example, in **Figure 2a of the main document**, the vertex of *HDAC9* is grey, as it is straight similar to the “Relapse” vertex and reversed similar to the “CCR” vertex. Correspondingly, gene *HDAC9* is up-regulated in the sample phenotype group as “Relapse”, but down-regulated in the CCR sample group.

**
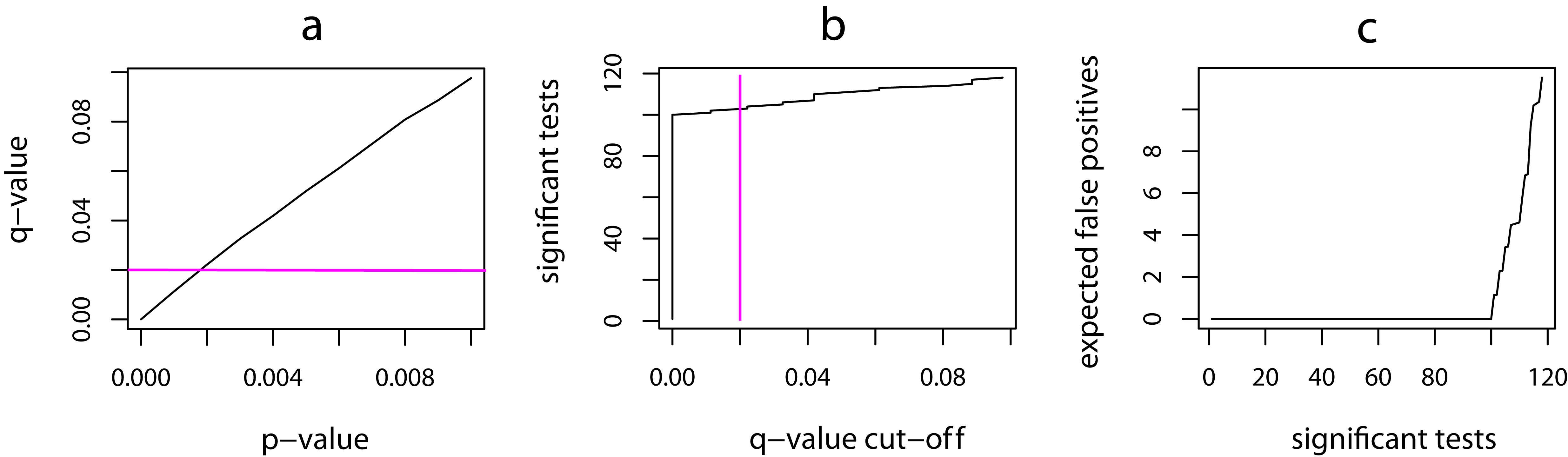
**

**Figure A.** Three plots describe the estimates q-values for empirical p-values. a) q-values versus the p-values; b) The number of significant tests versus each q-value cutoff; c) The number of expected false positives versus the number of significant tests. The red lines show that a cut-off of q-value smaller than 0.02 would identify none false discovery for our resulting pairs of vectors.

# Evaluation

**Table B: Two ways to evaluate PGnet**

| **Area of Evaluation** | **Method** |
| --- | --- |
| Examination of the putative gene functions predicted in the network | GO and PubMed databases were searched for corroboration of results in PGnet |
| Examination of the prognostic ability for partial profiling of the mechanism-anchored network by 100 iterations of three-fold cross-validation | **Evaluation A**: uses PGnet to identify predictors and trained linear Support Vector machine (SVM)[23] model to do classify (**Suppl. Fig. 2 panel a**). |
|  | **Evaluation B**: uses PGnet to identify predictors and Prediction Analysis for Microarrays Class Prediction (PAM)[24] as classifier (**Suppl. Fig. 2 panel b**). |

**Table B** provides a summary of our validation methods. To examine the putative gene functions predicted in the network, we searched PubMed resources. We also did hypergeometric evaluation for the GO item enrichment among the GEMs that linked to certain leukemia type. The background gene set was the genes detected by the chip (Affymetrix hgu133a.db_2.2.0). We used Bioconductor[25, 26] package *GOstats*[27] to do the enrichment analysis for molecular function terms in GO database (GO.db_2.2.0). And after further controlling the false discovery rate, the significant GO items were reported for genes linked to LP of interested (**Suppl. Table 4**).

To examine the prognostic ability for partial profiling of the epigenetic mechanism anchored network, 3-fold cross validation (CV) was run 100 repeats on the 87 samples with “relapse” or “continuous complete remission - CCR” following-up records in this dataset[1]. Please note that some patients did have an outcome of treatment-induced AML, a distinct form of leukemia, and were excluded here in the evaluation procession because this disease occurs at a later stage and the authors of the dataset did not disclose the outcome of ALL for these patients. Each running of CV adopted PGnet to select “CCR” associated features on two-thirds of random sampled stratified arrays and did classification on the remaining one-third arrays by either trained linear support vector machine[23] (SVM) model or directly running Prediction Analysis for Microarrays Class Prediction (PAM)[24]. The prediction accuracy was viewed by Receiver operating characteristic (ROC) curve and the precision-recall plots (Suppl. Fig 3 and Suppl. Fig 4).

To further assess the performance of PGnet feature selection on leukemia relapse prediction, we randomly picked the same number of genes as PGnet selected from 7,256 background genes, and observed the area under a curve (AUC) value for each cross-validation.

The ROC curve is widely used to direct-view the performance of any rule for two-group classifying problem. The sensitivity and specificity of validations therefore were plotted as ROC curve for each set of marker genes using Bioconductor package *ROC*. AUC is viewed as a measure of a prediction's accuracy, i.e. a measure of 1 would indicate a perfect model, while a measure of 0.5 would theoretically indicate a random prediction[28, 29]. The p-value of AUC were estimated using Bioconductor[25, 26] package *verification*[29]. And the 95% confidence intervals were estimated by calculating the sensitivity and specificity values after 1000 bootstrapping the observation and prediction.

There were 1,069 out of 7,256 genes were called at least once during the 100 iterations in Evaluation A. Among them, 55 genes including 3 ESGs (**Suppl. Table 5**) were high-frequently identified (x>32, the 95% quantitle of observed frequencies, **Figure B**) and hereafter were called as “robust features”[30].


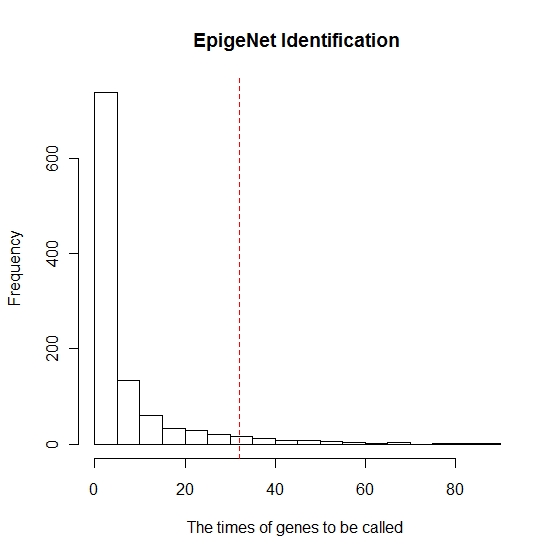


**Figure B.** Histogram of the frequencies of genes to be identified by PGnet during the 100 iterations of cross-validation (**Table B, Evaluation A**). The red dash line indicates the 95% quantitle of observed frequencies which is 32. The 55 robust signatures are the genes that were called more than 32 times (**Suppl. Table 5**).

# Comparison with Another Reverse Engineering Technology - ARACNE

ARACNE[31] is an approach for reverse engineering of cellular networks that overcomes the limitation of co-expression involved in indirect interactions[32], by using an Mutual Information (MI) theoretic approach, and has been shown effective in practice[33]. This method has been proven effective in identifying bona fide transcriptional targets in mammalian networks[33, 34]. We used ARACNE program for the reverse engineering of transcriptional networks from preprocessed microarray data and further identified a short range of direct interactions from the long range of correlated variables. For the expression levels of 132 leukemia samples, ARACENE determined a kernel width from the data size, the most important parameter for the Gaussian kernel estimator of MI which scaled as a power-law of the sample size, to be 0.499. As a result, our technique overlaps with ARACNE however has the added benefit of providing phenotypic information (results in **Suppl.** **Table 7**).

ARACNE is not designed to produce tripartite networks, while PGnet is. In other words, the ARACNE method allowed us to compare only the ESG-GEMs relationships, not the ESG-GEM-LP ones. We thus focused on the comparable components of the networks for the evaluation based on ARACNE. To further compare the two technologies in small sample size, we supplied ARACNE with our 48 epigenetic seed genes, and the expression data for samples of different phenotype, phenotype respectively (inputs). Thereafter, ARACNE produced 12 different phenotype specific gene-gene networks. We took the including criteria for MI as p-value<0.05, 15% DPI-tolerance and kept other default parameters of ARACNE and compared the results with PGnet (**Suppl. Table 8**).

# Reference

1. Ross ME, Zhou X, Song G, Shurtleff SA, Girtman K, Williams WK, Liu HC, Mahfouz R, Raimondi SC, Lenny N *et al*: **Classification of pediatric acute lymphoblastic leukemia by gene expression profiling**. *Blood* 2003, **102**(8):2951-2959.

2. Yeoh EJ, Ross ME, Shurtleff SA, Williams WK, Patel D, Mahfouz R, Behm FG, Raimondi SC, Relling MV, Patel A *et al*: **Classification, subtype discovery, and prediction of outcome in pediatric acute lymphoblastic leukemia by gene expression profiling**. *Cancer Cell* 2002, **1**(2):133-143.

3. Hopfer O, Komor M, Koehler IS, Schulze M, Hoelzer D, Thiel E, Hofmann WK: **DNA methylation profiling of myelodysplastic syndrome hematopoietic progenitor cells during in vitro lineage-specific differentiation**. *Exp Hematol* 2007, **35**(5):712-723.

4. Roloff TC, Ropers HH, Nuber UA: **Comparative study of methyl-CpG-binding domain proteins**. *BMC Genomics* 2003, **4**(1):1.

5. Kim SW, Park JI, Spring CM, Sater AK, Ji H, Otchere AA, Daniel JM, McCrea PD: **Non-canonical Wnt signals are modulated by the Kaiso transcriptional repressor and p120-catenin**. *Nat Cell Biol* 2004, **6**(12):1212-1220.

6. Hermann A, Gowher H, Jeltsch A: **Biochemistry and biology of mammalian DNA methyltransferases**. *Cell Mol Life Sci* 2004, **61**(19-20):2571-2587.

7. Moinova HR, Chen WD, Shen L, Smiraglia D, Olechnowicz J, Ravi L, Kasturi L, Myeroff L, Plass C, Parsons R *et al*: **HLTF gene silencing in human colon cancer**. *Proc Natl Acad Sci U S A* 2002, **99**(7):4562-4567.

8. Feinberg AP, Tycko B: **The history of cancer epigenetics**. *Nat Rev Cancer* 2004, **4**(2):143-153.

9. McManus KJ, Hendzel MJ: **Quantitative analysis of CBP- and P300-induced histone acetylations in vivo using native chromatin**. *Mol Cell Biol* 2003, **23**(21):7611-7627.

10. Kim KC, Geng L, Huang S: **Inactivation of a histone methyltransferase by mutations in human cancers**. *Cancer Res* 2003, **63**(22):7619-7623.

11. Hamamoto R, Furukawa Y, Morita M, Iimura Y, Silva FP, Li M, Yagyu R, Nakamura Y: **SMYD3 encodes a histone methyltransferase involved in the proliferation of cancer cells**. *Nat Cell Biol* 2004, **6**(8):731-740.

12. Bernstein E, Duncan EM, Masui O, Gil J, Heard E, Allis CD: **Mouse polycomb proteins bind differentially to methylated histone H3 and RNA and are enriched in facultative heterochromatin**. *Mol Cell Biol* 2006, **26**(7):2560-2569.

13. Roberts CW, Orkin SH: **The SWI/SNF complex--chromatin and cancer**. *Nat Rev Cancer* 2004, **4**(2):133-142.

14. Zhou Y, Santoro R, Grummt I: **The chromatin remodeling complex NoRC targets HDAC1 to the ribosomal gene promoter and represses RNA polymerase I transcription**. *EMBO J* 2002, **21**(17):4632-4640.

15. Ihaka R, Gentleman R: **R: a language for data analysis and graphics.** . *Journal of Computational and Graphical Statistics* 1996, **53** 299-314.

16. Huber W, von Heydebreck A, Sultmann H, Poustka A, Vingron M: **Variance stabilization applied to microarray data calibration and to the quantification of differential expression**. *Bioinformatics* 2002, **18 Suppl 1**:S96-104.

17. Scheid S. JJaLC: **A toolbox for performing and illustrating microarray data analyses – user’s guide to the R package compdiagTools.** *CompDiag Tech Rep* 2005, **01 Mar**.

18. von Heydebreck A, Huber W, Gentleman R: **Differential expression with the Bioconductor Project**. *Bioconductor Project Working Papers* 2004.

19. Yang X, Bentink S, Scheid S, Spang R: **Similarities of ordered gene lists**. *J Bioinform Comput Biol* 2006, **4**(3):693-708.

20. Lottaz C, Yang X, Scheid S, Spang R: **OrderedList--a bioconductor package for detecting similarity in ordered gene lists**. *Bioinformatics* 2006, **22**(18):2315-2316.

21. Storey J: **A direct approach to false discovery rates**. *Journal of the Royal Statistical Society, Series B* 2002, **64**:479-498.

22. Storey J, Taylor J, Siegmund D: **Strong control, conservative point estimation, and simultaneous conservative consistency of false discovery rates: A unified approach.** *Journal of the Royal Statistical Society, Series B* 2004, **66**:187-205.

23. Meye D, Leisch F, Hornik K: **The support vector machine under test.** . *Neurocomputing* 2003, **55**(1-2):169-186.

24. Tibshirani R, Hastie T, Narasimhan B, Chu G: **Diagnosis of multiple cancer types by shrunken centroids of gene expression**. *Proc Natl Acad Sci U S A* 2002, **99**(10):6567-6572.

25. Gentleman RC, Carey VJ, Bates DM, Bolstad B, Dettling M, Dudoit S, Ellis B, Gautier L, Ge Y, Gentry J *et al*: **Bioconductor: open software development for computational biology and bioinformatics**. *Genome Biol* 2004, **5**(10):R80.

26. R-Development-Core-Team: **R: A Language and Environment for Statistical Computing.** In: *R Foundation for Statistical Computing,: 2005; Vienna, Austria.*; 2005.

27. Alexa A, Rahnenfuhrer J, Lengauer T: **Improved scoring of functional groups from gene expression data by decorrelating GO graph structure**. *Bioinformatics* 2006, **22**(13):1600-1607.

28. Swets JA: **Theory and ROC Analysis in Psychology and Diagnostics.**: Lawrence Erlbaum Associates, Inc.; 1996.

29. Mason SJ, Graham NE: **Areas beneath the relative operating characteristics (ROC) and relative operating levels (ROL) curves: Statistical significance and interpretation**. *Q J R Meteorol Soc* 2002, **128**:2145-2166.

30. Michiels S, Koscielny S, Hill C: **Prediction of cancer outcome with microarrays: a multiple random validation strategy**. *Lancet* 2005, **365**(9458):488-492.

31. Margolin AA, Nemenman I, Basso K, Wiggins C, Stolovitzky G, Dalla Favera R, Califano A: **ARACNE: an algorithm for the reconstruction of gene regulatory networks in a mammalian cellular context**. *BMC Bioinformatics* 2006, **7 Suppl 1**:S7.

32. Ma S-K: **Statistical mechanics.** *Singapore: World Scientific* 1985.

33. Basso K, Margolin AA, Stolovitzky G, Klein U, Dalla-Favera R, Califano A: **Reverse engineering of regulatory networks in human B cells**. *Nat Genet* 2005, **37**(4):382-390.

34. Margolin AA, Wang K, Lim WK, Kustagi M, Nemenman I, Califano A: **Reverse engineering cellular networks**. *Nat Protoc* 2006, **1**(2):662-671.
