# Supplementary material for: Mechanism-anchored profiling derived from epigenetic networks predicts outcome in acute lymphoblastic leukemia
Source: BMC Bioinformatics. 2009 Sep 17;10(Suppl 9):S6. doi: 10.1186/1471-2105-10-S9-S6 (PMC2745693; doi:10.1186/1471-2105-10-S9-S6)
Supplement: Additional file 2 — Supplementary Table 1 – The eight categories of epigenetic key terms and their corresponding 71 epigenetic seed genes. [file 1471-2105-10-S9-S6-S2.doc]

**Supplementary Table 1**

**The eight categories of epigenetic key terms and their corresponding 71 epigenetic seed genes**

| **Biological Function or Process related to or targeted by Epigenetic Regulation** | | **Queried Biological Genes/Gene family**  **(Term used in Pubmed)** | **Associated Genes** | | | | | | | | | |  |
| --- | --- | --- | --- | --- | --- | --- | --- | --- | --- | --- | --- | --- | --- |
|  | Methyl-binding protein | MBD^1,2^ | **MBD1** | | **MBD2** | | **MBD3** | | **MBD4** | | MBD5 | |  |
|  |  | MECP2^1^ | **MECP2** | |  | |  | |  | |  | |  |
|  |  | ZBTB33^3,4^ | ZBTB33 | |  | |  | |  | |  | |  |
|  | DNA Methyltransferase | DNMT^2,5^ | **DNMT1** | | **DNMT3A** | | **DNMT3B** | | DNMT3L | |  | |  |
|  | Histon acetyltransferase | histone acetyltransferase**^6,7^** | **HAT1** | | **MYST1** | | **MYST2** | | **MYST3** | | **MYST4** | |  |
|  | Histone deacetylase | DOT1L^6^ | DOT1L | |  | |  | |  | |  | |  |
|  |  | HDAC^2,8^ | **HDAC1** | | HDAC11 | | **HDAC2** | | **HDAC3** | | **HDAC4** | |  |
|  |  |  | **HDAC5** | | **HDAC6** | | **HDAC7A** | | **HDAC9** | |  | |  |
|  | Histone methyltransferase | SETDB^1,9^ | **SETDB1** | |  | |  | |  | |  | |  |
|  |  | SUV39H^10,11^ | **SUV39H1** | | SUV39H2 | |  | |  | |  | |  |
|  |  | ash1^12^ | ASH1L | |  | |  | |  | |  | |  |
|  |  | PRDM^10,13,14^ | PRDM10 | | PRDM11 | | **PRDM12** | | PRDM13 | | PRDM14 | |  |
|  |  |  | **PRDM2** | | PRDM4 | | PRDM5 | | PRDM8 | | PRDM9 | |  |
|  |  |  | PRDM16 | |  | |  | |  | |  | |  |
|  |  | SMYD3^15^ | **SMYD3** | |  | |  | |  | |  | |  |
|  |  | EHMT^10,16^ | EHMT1 | | **EHMT2** | |  | |  | |  | |  |
|  | Histone modificated chromobox protein | CBX^11,17^ | **CBX1^11^** | | CBX2 | | **CBX3** | | **CBX4** | | **CBX5** | |  |
|  |  |  | **CBX6** | | **CBX7** | | CBX8 | |  | |  | |  |
|  | Imprinting | ** | KCNQ1 | | IGF2 | | **PHLDA2** | | **CTCF** | | DIRAS3 | |  |
|  | Chromatin remodeling | BAZ2^18^ | **BAZ2A** | | **BAZ2B** | |  | |  | |  | |  |
|  |  | SMARC^19,20^ | **SMARCA1** | | **SMARCA2** | | **SMARCA4** | | **SMARCA5** | | **SMARCAL1** | | |
|  |  |  | **SMARCB1** | | **SMARCC1** | | **SMARCC2** | | **SMARCD1** | | **SMARCD2** | | |
|  |  |  | SMARCD3 | **SMARCE1** | |  | |  | |  | |  |  |

Terms with quotation marks are used for gene name searching, ** were searched by GO group “imprinting” using “GO:0006349”. Forty-eight epigenetic seed genes (ESGs) with bold-face passed our IQR filtering. The second column gives the terms to be mapped; the third column lists the official gene symbols.

References:

1. Roloff TC, Ropers HH, Nuber UA. Comparative study of methyl-CpG-binding domain proteins. BMC Genomics. 2003 Jan 16;4(1):1.

2. Feinberg AP, Tycko B. The history of cancer epigenetics. Nat Rev Cancer. 2004 Feb;4(2):143-53.

3. Kim SW, Park JI, Spring CM, et al. Non-canonical Wnt signals are modulated by the Kaiso transcriptional repressor and p120-catenin. Nat Cell Biol. 2004 Dec;6(12):1212-20.

4. Filion GJ, Zhenilo S, Salozhin S, Yamada D, Prokhortchouk E, Defossez PA. A family of human zinc finger proteins that bind methylated DNA and repress transcription. Mol Cell Biol. 2006 Jan;26(1):169-81.

5. Hermann A, Gowher H, Jeltsch A. Biochemistry and biology of mammalian DNA methyltransferases. Cell Mol Life Sci. 2004 Oct;61(19-20):2571-87.

6. McManus KJ, Hendzel MJ. Quantitative analysis of CBP- and P300-induced histone acetylations in vivo using native chromatin. Mol Cell Biol. 2003 Nov;23(21):7611-27.

7. Georgiakaki M, Chabbert-Buffet N, Dasen B, et al. Ligand-controlled interaction of histone acetyltransferase binding to ORC-1 (HBO1) with the N-terminal transactivating domain of progesterone receptor induces steroid receptor coactivator 1-dependent coactivation of transcription. Mol Endocrinol. 2006 Sep;20(9):2122-40.

8. Bradbury CA, Khanim FL, Hayden R, et al. Histone deacetylases in acute myeloid leukaemia show a distinctive pattern of expression that changes selectively in response to deacetylase inhibitors. Leukemia. 2005 Oct;19(10):1751-9.

9. Schultz DC, Ayyanathan K, Negorev D, Maul GG, Rauscher FJ, 3rd. SETDB1: a novel KAP-1-associated histone H3, lysine 9-specific methyltransferase that contributes to HP1-mediated silencing of euchromatic genes by KRAB zinc-finger proteins. Genes Dev. 2002 Apr 15;16(8):919-32.

10. Kim KC, Geng L, Huang S. Inactivation of a histone methyltransferase by mutations in human cancers. Cancer Res. 2003 Nov 15;63(22):7619-23.

11. Garcia-Cao M, O'Sullivan R, Peters AH, Jenuwein T, Blasco MA. Epigenetic regulation of telomere length in mammalian cells by the Suv39h1 and Suv39h2 histone methyltransferases. Nat Genet. 2004 Jan;36(1):94-9.

12. Gregory GD, Vakoc CR, Rozovskaia T, et al. Mammalian ASH1L is a histone methyltransferase that occupies the transcribed region of active genes. Mol Cell Biol. 2007 Dec;27(24):8466-79.

13. Reid AG, Nacheva EP. A potential role for PRDM12 in the pathogenesis of chronic myeloid leukaemia with derivative chromosome 9 deletion. Leukemia. 2004 Jan;18(1):178-80.

14. Pastural E, Takahashi N, Dong WF, et al. RIZ1 repression is associated with insulin-like growth factor-1 signaling activation in chronic myeloid leukemia cell lines. Oncogene. 2007 Mar 8;26(11):1586-94.

15. Hamamoto R, Furukawa Y, Morita M, et al. SMYD3 encodes a histone methyltransferase involved in the proliferation of cancer cells. Nat Cell Biol. 2004 Aug;6(8):731-40.

16. Pless O, Kowenz-Leutz E, Knoblich M, et al. G9a-mediated lysine methylation alters the function of CCAAT/enhancer-binding protein-beta. J Biol Chem. 2008 Sep 26;283(39):26357-63.

17. Bernstein E, Duncan EM, Masui O, Gil J, Heard E, Allis CD. Mouse polycomb proteins bind differentially to methylated histone H3 and RNA and are enriched in facultative heterochromatin. Mol Cell Biol. 2006 Apr;26(7):2560-9.

18. Zhou Y, Santoro R, Grummt I. The chromatin remodeling complex NoRC targets HDAC1 to the ribosomal gene promoter and represses RNA polymerase I transcription. EMBO J. 2002 Sep 2;21(17):4632-40.

19. Moinova HR, Chen WD, Shen L, et al. HLTF gene silencing in human colon cancer. Proc Natl Acad Sci U S A. 2002 Apr 2;99(7):4562-7.

20. Pottier N, Yang W, Assem M, et al. The SWI/SNF chromatin-remodeling complex and glucocorticoid resistance in acute lymphoblastic leukemia. J Natl Cancer Inst. 2008 Dec 17;100(24):1792-803.
