# Supplementary material for: Mechanism-anchored profiling derived from epigenetic networks predicts outcome in acute lymphoblastic leukemia
Source: BMC Bioinformatics. 2009 Sep 17;10(Suppl 9):S6. doi: 10.1186/1471-2105-10-S9-S6 (PMC2745693; doi:10.1186/1471-2105-10-S9-S6)
Supplement: Additional file 3 — Supplementary Table 2 – Thirty-three significant "LP-ESG" linkages contributed by corresponding GEMs (parameter T = 200) [file 1471-2105-10-S9-S6-S3.doc]

**Supplementary Table 2**

**Thirty-three significant “LP-ESG” linkages and their corresponding GEMs (*orderedlist* parameter T=200)**

***LP:*** Leukemia Phenotype

***ESG***: Epigenetic Seed Gene

***P:***  The un-adjusted empirical vectorial enrichment p-value of every significant (p<0.001) “LP-ESG” pair

***Dir***: “+” represents straight similar, “-” represents reversed similar

***ESG.dys***: The average change of ESGs in expression to its linked leukemia phenotype compared to other phenotypes.

***Opt.T***: The optimal parameter T which achieves lowest empirical ***p*** for a given range of T candidates (100, 150, 200, 300, 400, 500, 750, 1000, 2000, 2500) based on 1000 random permutations of vectors.

***O(T)***: The counts of Genes co-Expressed with Mechanism genes (GEMs) using the corresponding optimal parameter T.

***O(200)***: The counts of GEMs using a parameter T=200.

***GEMs(200)***: The predicted GEMs using a parameter T=200.

| **LP** | **ESG** | **P** | **Dir** | **ESG.dys** | **Opt.T** | **O(T)** | **O(200)** | **GEMs(200)** |
| --- | --- | --- | --- | --- | --- | --- | --- | --- |
| Pseudodip | BAZ2A | <0.001 | + | up | 300 | 15 | 9 | SPIN2B; ADFP; FAM45B; TMEM164; TCEAL1; HNRPH2; PIN4; PCDH9; dJ222E13.2 |
| TEL-AML1 | DNMT3A | <0.001 | + | up | 200 | 25 | 25 | ADK; PTGES3; C22orf9; KIAA0564; ITM2C; C11orf24; ANXA2; PKIG; VGLL4; VAV1; KNTC1; SMAD1; RASA4; SCARB1; GNG11; RY1; CBFA2T3; PDLIM7; TNFRSF21; ABHD3; LOC654342; TCFL5; TNS1; C10orf26; ARHGEF4 |
| T-ALL | DNMT3B | <0.001 | + | up | 100 | 15 | 40 | CD74; HLA-DRA; HLA-DRB1; CD19; BLNK; HLA-DPA1; C14orf139; HLA-DPB1; HLA-DRB5; TCL1A; CD79A; POU2AF1; TFEB; CHD7; CTNNA1; HLA-DQB1; SLC27A3; PTPN18; BANK1; STX3; HLA-DRB6; PFTK1; CD9; PTK2; TRIM38; ENG; FHL1; STK32B; DSTN; ZNF167; SEMA4D; AGL; C9orf78; ATP1B1; C14orf135; LZTFL1; NUCB2; SHQ1; WDR67; TRD@ |
| T-ALL | HDAC4 | <0.001 | + | up | 100 | 19 | 56 | CD74; HLA-DRA; HLA-DRB1; BLNK; HLA-DPA1; HLA-DPB1; HLA-DMA; HLA-DRB5; CD79B; MEF2C; TCL1A; CD79A; SNX2; JUP; POU2AF1; TFEB; CHD7; GALNAC4S-6ST; HLA-DQB1; LAMC1; STX7; SLC27A3; PTPN18; HLA-DMB; BANK1; HLA-DRB6; PTK2; ENG; tcag7.1314; HLA-F; FHL1; STK32B; SLC25A15; INSIG1; DSTN; HIRA; ZAP70; NOTCH3; MLLT11; C9orf78; VAT1; PELO; C14orf135; LZTFL1; CD247; NUCB2; PEX5; CHI3L2; NBR1; USP20; LAT; BCL11B; LCK; UBASH3A; MAL; CD3D |
| MLL | HDAC9 | <0.001 | + | up | 100 | 23 | 51 | MGC29506; MME; MYO5C; SERPINB9; MYH10; FHIT; COL5A1; DPEP1; PARD3; SMAD1; VAMP5; HYI; GFOD1; ALOX5; POLE; NEDD4; SCHIP1; MDK; STK32B; GIMAP4; DDR1; MYO1B; FGD1; EDEM1; PTP4A3; ADK; PTGES3; TPP2; GLT8D1; STS; ADAM10; C22orf9; GALC; ZEB2; IGFBP7; LY75; WIPI1; CD72; SIDT2; HK2; C11orf24; CD44; FEZ2; FAIM; NLRP3; MPZL1; PLXNC1; ATP8B4; LGALS1; KLRK1; C20orf103 |
| Hyperdip>50 | HDAC6 | <0.001 | + | up | 300 | 41 | 25 | RAG2; ABCC4; AKAP12; PQBP1; ARMCX5; CXorf45; UBE2A; ABCD4; ATP6AP2; MAGEH1; LAS1L; RRP1B; GPKOW; MORC3; HNRPH2; ABCB7; UBQLN2; SLC9A6; FTSJ1; USP9X; RNF113A; MED12; RP6-213H19.1; PSMD10; UPF3B |
| MLL | SMARCA2 | <0.001 | + | up | 300 | 60 | 31 | MGC29506; MYH10; DPEP1; AKAP12; PARD3; ITPR3; MAGED1; EMP2; STAT4; RASA1; PABPC4; MED13L; SMARCA2; ZNF394; SIRT7; TSEN34; SH3YL1; ARL6IP5; MAN2B1; ZEB2; OXA1L; LY75; AK2; CDKN1B; FUT4; C1orf164; FEZ2; PCDHGC3; DAD1; PLXNC1; IGF2BP2 |
| T-ALL | SMYD3 | <0.001 | + | up | 100 | 10 | 17 | CD74; HLA-DRA; HLA-DRB1; HLA-DPA1; HLA-DPB1; HLA-DMA; HLA-DRB5; CD79B; CD24; TCL1A; CD79A; POU2AF1; CHD7; HLA-DQB1; SLC27A3; HLA-DRB6; tcag7.1314 |
| E2A-PBX1 | PHLDA2 | <0.001 | + | up | 200 | 34 | 34 | EVI2A; SOCS2; CCND2; PLSCR1; ARHGAP4; GUSB; LTB4R; HPCAL1; TMEM134; XBP1; TUSC4; SH3BP4; RHOBTB1; SLC15A2; FNDC3B; CALD1; APBB2; EAF2; PLEKHF2; ELL3; ROR1; ADARB1; DACT1; KCNJ12; GOLGA3; FHOD3; SEMA4C; GP5; HIP1R; NID2; KIAA0802; SYNPO; SLC27A2; PBX1 |
| Relapse | HDAC9 | <0.001 | + | up | 400 | 10 | 5 | MYH10; SALL2; CEBPE; LGALS1; IGFBP7 |
| TEL-AML1 | MBD2 | <0.001 | - | down | 300 | 47 | 20 | FAM120A; MBD2; MRCL3; DSC2; DUOX1; ANGPTL2; DLGAP2; SEMA3F; DRAM; PTPRK; KCNK3; GBA3; TMEM16A; LOC654342; NOVA1; HAP1; FBN2; PCLO; BIRC7; ARHGEF4 |
| Hyperdip>50 | BAZ2A | <0.001 | - | down | 150 | 21 | 27 | ELF1; ZNF75; ARMCX5; ASB9; ENOX2; UBE2A; ATP6AP2; MGC39900; MORC3; HNRPH2; PRKAR2B; WDR44; SOD1; SLC9A6; SETD3; FTSJ1; ARMCX1; MTCP1; PIGP; LOC57228; DHRS4; RP6-213H19.1; CRYZL1; PSMD10; TCEAL1; TCEAL4; UPF3B |
| E2A-PBX1 | BAZ2B | <0.001 | - | down | 100 | 25 | 45 | GNAQ; PRKACB; KIAA0430; SNAP23; AKAP11; STX16; GNPTAB; HSD17B11; TTC31; SLC35E2; LETMD1; F13A1; LTBP2; DOCK9; PSAT1; ODZ4; QRSL1; GNAZ; KCNA3; TMEM121; RASAL1; ELOVL2; CCDC81; IL12RB2; IGSF3; KCNMB3; ELL3; IRF4; ROR1; SAMD4A; KCNJ12; FAT; NCAPD3; FHOD3; SORBS1; SEMA4C; GP5; HIP1R; NID2; PRKCZ; KIAA0802; SYNPO; PSEN2; MERTK; PBX1 |
| E2A-PBX1 | MECP2 | <0.001 | - | down | 150 | 22 | 33 | ZMYND11; CTDSP2; ARHGAP4; SLC35E2; STAT5B; EXOC1; ARNTL2; MAP3K1; MAP1B; FGF9; GPR176; ENDOD1; SYT1; CCDC81; IL12RB2; APBB2; EAF2; KCNMB3; BLK; IRF4; AOX1; ROR1; SAMD4A; LRMP; KANK1; DACT1; FHOD3; SLAMF1; GP5; SYNPO; PSEN2; SLC27A2; PBX1 |
| BCR-ABL | DNMT3B | <0.001 | - | down | 300 | 30 | 12 | C5orf13; GLUL; JARID1B; MVP; STX3; GIMAP4; S100A13; MS4A1; RAPGEF3; TBXA2R; ENG; ECM1 |
| T-ALL | HDAC5 | <0.001 | - | down | 100 | 25 | 60 | PAX5; C14orf139; MEF2C; BTK; SNX2; JUP; PLCG2; CIITA; NCF4; TFEB; IGHM; CTNNA1; MSRA; HLX; LAPTM5; NUBP1; PLXNB2; ROGDI; MARCH3; SIPA1; LRP10; ST18; ZNF167; LIMA1; RGS10; SNX10; SLC19A2; ITM2A; CMAH; ATP1B1; PTPN22; CD2; GATA3; FXYD2; AKR1C3; GALNT6; NGFRAP1; STAU2; ITK; DENND2D; CHI3L2; CD28; TFDP2; AQP3; IL23A; PTPN7; BIN2; LAT; BCL11B; PRKCQ; LCK; SCD; CD7; TRAT1; UBASH3A; CD3E; TRD@; MAL; SH2D1A; CD3D |
| BCR-ABL | HDAC4 | <0.001 | - | down | 300 | 28 | 15 | GLUL; PKIA; TUSC4; HDAC4; ITGAE; GIMAP4; PALM; FLJ20489; CCND2; S100A13; P2RY14; RAPGEF3; ENG; ECM1; SLC2A5 |
| TEL-AML1 | HDAC9 | <0.001 | - | down | 100 | 15 | 47 | ADK; PTGES3; SORD; IQGAP2; C22orf9; PUS7; CD44; PTGER4; MINA; ATP8A1; C11orf24; MPZL1; FLJ11184; FAM98A; CTSC; FXN; EDEM1; GABBR1; CD27; ZNF91; AJAP1; SMAD1; FARP1; FERMT2; SEMA6A; SH3GLB2; H1F0; GNG11; SPTA1; DSG2; MDK; PTP4A3; RAG1; NRN1; TSPYL5; TERF2; NR3C2; SPANXA1; KCNK3; TUSC3; ARHGAP29; TNFRSF21; LOC654342; NOVA1; HAP1; PCLO; ARHGEF4 |
| CCR | HDAC9 | <0.001 | - | down | 200 | 13 | 13 | IGFBP7; LGALS1; WIPI1; ATP8B4; TBK1; NLRP3; MYO1F; PTGES3; HK2; FEZ2; POLE; NUDT11; SALL2 |
| E2A-PBX1 | HDAC7 | <0.001 | - | down | 100 | 20 | 38 | GNAQ; CD99; CTDSP2; LASP1; ITGB1; PRKCB1; AMZ2; STX16; GPSM3; SLC35E2; DGAT1; C14orf1; TMC6; TMEM134; F13A1; GALNT14; SYT1; RASAL1; APBB2; EAF2; ELL3; BLK; IRF4; AOX1; ROR1; SAMD4A; LRMP; KANK1; DACT1; NP; FHOD3; SLAMF1; GP5; PRKCZ; SYNPO; PSEN2; MERTK; PBX1 |
| E2A-PBX1 | SMARCA2 | <0.001 | - | down | 300 | 49 | 30 | HMHA1; TM9SF3; SNAP23; AKAP11; JMJD1C; HSD17B11; CNDP2; METTL7A; LETMD1; NFATC3; EXOC1; EMP2; MAGED1; ARNTL2; LTBP2; DOCK9; CASC1; PSAT1; KCNA3; ALDH1A1; ELOVL2; ROR1; FAT; FHOD3; SLAMF1; HIP1R; NID2; PRKCZ; PSEN2; PBX1 |
| Hyperdip>50 | SMARCA4 | <0.001 | - | down | 200 | 17 | 17 | COMMD4; ANP32A; BCL2L1; SMARCA4; SPTBN1; PSME4; HDGF; TLR2; ECHDC3; IL6R; ALDH3B1; ITSN1; MS4A6A; PLP2; IL13RA1; IL3RA; ZNF185 |
| Normal | SUV39H1 | <0.001 | - | down | 300 | 39 | 23 | KIAA0101; TYMS; HMGN2; SMS; RBBP7; UCHL5IP; UBE2A; CDC2; TBC1D25; SOD1; CBLB; DLGAP4; CD84; ZNF192; CSHL1; ITGA6; FLT1; PCDH9; AGMAT; MPPED2; HEY2; LHFPL2; STAP1 |
| Relapse | SUV39H1 | <0.001 | - | down | 200 | 22 | 22 | SHCBP1; NCAPH; TOP2A; ARHGAP19; KIF4A; SPAG5; CCNB2; CDC45L; C21orf45; BIRC5; KIF11; PLK4; TIMELESS; MAD2L1; AURKB; KIF2C; H2AFZ; CKS1B; ZWINT; RAD51; NPR3; FLJ13197 |
| T-ALL | PRDM2 | <0.001 | - | down | 200 | 30 | 30 | CD24; CD79A; POU2AF1; INSR; CUL1; GNG7; AUTS2; TRAK1; MLXIP; PCBP3; SLC9A3R1; FAH; LIME1; PELO; EPHB6; PEX5; GATA3; FXYD2; AKR1C3; GALNT6; NGFRAP1; TFDP2; AQP3; LAT; TRAT1; UBASH3A; CD3E; MAL; SH2D1A; CD3D |
| BCR-ABL | CBX1 | <0.001 | - | down | 100 | 8 | 25 | PKIA; HDAC4; RABL4; SERPINB6; GIMAP4; PALM; HPCAL1; FLJ20489; ITGA5; CCND2; S100A13; P2RY14; RAPGEF3; ARHGEF17; TBXA2R; ASB13; LIMD1; BAALC; PRX; ENG; TBX21; ECM1; SLC2A5; LOC26010; OLFML2A |
| E2A-PBX1 | CBX6 | <0.001 | - | down | 200 | 21 | 21 | CIRBP; CTDSP2; ARHGAP4; SAPS2; DGAT1; TMEM134; CASC1; SLC15A2; ALDH1A1; RASAL1; CCDC81; APBB2; EAF2; SAMD4A; KANK1; DACT1; GP5; SYNPO; PSEN2; SLC27A2; PBX1 |
| Normal | CBX5 | <0.001 | - | down | 300 | 35 | 16 | NUCKS1; KIAA0101; TYMS; SS18L2; CDC2; PTTG1; FH; TP53TG3; CCR5; CSHL1; REEP1; FLT1; FER1L3; SLC6A16; AGMAT; GFOD1 |
| Relapse | CBX5 | <0.001 | - | down | 100 | 9 | 17 | TUBB; NCAPH; TOP2A; MKI67; TPX2; ANP32E; KIF20A; CCNB2; BUB1; CCNA2; AURKB; KIF2C; H2AFZ; CKS1B; ZWINT; NPR3; FLJ13197 |
| Hyperdip>50 | CBX7 | 0.004 | - | down | 400 | 26 | 7 | ECHDC3; ITSN1; MYBPC2; C10orf56; IL13RA1; IL3RA; ZNF185 |
| E2A-PBX1 | MYST2 | <0.001 | - | down | 300 | 43 | 26 | TMED10; MORC3; SEL1L; EMP2; PRR5; PPFIA4; ARNTL2; SGSM3; EWSR1; GPR176; ODZ4; QRSL1; GNAZ; ALDH1A1; RASAL1; KCNMB3; ROR1; DACT1; KCNJ12; FAT; NID2; PRKCZ; SYNPO; PSEN2; MERTK; PBX1 |
| E2A-PBX1 | MYST4 | <0.001 | - | down | 100 | 10 | 19 | ZMYND11; ARNTL2; GPR176; PSAT1; QRSL1; TMEM121; RASAL1; CCDC81; KCNMB3; IRF4; AOX1; ROR1; SAMD4A; DACT1; SLAMF1; NID2; SYNPO; PSEN2; PBX1 |
| Relapse | DNMT3A | <0.001 | - | down | 750 | 23 | 6 | DBN1; TUBB; SEPHS1; ZNF675; PRPF4B; S100A4 |
