# Supplementary material for: Mechanism-anchored profiling derived from epigenetic networks predicts outcome in acute lymphoblastic leukemia
Source: BMC Bioinformatics. 2009 Sep 17;10(Suppl 9):S6. doi: 10.1186/1471-2105-10-S9-S6 (PMC2745693; doi:10.1186/1471-2105-10-S9-S6)
Supplement: Additional file 4 — Supplementary Table 3 – The predicted GEMs of 117 significant "LP-ESG" linkages based on optimal parameters (Ts) [file 1471-2105-10-S9-S6-S4.doc]

**Supplementary Table 3**

**The predicted GEMs of 117 significant “LP-ESG” linkages based on optimal Ts**

**--- 102 linkages with p-value smaller than 0.02 were estimated to be none-false discovery (Suppl. Methods)**

***LP:*** Leukemia Phenotype

***ESG***: Epigenetic Seed Gene

***p:***  The un-adjusted empirical vectorial enrichment p-value of every significant (p<0.001) “LP-ESG” pair

***q:*** The estimated false discovery rate (proportion of false positives incurred) at given empirical p-value threshold.

***Dir***: “+” represents straight similar, “-” represents reversed-similar

***ESG.dys***: The average change of ESGs in expression to its linked leukemia phenotype compared to other phenotypes.

***Opt.T***: The optimal parameter T which achieves lowest empirical ***p*** for a given range of T candidates (100, 150, 200, 300, 400, 500, 750, 1000, 2000, 2500) based on 1000 random permutations of vectors.

***O(T)***: The counts of Genes co-Expressed with Mechanism genes (GEMs) using the corresponding optimal parameter T.

***GEMs(T)***: The predicted GEMs using the corresponding optimal parameter T.

| **LP** | **ESG** | **p** | **q** | **Dir** | **ESG.dys** | **Opt.T** | **O(T)** | **GEMs(T)** |
| --- | --- | --- | --- | --- | --- | --- | --- | --- |
| Pseudodip | BAZ2A | <0.001 | 0 | + | up | 300 | 15 | SPIN2B; ALG13; ADFP; RP2; ARFIP1; FAM45B; TMEM164; LAMP2; TCEAL1; HNRPH2; PIN4; GLT25D1; SH2B2; PCDH9; dJ222E13.2 |
| TEL-AML1 | DNMT3A | <0.001 | 0 | + | up | 200 | 25 | ADK; PTGES3; C22orf9; KIAA0564; ITM2C; C11orf24; ANXA2; PKIG; VGLL4; VAV1; KNTC1; SMAD1; RASA4; SCARB1; GNG11; RY1; CBFA2T3; PDLIM7; TNFRSF21; ABHD3; LOC654342; TCFL5; TNS1; C10orf26; ARHGEF4 |
| T-ALL | DNMT3B | <0.001 | 0 | + | up | 100 | 15 | CD74; HLA-DRA; HLA-DRB1; BLNK; HLA-DPA1; HLA-DPB1; HLA-DRB5; TCL1A; TFEB; CTNNA1; HLA-DQB1; SLC27A3; NUCB2; SHQ1; WDR67 |
| T-ALL | HDAC4 | <0.001 | 0 | + | up | 100 | 19 | CD74; HLA-DRA; HLA-DRB1; BLNK; HLA-DPA1; HLA-DPB1; HLA-DMA; HLA-DRB5; JUP; CHD7; HLA-DQB1; STX7; SLC27A3; NUCB2; CHI3L2; LAT; BCL11B; LCK; MAL |
| MLL | HDAC9 | <0.001 | 0 | + | up | 100 | 23 | MGC29506; MME; MYH10; FHIT; COL5A1; DPEP1; PARD3; SMAD1; VAMP5; HYI; GFOD1; ALOX5; POLE; SCHIP1; WIPI1; HK2; C11orf24; FEZ2; FAIM; NLRP3; PLXNC1; LGALS1; C20orf103 |
| Hyperdip>50 | HDAC6 | <0.001 | 0 | + | up | 300 | 41 | OR7E38P; RAG2; ABCC4; TPD52; AKAP12; MCTP2; PCDH9; TTC3; PDHA1; SH3BGRL; HDAC6; CETN2; STAG2; HUWE1; TMEM164; PHKA2; CUL4B; PQBP1; ARMCX5; CXorf45; UBE2A; ABCD4; ATP6AP2; MAGEH1; LAS1L; RRP1B; GPKOW; MORC3; HNRPH2; ABCB7; UBQLN2; SLC9A6; FTSJ1; USP9X; RNF113A; MED12; RP6-213H19.1; CRYZL1; PSMD10; TCEAL1; UPF3B |
| MLL | SMARCA2 | <0.001 | 0 | + | up | 300 | 60 | MGC29506; MME; CD52; EXDL2; MYH10; DPEP1; AKAP12; PARD3; ITPR3; MAGED1; HYI; DBN1; EMP2; FGD1; BMPR2; STAT4; ZNF415; SALL2; ZNF667; ODZ4; TREML2; SPAG16; RAPGEF3; COPS7A; RNFT1; DVL1; EP400; PHF6; MYH9; RPS6KA1; IKBKB; PLXNB2; JMJD1C; RASA1; PABPC4; MED13L; SMARCA2; ZNF394; COQ2; MAP4; RPP40; SIRT7; TSEN34; SH3YL1; ARL6IP5; MAN2B1; ZEB2; OXA1L; LY75; AK2; CDKN1B; FUT4; C1orf164; ADCY9; FEZ2; FAIM; PCDHGC3; DAD1; PLXNC1; IGF2BP2 |
| T-ALL | SMYD3 | <0.001 | 0 | + | up | 100 | 10 | CD74; HLA-DRA; HLA-DRB1; HLA-DPA1; HLA-DPB1; HLA-DMA; HLA-DRB5; TCL1A; POU2AF1; SLC27A3 |
| E2A-PBX1 | PHLDA2 | <0.001 | 0 | + | up | 200 | 34 | EVI2A; SOCS2; CCND2; PLSCR1; ARHGAP4; GUSB; LTB4R; HPCAL1; TMEM134; XBP1; TUSC4; SH3BP4; RHOBTB1; SLC15A2; FNDC3B; CALD1; APBB2; EAF2; PLEKHF2; ELL3; ROR1; ADARB1; DACT1; KCNJ12; GOLGA3; FHOD3; SEMA4C; GP5; HIP1R; NID2; KIAA0802; SYNPO; SLC27A2; PBX1 |
| Relapse | HDAC9 | <0.001 | 0 | + | up | 400 | 10 | DBN1; HMGB3; MYH10; SALL2; CEBPE; LGALS1; IGFBP7; MFSD1; S100A4; AHR |
| TEL-AML1 | MBD2 | <0.001 | 0 | - | down | 300 | 47 | NME2; FAM120A; CCDC28A; VIM; MBD2; MRCL3; ACAT2; FDX1; ME2; SRP72; ARHGAP24; NOL4; PRO2268; GPR17; DSC2; DUOX1; CACNB2; SPON1; MSR1; AJAP1; FERMT2; EHD2; TFPI; ANGPTL2; DLGAP2; MAPK13; MDK; TRPM4; SEMA3F; CXCR7; DRAM; PTPRK; MYO10; SPANXA1; KCNK3; TUSC3; GBA3; TMEM16A; LOC654342; NOVA1; HAP1; FBN2; TNS1; PCLO; BIRC7; CLIC5; ARHGEF4 |
| Hyperdip>50 | BAZ2A | <0.001 | 0 | - | down | 150 | 21 | ATP6AP2; MGC39900; MORC3; HNRPH2; PRKAR2B; WDR44; SOD1; SLC9A6; SETD3; FTSJ1; ARMCX1; MTCP1; PIGP; LOC57228; DHRS4; RP6-213H19.1; CRYZL1; PSMD10; TCEAL1; TCEAL4; UPF3B |
| E2A-PBX1 | BAZ2B | <0.001 | 0 | - | down | 100 | 25 | GNAQ; PRKACB; AKAP11; STX16; GNPTAB; TMEM121; RASAL1; ELOVL2; CCDC81; IL12RB2; IGSF3; KCNMB3; ELL3; IRF4; ROR1; SAMD4A; KCNJ12; FAT; FHOD3; HIP1R; NID2; PRKCZ; SYNPO; MERTK; PBX1 |
| E2A-PBX1 | MECP2 | <0.001 | 0 | - | down | 150 | 22 | CTDSP2; ARHGAP4; SLC35E2; MAP1B; SYT1; CCDC81; IL12RB2; APBB2; EAF2; BLK; IRF4; AOX1; ROR1; SAMD4A; KANK1; DACT1; FHOD3; SLAMF1; GP5; SYNPO; PSEN2; PBX1 |
| BCR-ABL | DNMT3B | <0.001 | 0 | - | down | 300 | 30 | C5orf13; GLUL; ITGAE; JARID1B; HK2; IGFBP4; MYO5C; PTPN18; NAV1; SLC35D2; SCHIP1; SCN1B; CAST; MVP; STX3; GIMAP4; HPCAL1; MTSS1; SH2B3; S100A13; TNFRSF1B; MS4A1; RAPGEF3; EMP1; TBXA2R; SEMA6A; ENG; TBX21; ECM1; OLFML2A |
| T-ALL | HDAC5 | <0.001 | 0 | - | down | 100 | 25 | PAX5; C14orf139; MEF2C; BTK; CIITA; NCF4; TFEB; IGHM; CTNNA1; GATA3; FXYD2; NGFRAP1; ITK; TFDP2; AQP3; IL23A; PTPN7; CD7; TRAT1; UBASH3A; CD3E; TRD@; MAL; SH2D1A; CD3D |
| BCR-ABL | HDAC4 | <0.001 | 0 | - | down | 300 | 28 | GLUL; PKIA; TUSC4; HDAC4; ITGAE; MYO5C; PTPN18; NAV1; SLC35D2; SCHIP1; LUZP1; DUSP6; SERPINB6; STX3; GIMAP4; PALM; FLJ20489; CCND2; S100A13; OSBPL10; P2RY14; RAPGEF3; SEMA6A; LIMD1; ENG; ECM1; SLC2A5; OLFML2A |
| TEL-AML1 | HDAC9 | <0.001 | 0 | - | down | 100 | 15 | ADK; PTGES3; IQGAP2; C22orf9; PUS7; MINA; ATP8A1; C11orf24; DSG2; PTP4A3; TSPYL5; TERF2; TUSC3; ARHGAP29; TNFRSF21 |
| CCR | HDAC9 | <0.001 | 0 | - | down | 200 | 13 | IGFBP7; LGALS1; WIPI1; ATP8B4; TBK1; NLRP3; MYO1F; PTGES3; HK2; FEZ2; POLE; NUDT11; SALL2 |
| E2A-PBX1 | HDAC7 | <0.001 | 0 | - | down | 100 | 20 | CD99; CTDSP2; LASP1; ITGB1; SYT1; APBB2; EAF2; ELL3; BLK; IRF4; SAMD4A; LRMP; KANK1; DACT1; NP; FHOD3; GP5; SYNPO; PSEN2; PBX1 |
| E2A-PBX1 | SMARCA2 | <0.001 | 0 | - | down | 300 | 49 | HMHA1; SNX3; TM9SF3; SNAP23; AKAP11; JMJD1C; GALNT1; RALB; HSD17B11; CNDP2; METTL7A; LETMD1; NFATC3; RNF13; UBE4A; EXOC1; TMEM30A; B3GNT2; OS9; TMEM43; ARHGEF11; MYT1L; MYL4; PAWR; ARL4C; EMP2; MAGED1; ARNTL2; LTBP2; DOCK9; CASC1; PSAT1; ODZ4; D4S234E; KCNA3; ALDH1A1; KIAA0922; ELOVL2; ROR1; FAT; FHOD3; SLAMF1; GP5; HIP1R; NID2; PRKCZ; PSEN2; MERTK; PBX1 |
| Hyperdip>50 | SMARCA4 | <0.001 | 0 | - | down | 200 | 17 | COMMD4; ANP32A; BCL2L1; SMARCA4; SPTBN1; PSME4; HDGF; TLR2; ECHDC3; IL6R; ALDH3B1; ITSN1; MS4A6A; PLP2; IL13RA1; IL3RA; ZNF185 |
| Normal | SUV39H1 | <0.001 | 0 | - | down | 300 | 39 | APITD1; TRMT5; KIAA0101; TYMS; LAGE3; HMGN2; SMS; RBBP7; UCHL5IP; UBE2A; CDC2; TBC1D25; ATP5J; HMGN1; SOD1; CCNB2; MCM6; SLC25A5; EIF4A3; EBP; H2AFZ; TOP2A; PSMG1; CKS1B; C4orf27; CBLB; DLGAP4; CD84; ZNF192; CSHL1; ITGA6; FLT1; PCDH9; FER1L3; AGMAT; MPPED2; HEY2; LHFPL2; STAP1 |
| Relapse | SUV39H1 | <0.001 | 0 | - | down | 200 | 22 | SHCBP1; NCAPH; TOP2A; ARHGAP19; KIF4A; SPAG5; CCNB2; CDC45L; C21orf45; BIRC5; KIF11; PLK4; TIMELESS; MAD2L1; AURKB; KIF2C; H2AFZ; CKS1B; ZWINT; RAD51; NPR3; FLJ13197 |
| T-ALL | PRDM2 | <0.001 | 0 | - | down | 200 | 30 | CD24; CD79A; POU2AF1; INSR; CUL1; GNG7; AUTS2; TRAK1; MLXIP; PCBP3; SLC9A3R1; FAH; LIME1; PELO; EPHB6; PEX5; GATA3; FXYD2; AKR1C3; GALNT6; NGFRAP1; TFDP2; AQP3; LAT; TRAT1; UBASH3A; CD3E; MAL; SH2D1A; CD3D |
| BCR-ABL | CBX1 | <0.001 | 0 | - | down | 100 | 8 | PKIA; HDAC4; TBXA2R; LIMD1; ENG; ECM1; SLC2A5; OLFML2A |
| E2A-PBX1 | CBX6 | <0.001 | 0 | - | down | 200 | 21 | CIRBP; CTDSP2; ARHGAP4; SAPS2; DGAT1; TMEM134; CASC1; SLC15A2; ALDH1A1; RASAL1; CCDC81; APBB2; EAF2; SAMD4A; KANK1; DACT1; GP5; SYNPO; PSEN2; SLC27A2; PBX1 |
| Normal | CBX5 | <0.001 | 0 | - | down | 300 | 35 | NUCKS1; KIAA0101; TYMS; SS18L2; CDC2; PTTG1; FH; CCNB2; TUBA1C; MCM6; RPA3; RFC4; KIF18A; H2AFZ; NDUFA6; CBX5; TOP2A; NDUFS6; CKS1B; RAN; SFRS2B; TUBB2C; AMT; TP53TG3; CCR5; EFR3B; PRKG2; ZNF239; CSHL1; REEP1; FLT1; FER1L3; SLC6A16; AGMAT; GFOD1 |
| Relapse | CBX5 | <0.001 | 0 | - | down | 100 | 9 | TUBB; NCAPH; MKI67; TPX2; KIF20A; CCNB2; BUB1; NPR3; FLJ13197 |
| Hyperdip>50 | CBX7 | 0.004 | 0.04189 | - | down | 400 | 26 | TTC15; ADAM17; SENP7; USP4; DLEU2L; MPHOSPH8; MZF1; REPIN1; ARID4B; VEGFB; POLA1; PET112L; RRP1; PRKAR2B; ECHDC3; IL6R; CYB5A; SETD3; ALDH3B1; ITSN1; MYBPC2; PLP2; C10orf56; IL13RA1; IL3RA; ZNF185 |
| E2A-PBX1 | MYST2 | <0.001 | 0 | - | down | 300 | 43 | MSN; TMED10; MORC3; SEL1L; TMEM30A; ZFP106; CLINT1; ATP6AP2; YPEL1; TCF3; MYL4; JAG1; JAM2; KCNJ16; EPB41L2; EMP2; PRR5; PPFIA4; ARNTL2; PHACTR1; SGSM3; EWSR1; GPR176; ODZ4; QRSL1; GNAZ; ALDH1A1; CALD1; RASAL1; KCNMB3; ROR1; DACT1; KCNJ12; FAT; SLAMF1; HIP1R; NID2; PRKCZ; KIAA0802; SYNPO; PSEN2; MERTK; PBX1 |
| E2A-PBX1 | MYST4 | <0.001 | 0 | - | down | 100 | 10 | RASAL1; KCNMB3; IRF4; AOX1; ROR1; SAMD4A; DACT1; SLAMF1; SYNPO; PSEN2 |
| Relapse | DNMT3A | <0.001 | 0 | - | down | 750 | 23 | DBN1; MCM10; TUBB; HMGB3; SEPHS1; HRB; TIMELESS; ZNF675; PRPF4B; C11orf21; LGALS1; IGFBP7; S100A4; P2RX5; NPR3; DKFZp686O1327; PSTPIP2; FOXP1; AHR; PLCB1; FLJ13197; CCPG1; TSPAN32 |
| T-ALL | MBD3 | <0.001 | 0.01129 | + | up | 2500 | 1015 | CD74; HLA-DRA; HLA-DRB1; PAX5; CD19; BLNK; HLA-DPA1; C14orf139; HLA-DPB1; HLA-DMA; HLA-DRB5; CD79B; MEF2C; CD24; TCL1A; BTK; CD79A; SNX2; VPREB3; JUP; PLCG2; CIITA; NCF4; POU2AF1; TFEB; IGHM; CHD7; GALNAC4S-6ST; CTNNA1; RIPK2; TCF4; HLA-DQB1; MSRA; INSR; HLX; LAMC1; INPP5D; STX7; SLC27A3; EGLN1; LAPTM5; LAT2; HLA-DMB; BANK1; TSPAN13; STX3; HLA-DRB6; PFTK1; CD9; CD22; PTPRE; BACH2; HCP5; GNG7; AUTS2; PTK2; RHBDF2; LILRA6; LILRA2; TRIM38; TRAK1; HLA-B; TLR1; tcag7.1314; CD72; FLJ20674; MARCH3; HLA-F; PDLIM1; LRP10; KIAA0040; PIK3CG; STK32B; EVI5; LILRB1; CSDA; LARGE; NLRP1; SHOC2; 3.8-1; CYB5R1; C7orf23; PIK3CD; FADS3; LOC90925; RGL1; PDE4B; TRIO; FAM65A; LY86; TULP4; C17orf60; OFD1; HLA-DOA; ERO1LB; UNC119; VPREB1; CTGF; HIST2H2AA3; HLA-J; BTG2; FOXO1; GALNT14; PXDN; CIDEB; SCML2; SAV1; PRDX1; HLA-G; SCARF1; MBP; SPINT1; SLC35F2; GRB10; QRSL1; RASSF2; S100A13; CTBP2; DENND3; C10orf10; NEIL1; CYFIP1; CRIM1; MYO5C; ALDH3A2; GAB1; P4HA2; KIAA0495; LRRK1; MDM2; SLC35D2; VIPR1; ELK3; LILRB2; DUSP1; WFS1; KIAA0323; CPM; OGFRL1; ECHDC3; TST; BTN2A2; ZRANB1; LILRB3; TSC22D3; RAI14; ARSD; KMO; SSH3; BCL2L2; BCAS4; TGIF1; SCARB2; NPY; TCL6; CD58; MME; NPR1; LRIG1; KIF13A; SLC35E3; MYLK; IL1B; CELSR2; EHD3; FAM50B; IGHG1; CSF2RB; SCPEP1; CAST; TRMT12; HLA-DRB3; SPIB; MYLIP; TERF2; BCAR3; NCF1; PIP5K1B; RAPGEF3; PSMB9; DRAM; KLHL2; GH1; MANBA; TAP2; STK38; GLRX; GNAI1; CD180; SFMBT1; TPST1; IL6ST; GNA12; LDLRAP1; RHOB; DIP2C; IQCK; PPFIBP1; DRP2; C14orf106; DACT1; KANK2; P2RX1; IRF4; ARHGEF12; HHEX; S100A1; C14orf113; WASF1; HMHB1; C14orf132; ZNF274; STAG3; ZFP36; PHACTR1; TLR2; CELSR1; IRF7; LDOC1; DYRK3; HIST1H2BF; SYT11; COL5A1; HLA-DOB; LRMP; SCHIP1; CORO2B; GORASP1; CXorf21; NUAK2; DGCR6; SLC16A2; OR2A9P; HIST1H2BG; SYT1; PAOX; PARP1; PPP3CC; IRF6; C12orf49; ISG20; F13A1; ACOT9; MAP7D3; DGKD; KLF11; CYP2B7P1; OCA2; HLA-DQA1; IRS2; ALOX5; VRK2; PRKCE; PDK1; EWSR1; PCDH9; MALT1; RNF19B; SERPINI2; ZNF193; ABCB4; TLE1; FGD2; CYBB; PCSK6; ECM1; ACTR2; HLA-A; GSTM5; AKAP12; MGC5370; LMO2; NFKB1; PRO2268; UNC13B; PDE8B; CCR1; NCF2; COCH; PRDM2; BMPR2; STAT2; ELL3; RIN2; COBL; MTSS1; C9orf167; SGSH; C9orf45; SPPL2B; LTB4R; MAN1A1; EMP2; CD200; NCOA3; BMP2; RASL10A; CRMP1; ENTPD1; FGD1; P2RY14; ERG; ZSCAN16; HCK; CDC25C; SAMD4A; ST6GALNAC4; TGFBR2; TNS3; JAM2; HIST1H3D; CCDC81; RCAN1; NINJ1; KHDRBS3; SDC2; SSX4; NRGN; HLA-DRB4; GABBR1; PBX2; HLA-E; MPEG1; SLC43A1; AASS; CRAT; CLEC2D; SKIL; ODZ4; GPR132; EAF2; SERHL2; HIST1H2BE; FAM108B1; DDEF1; BASP1; CAP2; HIST1H2BN; MYRIP; IGSF3; DSP; OR1A1; MRC1; NOL4; IFIT3; KCTD7; BIN1; OLFML2A; LTBP2; FAM3C; MBNL2; TBXA2R; ZFP36L1; B2M; CSNK1G3; FOXP1; OR7A5; CEACAM21; HIST1H2BH; NIPSNAP3B; ENTPD3; SEMA5A; TMEM2; LTBR; MS4A1; CD86; CXCR6; TCF3; SEMA6D; RAG2; CD40; SLC7A7; TBC1D9; EPHB3; MMP11; IFIT2; ELOVL2; KCNS3; BACE1; ATP10B; PGCP; SLC11A1; HIST1H2AM; ID3; CSHL1; SLC15A2; NLRP2; GNAZ; HIST1H2AJ; GAS8; EFNB1; KLF2; ADAM19; HIST1H1C; HSPA2; HOMER2; EZR; IL8; SPRY4; IL13RA1; TP53I11; ASB13; VCL; PTPRG; IQSEC1; DDR1; SEMA3F; NEBL; C5orf4; ADCK2; HIST1H4H; FLJ20489; RHOQ; ITIH3; BLK; ULK2; FER1L3; ZNF467; SLC17A7; LDB2; CKMT2; B4GALT1; ANKRD55; REEP1; LOH3CR2A; IL3RA; KIAA0774; ECM2; KIAA0746; CYP2B6; ORAI3; CLOCK; C10orf56; KCNJ2; C11orf24; SRGAP2; FLJ10357; HIST1H2BI; TSSC1; FKSG49; MYO10; DPEP1; TXNIP; MUC5AC; CTSO; IRAK3; SH2D3C; USP10; EPHA7; GJC1; IGLJ3; MLLT4; ALDOC; MGP; MLPH; ITIH4; IFI30; C6orf145; RBM47; TGFB1I1; GIMAP4; BC37295_3; ZNF117; SIGLEC15; UTRN; EGFR; CLEC4E; AK5; FERMT1; ATP10D; SPTA1; NFATC4; OPHN1; STARD13; SLC2A1; FLT1; APBB2; SP4; IMPACT; TCL1B; PIK3IP1; KCNMB1; SEMA6A; KIF1B; AQP1; SUOX; IGKV1OR2-108; PTGS1; CD52; ERBB2; MS4A6A; HIST1H2BD; EVC; HEY2; SYNPO; LILRB4; DKK1; HAP1; RBMS3; C5; GLDC; LRRC17; SF3B3; NOLC1; FAM89B; TMEM70; NOL14; POLR2B; EXOSC10; CHERP; TNPO3; PRR4; C12orf41; TMEM134; ZNHIT1; USP47; POP4; C1orf77; C19orf10; PIH1D1; CHMP2A; SLC20A1; UPF1; ACACA; RRM1; YY1; TSN; YARS; UBE3A; PMS2L8; LUC7L2; UQCRFS1; CRKL; PANK2; RAB7A; BXDC5; GNG5; PSMC4; UCHL3; MAD2L1; AASDHPPT; PSMB7; CCT7; HSP90AA1; PI4KA; ITGB3BP; HNRNPA2B1; MED26; PSCD2; TARDBP; REST; RPL36; PPP1R7; NIF3L1; YLPM1; SFRS9; NCAPG2; FAM128B; NDUFA9; PIK3R4; DHPS; NFYB; POLR2I; RBBP4; COPG; TBC1D2B; ARS2; BMS1; ADRM1; H2AFV; HSPA9; CCT2; C19orf50; LOC130074; AMZ2; YY1AP1; ZXDC; TMEM194; CTCF; C19orf29; UQCRC1; FAM128A; RFC1; TXNL1; SCAMP1; CCDC90A; IMP3; ACLY; JOSD1; COX6A1; GTSE1; ALG8; DRG1; FBXO7; NDUFB7; SRP72; WDR6; COX4NB; CHCHD2; TCEA1; B3GAT3; BCL7B; PSMA5; CCDC72; UQCRQ; C19orf60; ATP6V1H; C14orf166; NF2; XPOT; KARS; HMGN2; DCTN2; NAB1; SNRPD1; POLR2F; MRPL20; MRPL11; TRMT5; PFN1; MTHFD1; HNRPA3P1; ATP5A1; OLA1; ZNF131; CYCS; SUMO2; MRPL4; GOLPH3; TCEB2; FASTKD1; PPP2CB; ICMT; PGD; PPME1; SUCLG1; MRPL23; ARPC1A; ZC3H4; BRD7; SNRPB; MORF4L2; MBTPS1; BAZ1B; DPM3; LOC645139; ANKMY2; MUM1; CCNB1; ELMO2; MRPS15; ASNSD1; AATF; PDCD5; LOC552889; ANP32E; C19orf56; RAD54L; ATP5H; C20orf149; MBD3; LIG1; TPI1; ACYP1; FLJ14154; TAF9; FHOD1; HSPE1; CFDP1; H2AFZ; ALDOA; NAT13; SHMT2; UQCRH; ARL1; HSPC152; EIF2AK1; STX10; HMGN4; RNF4; DAZAP1; COPS5; ATXN10; SSBP1; CASC3; RPN2; HNRNPR; SEC24C; UBP1; GMCL1; SUPT5H; YWHAQ; ZNF518A; UBE4A; RBM14; NAP1L4; KHSRP; SNRPG; LONP1; EIF4E2; MCM6; TBC1D25; GCSH; C1orf149; XRCC6; USP1; RPA1; MDH2; TUBA1B; TMEM106C; HNRPAB; TTC31; NSMAF; GMNN; USP39; DTYMK; PTOV1; PSMD14; YBX1; PMS2L1; NRD1; RNASEH2A; ATP5J2; ATP1A1; COX8A; COX17; GPBP1L1; LRRC40; ZNF146; PSMD1; PCMT1; NUP93; PPT1; PTCD3; HADH; DDX39; GMPS; C12orf29; SMC3; GLB1; METT11D1; TMEM160; BOLA1; RBBP8; ANAPC13; MED16; hCG_1776980; CDC34; TOMM70A; PATZ1; TRIM28; PPAT; FTO; TDG; TMEM208; RNMT; PGRMC1; PCID2; EXOSC4; TCP1; ARMC1; NDUFC2; TXNL4A; KATNB1; ARF4; RUFY1; DDX1; MTX1; SAFB2; TMEM5; NUBP2; THOC5; ATF4; POLRMT; SND1; RANBP5; DDX18; C1orf41; PRPF19; POLR3E; ZNF574; DGCR8; C11orf10; IHPK2; PSMD2; NDC80; TMPO; LARP1; LAGE3; UNC50; HAX1; UQCR; MAP2K2; NDUFS8; TADA3L; WDR18; THAP7; MGA; METAP1; PREB; SUMO1; WDR59; SNRPF; GSTP1; LOC730107; KHDRBS1; LSM3; CUL4A; BOLA2; NUP37; DGKZ; NXT1; STMN1; SHFM1; PSMD8; ATP5SL; FBXW2; FANCL; C9orf16; TERF2IP; CHCHD3; C16orf80; ILF2; MRPL3; LSM5; TUBA1C; PPP1CC; RSAD1; DPP3; TCEB1P; NIT2; MLF1IP; SAMM50; GARS; WDR82; PCNT; ACTL6A; MYOD1; MTERFD1; LSM1; SEC61A1; METTL5; RFXANK; SRPK1; DYNC1H1; ATP5G3; PIN1; C20orf24; TCEB1; ENTPD6; GAMT; CBX3; SAC3D1; NDUFS6; IKBKAP; UBA2; MRPL9; UNG; LASP1; PHF20; MEA1; TBCD; FARSA; MPHOSPH10; CALM2; TAOK3; TIMM13; MDH1; NOLA1; GORASP2; CCBL2; PANK4; CLEC3B; DBF4; GPR89B; UBL5; KEAP1; MSL2L1; RFXAP; GTPBP8; PAICS; NDUFA7; ARHGAP15; AOF2; EIF3M; GSS; TMOD1; OXSR1; FBL; CDC16; DGUOK; TTLL12; PSMC2; RTN4; POLR2H; METTL9; CUTA; COX6C; TAF2; RFC3; GCDH; GPR89A; TRIM33; EFTUD1; PSMB2; CHMP7; ADA; UBAP2; MRPL48; IDH2; PPIF; SSR2; SNRPD2; NDUFB6; HLTF; SAP130; STAU1; POM121L1; VDAC3; NCDN; LETM1; SPECC1L; FADD; MSH2; ARFGAP1; ANP32B; GNA15; MIF; ATP13A3; UBE2V2; KIAA0494; C16orf61; NDUFA13; RPIA; PRR14; CLNS1A; FDXR; DNAJB6; RTN3; KIAA0241; NDUFA3; C19orf53; ZC3H13; GADD45GIP1; SEC61G; TFG; C11orf48; SPAST; UFD1L; FBXO46; NCOR2; EXOSC8; HINT1; ASF1A; SLC3A2; ATF2; ZDHHC4; LEPROTL1; ADH5; SLC25A38; PCM1; HMG2L1; MRP63; ST13; HYOU1; ATIC; UBR4; GMDS; FAF1; RHOG; CNOT7; C1orf174; C11orf51; PMF1; DRAP1; COX11; KIAA0152; SIVA1; INTS9; RAP1GDS1; SLC7A1; LDHB; ANKRD27; PRMT7; GRSF1; LCMT1; DNAJC9; PAPSS1; RAD21; FADS2; NUP50; ATP6V1B2; TFPT; GNAQ; ETFB; LOC150759; SFI1; FDPS; LSM4; HN1; FDFT1; TM2D3; PAFAH1B3; CLPP; FBXL12; PTP4A2; SLC1A4; CD47; LANCL1; SEMA4D; MAPKAPK3; CDK5RAP3; C9orf78; PELO; PEX5 |
| Normal | BAZ2A | 0.001 | 0 | + | up | 1500 | 79 | LOC552891; HPRT1; PRDX3; GOT1; NDUFA1; NDUFS5; ACAT1; APIP; HSDL2; PDHX; NDUFB2; COPS3; ADFP; COX7B; LAGE3; AIF1; SMS; RBBP7; UCHL5IP; PSMC5; UBE2A; ATP5J; HMGN1; ARMCX1; SOD1; ARPC5; BCAP31; RCOR1; HMGN3; PECI; HN1; SLC25A5; NDUFB8; EEF1E1; CCDC56; EBP; CD93; PSMG1; C4orf27; RWDD1; PGAM1; IRAK3; SLC9A6; PHB; FAM45B; AMPD3; NDUFA8; GALNT2; NFIL3; MYLIP; BFAR; CBLB; DLGAP4; HIP1; PTGDR; CD84; APOBEC3G; CDH11; ANGPT2; GRAMD1B; MICAL3; RAG2; MTMR3; LRRC8B; PCDH9; PCBP4; GLDC; AP1B1; RHBDL2; CLTC; CAPN3; LGR5; MPPED2; LHFPL2; CHST7; CHST2; STAP1; PTPRM; CENTG2 |
| MLL | BAZ2B | <0.001 | 0 | + | up | 750 | 131 | MME; CD52; MYH10; COL5A1; MPP1; DPEP1; AKAP12; PARD3; ITPR3; RAI14; MAGED1; SYNE2; HPS4; MYLK; FAM65A; DBN1; MDK; PXDN; IL1B; EGLN1; TCF4; HCP5; DDR1; KHDRBS3; ITIH4; EMP2; FGD1; BMPR2; FLOT1; EHD1; LRIG1; ARID5B; C14orf132; SLAMF7; DIP2C; SRRD; LDOC1; COBL; ZNF667; ODZ4; HLA-C; H2AFY2; RAPGEF3; PSMB9; KANK2; C10orf10; LRRN2; HLA-F; CORO2B; NRN1; HLA-A; IGFBP4; TJP2; PCBP4; GAS1; APOL6; ATRNL1; ITIH3; DOCK9; TIAM1; GOLGA3; GNA11; GCHFR; DEF8; HLA-J; HRK; PARD6A; TP53I11; LRP6; DRP2; RIN2; IGLL1; GTF2IRD1; SMTN; E2F1; RASIP1; KLF11; PLAC4; FADS3; IGKV1D-13; IGKV1OR15-118; 3.8-1; KIAA0562; STX12; STAM2; CDK7; AKAP11; TSPAN31; VPS24; MEMO1; EIF3D; MFSD1; TM9SF2; BZW2; VAMP3; IQGAP2; NSMAF; CARS2; ASAH1; VPS13D; ATRN; GUCY1A3; NFATC3; DNAJB6; RYBP; GSTK1; RASSF1; CEP70; UBA3; ARL8B; COPS7A; SRPRB; UPF3A; EMR2; MPG; IKBKB; ACAA1; JMJD1C; RASA1; MED13L; SMARCA2; RRAS2; TBC1D2B; SH3YL1; ARL6IP5; GALC; LY75; C1orf164; FEZ2; PCDHGC3; PLXNC1 |
| T-ALL | BAZ2B | <0.001 | 0 | + | up | 2000 | 561 | CD74; HLA-DRA; HLA-DRB1; PAX5; CD19; BLNK; HLA-DPA1; C14orf139; HLA-DPB1; HLA-DMA; HLA-DRB5; CD79B; CD24; TCL1A; BTK; VPREB3; JUP; POU2AF1; TFEB; IGHM; CHD7; GALNAC4S-6ST; CTNNA1; TCF4; HLA-DQB1; EGLN1; PTPN18; STX3; HLA-DRB6; PFTK1; CD9; CD22; BACH2; HCP5; GNG7; AUTS2; RHBDF2; TRIM38; HLA-B; tcag7.1314; FLJ20674; HLA-F; PDLIM1; KIAA0040; PIK3CG; LARGE; 3.8-1; C7orf23; PIK3CD; FADS3; LOC90925; RGL1; PDE4B; FAM65A; LY86; TULP4; OFD1; KCTD15; ERO1LB; VPREB1; HLA-J; BTG2; FOXO1; GALNT14; PXDN; NUDT3; SCML2; SAV1; PRDX1; HLA-G; SCARF1; SPINT1; QRSL1; RASSF2; S100A13; GTF2IRD1; C10orf10; NEIL1; CYFIP1; CRIM1; MYO5C; ALDH3A2; P4HA2; MDM2; VIPR1; ELK3; WFS1; CPM; ECHDC3; TST; BTN2A2; ZRANB1; LILRB3; RAI14; KMO; GNA11; TCL6; CD58; MME; LRIG1; KIF13A; SLC35E3; MYLK; IL1B; CELSR2; IGHG1; CSF2RB; BCAR3; NCF1; PIP5K1B; RAPGEF3; PSMB9; KLHL2; AK1; TAP2; GNAI1; CD180; SFMBT1; TPST1; LDLRAP1; RHOB; DIP2C; IQCK; PPFIBP1; DRP2; RBM38; C14orf106; DACT1; KANK2; IRF4; ARHGEF12; S100A1; C14orf113; WASF1; C14orf132; ZNF274; PHACTR1; CELSR1; LDOC1; DYRK3; SYT11; COL5A1; LRMP; CORO2B; PRKCD; GORASP1; SLC16A2; OR2A9P; SYT1; PAOX; PARP1; IRF6; C12orf49; F13A1; DGKD; KLF11; CYP2B7P1; TCIRG1; ALOX5; PDK1; EWSR1; MALT1; ABCB4; TLE1; FGD2; CYBB; ADRBK1; C3orf37; SMAD3; PCSK6; ECM1; HLA-A; GSTM5; AKAP12; NAV1; MGC5370; NFKB1; SH2B2; PDE8B; NCF2; SRRD; COCH; BMPR2; ELL3; RIN2; COBL; C9orf167; SGSH; SPPL2B; EMP2; CBR1; RASL10A; CRMP1; ENTPD1; FGD1; ZNF672; ZSCAN16; HCK; CDC25C; SAMD4A; ST6GALNAC4; TGFBR2; JAM2; CCDC81; RCAN1; NINJ1; TBC1D1; KHDRBS3; SSX4; NRGN; HLA-DRB4; GABBR1; AASS; CRAT; ODZ4; SMTN; EAF2; SERHL2; DDEF1; BASP1; HIST1H2BN; MXD3; IGSF3; DSP; OR1A1; NOL4; IFIT3; BIN1; HPS4; LTBP2; FAM3C; ZFP36L1; OR7A5; CEACAM21; ENTPD3; SEMA5A; MS4A1; CD86; CXCR6; TCF3; SEMA6D; RAG2; CD40; MICALL1; APBB1IP; EPHB3; IFIT2; ELOVL2; KCNS3; BACE1; ATP10B; SLC11A1; HIST1H2AM; ID3; NLRP2; GNAZ; GAS8; EFNB1; KLF2; ADAM19; HIST1H1C; HSPA2; EZR; TAP1; IL8; SPRY4; TREX1; TP53I11; IQSEC1; DDR1; SEMA3F; NEBL; C5orf4; ADCK2; FLJ20489; ITIH3; BLK; M-RIP; NARFL; ZNF467; SLC17A7; B4GALT1; ANKRD55; REEP1; LOH3CR2A; KIAA0774; ECM2; KIAA0746; CYP2B6; CLOCK; C10orf56; KCNJ2; SRGAP2; CENTA1; TSSC1; FKSG49; DPEP1; MUC5AC; SH2D3C; USP10; GJC1; MLLT4; MGP; MLPH; ITIH4; IFI30; BC37295_3; DNTT; SIGLEC15; EGFR; FERMT1; NFATC4; OPHN1; APBB2; SP4; TCL1B; KCNMB1; PSMB8; IGKV1OR2-108; CD52; ERBB2; MS4A6A; EVC; SYNPO; ALG6; MED23; OBFC1; NSUN5C; HSPBAP1; COX7A2L; MBTPS1; ZC3H11A; SC5DL; ANKMY2; MUM1; SECISBP2; ELMO2; ASNSD1; LOC552889; C19orf56; TAF9; FHOD1; ARL1; COPS5; CRBN; RPN2; FLJ11286; UBP1; ZNF518A; SS18; KIAA1797; UBE4A; RBM7; C1orf149; DZIP3; PRKACB; TALDO1; LRBA; TTC31; NSMAF; SERTAD2; CEPT1; PMS2L1; C2orf25; HSD17B7; NSUN5B; GPBP1L1; LRRC40; CASD1; IDH1; ZNF146; AMY1A; PPT1; NMI; PTCD3; HADH; C12orf29; GLB1; METT11D1; ANAPC13; MED16; TOMM70A; PATZ1; LOC220594; FTO; C20orf19; SACM1L; TDG; RNMT; PCID2; EDEM3; GTF2F2; ARF4; STK38L; TES; LOC728411; SAFB2; TMEM5; IQWD1; POLRMT; SND1; ZNF83; SLC25A44; DDX18; POLR3E; ZNF574; IHPK2; UNC50; BCKDHB; UQCR; SLC33A1; TADA3L; KIAA0999; TMED5; MGA; OSBPL2; PTPRC; WDR59; FAM48A; CUL4A; LIPC; ATP5SL; FBXW2; BIRC2; CASP4; RSAD1; TCEB1P; CTR9; C1orf103; HIBCH; THSD1; MYOD1; CEP63; FAM62A; SAMSN1; GARNL4; RFXANK; CD46; INPP4A; IKBKAP; SLC25A46; P2RX4; DMXL2; LASP1; PHF20; TAPBPL; MPHOSPH10; TAOK3; ERCC5; OSBP; SCP2; EPM2AIP1; GORASP2; CCBL2; CSGlcA-T; INSIG2; GPR89B; VPS8; SLC11A2; MSL2L1; ICAM2; TANK; REXO2; ARHGAP15; PTTG1IP; EIF3M; TMOD1; OXSR1; MDM1; CDC16; IFRD1; DGUOK; RTN4; FNDC3A; PIP4K2A; FNBP1; PFKP; C19orf2; TAF2; GCDH; GPR89A; TRIM33; DOCK2; CHMP7; SSR2; STAU1; VDAC3; PIK3CB; NCDN; FADD; SLC2A3P1; GNA15; KIAA0494; CEP70; PRR14; ITGAE; CLNS1A; DNAJB6; TSC22D1; KIAA0241; ZC3H13; TFG; ZBTB20; SPAST; EXOSC8; ATF2; POLI; IL2RG; DPYD; ZDHHC4; TLE3; CTSW; C12orf11; LEPROTL1; PCM1; ST13; CASP8; UBR4; RHOG; SELL; TMEM135; C1orf174; TRAF3IP3; DFFB; TBXAS1; ANKRD27; SIGIRR; GOLGA8G; GRSF1; TFRC; STAM; ITPR2; SSFA2; ABCC1; PAPSS1; ATP6V1B2; OSBPL3; STAT3; GNAQ; LOC150759; SLC25A20; ATP2C1; FDFT1; TM2D3; DHRS7; ARHGEF3; FBXL12; CD47; LANCL1; IMPA1; SEMA4D; MAPKAPK3; CDK5RAP3; NOTCH1; AGL; STAT5A; CMAH; C14orf135; LZTFL1; NUCB2; TOX |
| Relapse | BAZ2B | <0.001 | 0 | + | up | 750 | 24 | DBN1; HMG4L; NCAPH; TOP2A; MKI67; KIF4A; TPX2; COL1A1; CDCA3; MAGED1; FANCE; ERCC6L; HJURP; MYBL2; KIF11; CENPE; MYH10; AURKB; AKR1B1; EXO1; MFSD1; IDH1; CEP70; LEPROT |
| CCR | DNMT3A | <0.001 | 0 | + | up | 400 | 16 | S100A4; IGFBP7; MAP3K5; SECTM1; NLRP3; PTGES3; SFXN3; CHMP1B; SMEK1; POLE; ZNF273; MCM10; ZNF43; PRPF4B; ZNF675; DBN1 |
| MLL | DNMT3B | <0.001 | 0.06117 | + | up | 400 | 51 | MME; CD52; MYO5C; FHIT; COL5A1; DNTT; VAMP5; ALOX5; ELK3; SCHIP1; PXDN; STK32B; EGLN1; DUSP26; TCF4; HCP5; GIMAP4; KHDRBS3; SH3TC1; SLC35E3; FGD1; BMPR2; NAV1; tcag7.1314; ZNF467; PTP4A3; DIP2C; MGC5370; BTN2A2; PXN; RGL1; LDOC1; TREML2; LTB; RAPGEF3; KANK2; C10orf10; PDE4B; IGFBP4; SEMA6A; XYLT1; PTER; CEP70; KIAA0125; UBA3; BRPF1; PIGB; TBC1D2B; TPP2; HK2; PLXNC1 |
| MLL | HDAC1 | 0.006 | 0 | + | up | 2000 | 407 | MME; CD52; MYO5C; EXDL2; SERPINB9; FHIT; COL5A1; DNTT; MPP1; DPEP1; AKAP12; RAI14; SMAD1; ZNF274; ZNF135; GFOD1; ALOX5; ITGA5; ELK3; POLE; GRAMD1B; SCHIP1; MYLK; FAM65A; MDK; PXDN; IL1B; STK32B; EGLN1; DUSP26; TCF4; ID3; HCP5; GIMAP4; DAB2; DDR1; KHDRBS3; SH3TC1; ITIH4; SLC35E3; MYO1B; EMP2; FGD1; ABCG2; BMPR2; NAV1; tcag7.1314; ZNF467; PTP4A3; ZNF257; ASB13; LRIG1; LIG4; ARID5B; C14orf132; SUOX; CASK; SLAMF7; SETBP1; STAT4; DIP2C; MGC5370; EFNA1; BTN2A2; PXN; RGL1; SRRD; PRO2268; LDOC1; PLCH1; COBL; ZNF667; ODZ4; IGHA1; HLA-C; TREML2; H2AFY2; APP; KIAA0427; DYRK3; ERG; RAPGEF3; KANK2; C10orf10; ABCG1; LRRN2; HLA-F; CCND2; ATN1; POU4F1; PDE4B; SPANXA1; FAM127A; TBL1X; UTRN; CORO2B; NRN1; HLA-A; IGFBP4; PECAM1; ZNF117; ITGA6; DAPK1; FAM129A; SEMA6A; SPTA1; IGHG3; PCBP4; SERPINB6; GAS1; RASL10A; A2M; APOL6; ATRNL1; ITIH3; GRIK5; TNFRSF21; CRIM1; CYTL1; ASB9; GNA11; IFIT2; NOS2A; HERC5; GPHN; MED9; SCARF1; OPTN; EPHA7; TMEM140; LARGE; ARHGEF17; PTGDR; ST3GAL6; KIAA1166; HLA-J; WFS1; OAS3; SH3BP5; LGALS3BP; HRK; ECM1; FLJ20489; MRC1; FAM127B; GBP1; MCTP2; P2RY14; IFIH1; PDXK; TMEM156; OSBPL10; SOD2; TP53I11; LRP6; CEBPE; F2RL1; DRP2; NEO1; RIN2; ARHGAP24; DLGAP2; EFNB1; PIP3-E; NOD1; GTF2IRD1; SMTN; CD200; CTNS; DLG3; TSPAN5; RASIP1; NUDT11; KLF11; TBC1D9; PLAC4; FRMD4B; FADS3; IGKV1D-13; LOC57228; MDM2; IGKV1OR15-118; 3.8-1; NPDC1; SEMA3F; SLC22A5; ZSCAN16; GABBR1; NR3C2; MUC5AC; NEIL1; NINJ1; KIAA0774; MLXIP; BMPR1B; C16orf67; TIAF1; C14orf45; PRKCZ; GAB1; KIAA1466; PTPRM; VPREB3; KCNK3; ZNF253; HLA-G; PDK3; ELOVL2; NEDD9; NCF2; SAV1; SIGLEC15; FERMT1; TCL1A; ARHGEF4; MYT1L; TXNRD3; NTN2L; IFI6; PTPRK; MARCKS; CEACAM6; DDEF1; RAG1; SLC25A46; PPCS; VPS13B; GOLGB1; EIF2B4; FLJ11506; DAP3; C1orf149; ATP5F1; BRD9; B3GAT3; NFU1; EIF4H; SEC11A; TSC22D2; ANXA6; TAF9; ALDOA; PIGT; U2AF2; BUD31; BTBD1; ETFA; KIAA0999; AURKAIP1; ZNF313; C19orf50; BRP44L; TGOLN2; CHCHD3; NDUFA10; SRP19; PRPF38B; MTMR6; TCERG1; PDCD6IP; EIF3K; ATR; STAMBP; FXR1; CHORDC1; CDV3; GRINL1A; GNL2; NOL12; C2orf24; BID; SPG11; JARID2; ALG8; LYPLA1; RNASEH1; LEPROTL1; CHMP1A; SCP2; PLEKHB2; NDUFS3; FAM32A; PRPF40A; UBE4A; PHB2; CAPZA1; C9orf16; C22orf28; UFC1; DDX47; RTCD1; IDH3A; SAP18; LRRC47; HN1L; CIB1; NDUFB5; ATP5G2; MRPS30; R3HDM2; CHD4; UQCRC2; MAPRE1; MRPS34; ATP5B; DUSP12; TRAF3IP3; TMED5; CYB5R3; DNAJA3; RER1; ZDHHC4; EIF3I; RBM22; ESD; XPOT; RPIA; COPZ1; MDH2; ZRF1; TXNDC14; NEUROD1; DPP3; MRPL3; EIF6; CLNS1A; NSUN5; RAB14; TCF25; ANAPC5; HSBP1; CMAS; SHMT2; PMS1; PRR13; UCHL3; PPID; EIF3M; MRPS12; FDX1; DNAJA2; TSPAN3; COX5A; WBSCR22; TUFM; BXDC5; SPCS1; ARPC2; CEP63; EFHA1; HADH; MASP1; IMMT; ANKMY2; PPP1CA; AKR7A2; EIF2B1; RMND5A; SLC25A3; GABARAPL2; STRAP; UNC119B; CDK7; DIAPH1; PTDSS1; SYF2; IQGAP1; HMOX2; MEMO1; C15orf15; C20orf43; TWF1; ETF1; EIF3D; EXOC3; POP5; TM9SF2; PWP1; DCTN3; ATP5SL; NSMAF; LPCAT1; PELI1; SCYE1; ITGAE; PCGF1; CS; DNAJB6; ATP6V1F; UBA3; ARL8B; SRPRB; APEH; UPF3A; LOC440354; CLASP2; IKBKB; CYC1; ACAA1; PIGB; PABPC4; PTPRC; MAP4; TPP2; ADAM10; ARL6IP5; MBNL1 |
| T-ALL | HDAC1 | <0.001 | 0.04189 | + | up | 1000 | 281 | CD74; HLA-DRA; HLA-DRB1; PAX5; CD19; BLNK; HLA-DPA1; C14orf139; HLA-DPB1; HLA-DMA; HLA-DRB5; CD79B; CD24; TCL1A; BTK; VPREB3; JUP; POU2AF1; CHD7; TCF4; HLA-DQB1; LAMC1; SLC27A3; EGLN1; PTPN18; HLA-DMB; HLA-DRB6; CD9; PTPRE; HCP5; ENG; HLA-B; TLR1; tcag7.1314; HLA-F; PDLIM1; STK32B; LARGE; TSPAN14; 3.8-1; FADS3; RGL1; ADCY6; PDE4B; C17orf60; HLA-DOA; ERO1LB; HIST2H2AA3; HLA-J; BTG2; PXDN; FAM49A; CIDEB; SCML2; C1orf38; HLA-G; SCARF1; S100A13; C10orf10; CRIM1; MYO5C; ALDH3A2; GAB1; MDM2; SLC35D2; VIPR1; ELK3; WFS1; OGFRL1; ECHDC3; BTN2A2; ZRANB1; LILRB3; RAI14; ARSD; SSH3; GNA11; NPY; MME; NPR1; LRIG1; KIF13A; SLC35E3; MYLK; IL1B; HLA-DRB3; MYLIP; TERF2; RAB13; RAG1; RAPGEF3; DRAM; TAP2; TPST1; PPFIBP1; DRP2; KANK2; S100A1; C14orf113; C14orf132; TLR2; EPHB4; CELSR1; IRF7; LDOC1; DYRK3; HIST1H2BF; COL5A1; SCHIP1; OR2A9P; HIST1H2BG; IRF6; CYP2B7P1; OCA2; HLA-DQA1; ALOX5; LIMD1; EGFL7; ZNF193; ABCB4; FGD2; CYBB; ADRBK1; PCSK6; CRTAP; ECM1; GSTM5; NAV1; PRO2268; PDE8B; CCR1; NCF2; BMPR2; STAT2; RIN2; COBL; C9orf167; IFI35; LTB4R; EMP2; BMP2; RASL10A; ENTPD1; FGD1; P2RY14; ERG; CDC25C; HIST1H3D; SSX4; NRGN; GABBR1; PBX2; CRAT; ODZ4; GPR132; SERHL2; DDEF1; HIST1H2BN; IGSF3; DSP; OR1A1; MRC1; NOL4; IFIT3; OLFML2A; OR7A5; ENTPD3; SEMA5A; CD86; CXCR6; SEMA6D; CD40; TBC1D9; EPHB3; IFIT2; DLG1; CBX3; NDUFS6; IKBKAP; SLC25A46; UBA2; MRPL9; C13orf23; PHF20; SUB1; BZW1; CALM2; MRPL16; HDAC1; ERCC5; OSBP; SCP2; EPM2AIP1; MDH1; DBF4; GPR89B; UBL5; VPS8; MSL2L1; DECR1; NDUFA6; NDUFA7; ARHGAP15; AOF2; COX7C; EIF3M; MDM1; CDC16; DGUOK; PSMC2; RTN4; POLR2H; TROVE2; COX6C; TAF2; GPR89A; TRIM33; EFTUD1; PSMB2; DOCK2; MRPL48; SSR2; SNRPD2; STAU1; MRPL13; SLC2A3P1; MIF; UBE2V2; KIAA0494; C16orf61; NDUFA13; RPIA; ITGAE; CLNS1A; DNAJB6; NDUFA3; C19orf53; SEC61G; TFG; C11orf48; SPAST; UFD1L; EXOSC8; MTMR6; ATF2; ZDHHC4; LEPROTL1; SLC25A38; PCM1; MRP63; ATIC; UBR4; FAF1; BRP44L; DRAP1; KIAA0152; INTS9; SEC61B; LDHB; ABCD3; DCUN1D1; GRSF1; LCMT1; RAD21; NUP50; ATP6V1B2; TFPT; SFI1; FDPS; THYN1; LSM4; FBXL12; PTP4A2; CD47; LANCL1; IMPA1; INSIG1; SEMA4D; CDK5RAP3; C9orf78; NUCB2 |
| MLL | HDAC5 | 0.004 | 0 | + | up | 2500 | 592 | MGC29506; CD2AP; EXDL2; SV2A; SERPINB9; MYH10; PRKCH; PARD3; ITPR3; TNFRSF14; ST3GAL5; ZNF135; HYI; TRAF5; SYNE2; NEDD4; C11orf75; ARMCX2; ZNF329; GIMAP4; DAB2; MYO1B; YES1; RHOF; BLVRA; RYK; NDFIP1; ZNF257; ASB13; LIG4; CASK; SLAMF7; STAT4; FAM117A; PTPLA; ZNF512B; ZNF415; SALL2; TSPAN7; PLCH1; GPR56; ZNF667; IGHA1; HLA-C; TREML2; SPAG16; SLC5A3; XPA; ABLIM1; FBXO21; RAB15; ZNF529; LTB; GIMAP6; CCDC92; RAPGEF3; EPAS1; ABCG1; DHCR7; NOTCH2; NET1; RAB11A; FGFR1; PLCG1; CD27; TJP2; PECAM1; C6orf32; ITGA6; DAPK1; GIMAP5; FAM129A; SEMA6A; LST1; PHF17; IGHG3; ELOVL5; DSG2; ETV5; GAS1; A2M; APOL6; CTDSPL; RICS; ZBTB20; DOCK9; C2orf3; GRIK5; CD247; IFITM2; UBE2E3; ASB9; IFITM1; BEX4; IFIT2; GPHN; STAT5B; PLXND1; GCHFR; RAB27A; GTDC1; PRKCA; MRPS6; ARHGEF17; DEF8; FZD6; PTGDR; ST3GAL6; KIAA1166; LRP4; NF1; ENOSF1; OAS3; SH3BP5; HRK; NUDT6; PRDX4; ICAM3; GBP1; MCTP2; PVRIG; MFHAS1; ARHGAP29; ST8SIA4; B4GALT6; TMEM156; DLG5; OSBPL10; EHD2; F2RL1; C2CD2; MAST4; DRP2; ARHGAP24; DLGAP2; NOD1; HDGFRP3; GLS; CD8A; BCL11B; FAM102A; EDG1; TSPAN5; PIK3R3; NUDT11; CASP10; FUT8; CNN3; ACP6; PLAC4; IGKV1D-13; IGKV1OR15-118; NPDC1; LAX1; RBM9; MYBL1; SLC22A5; PTPRF; IL23A; ENPP4; SFXN1; FYB; MUC5AC; TTC9; TMEFF1; FAT; BMPR1B; KIAA1128; CSTB; FADS1; C16orf67; PTK7; C14orf45; ARNTL2; SEPP1; PSRC1; SEPT6; KIAA1466; PTPRM; CLIP4; KCNK3; ZNF253; C7orf44; RUNX3; PDK3; AJAP1; DGKA; LCK; SGPP1; KLHL3; FERMT1; PAWR; TXNRD3; NTN2L; FLJ14213; IFI6; TCF7; PTPRK; CENPE; CEACAM6; ASS1; EPOR; SCAND1; C17orf48; BECN1; GGNBP2; STXBP3; DCXR; MAPK9; BCR; VPS16; GTF2F1; ARSD; MOAP1; EHBP1L1; LAMP1; DNM1L; UBE2D2; CDIPT; DNAJC4; GNB2; NTAN1; MAFF; CCDC28A; MAP1LC3B; FPGS; NCOR1; PDXDC1; RNF19B; SCAMP2; NPTN; ATP6V0C; ARHGAP25; PWP2; RBM23; LRMP; ZDHHC7; SH3BGRL; TAX1BP3; SFRS17A; SCAMP3; TTLL3; BCL11A; VARS; SNX26; ADAM17; LAT2; ASL; HEATR1; SPSB3; KIN; SLC35F2; PCBP1; TLN1; AKT1; HEBP1; EIF1B; ARHGEF18; SWAP70; HMGA1; C16orf58; CPSF3L; PPCS; TBC1D16; AKAP1; EIF2B4; SKIL; CAPN1; JMJD2C; EXOC1; TLR2; PNPLA6; ABCF2; NEU1; MED15; MYO9B; TBC1D1; MSH5; ATP6V0B; IMPDH1; INSR; PPP3CB; EIF3CL; LRRFIP1; PLEKHM1; PPP3CA; NCOA3; PIGT; ULK1; HMG20B; U2AF2; C14orf94; ARHGEF6; AURKAIP1; RP2; KCTD15; ZNF783; FNTA; NDUFA10; MAPKAP1; GDI2; TXNDC13; DRG2; GLG1; RASGRP2; PCYOX1L; PDCD2; TSR1; PRPF38B; ERCC2; TCERG1; GPS1; ELOVL1; KRI1; FAM118A; SCAMP4; ARD1A; LOC728844; SPAG9; GNL2; ADD1; NR3C1; SPG11; GCN5L2; C5orf15; USP25; COMT; DDX28; RNF7; TRIP4; ZFP36; SLC12A9; CHMP1A; MAN2A2; ZMYND8; RPS6KA4; CTAGE5; CAPNS1; HCK; STX7; TAF11; UBE2E1; PRPF40A; PRKCD; FLJ20323; C22orf28; SEC61A2; UFC1; NUBP1; EML3; ERP29; INPP5B; LRRC47; HECTD3; HMHB1; MTM1; PPP2R3C; ATP5G2; FRAT1; MSH6; CHD4; MKNK2; GPX1; SLC25A11; PAK1IP1; TARBP1; GGA2; EIF3B; PRKCSH; CDC123; CD81; CLTA; MICAL1; ASPSCR1; EIF4A1; ABI1; PLCG2; MMP17; LY86; CCDC22; SOLH; SETDB1; KDELR1; GGA1; KPNA3; CTSB; RAB4B; KIAA0100; IRAK1; MGC3032; NIPBL; TOMM20; RBM22; GTPBP4; PEX11B; CTSA; CYB5R1; MYCBP2; ATP5S; MEF2D; ARPC1B; ADI1; COPZ1; ZRF1; TNIP2; EIF6; C1orf108; RAB5B; TCF25; PPP2R5C; TRIM8; DYNLT1; CTGF; OS9; PRR13; PDHX; MAN2C1; SDF4; NCBP2; GIT2; GTPBP6; PTPLB; BLK; RNF40; QTRTD1; CECR5; CBX4; NAGPA; CCNB1IP1; POLR3C; WDR19; ARPC3; BANK1; EZR; LMO2; HLX; MANBA; BASP1; VPS37B; NCK2; SEH1L; IFNGR1; ARHGAP1; SYNGR2; EAF2; PPP1CA; EIF2B1; PPFIA1; RMND5A; CDKN1A; DBI; UNC119; SMPD2; GABARAPL2; GLA; HEXB; UTP6; DERA; CDK7; DIAPH1; SLC25A6; CAP1; FAM53B; STAT6; SNX17; SYF2; ELAC2; WDR45L; PES1; MMP11; ARHGAP17; RABEP2; CASP9; MEMO1; SUPT4H1; CLIP1; ATG12; C20orf43; PTPN6; TMEM149; EFHC1; TRIM34; MEF2A; OTUB1; SPIB; NIP7; SMURF1; MFSD1; VAMP8; HBEGF; PSCD4; CHKB; HDAC9; TSPO; ARL2; ADPGK; NSF; CARS2; TPD52L2; NUP214; CSRP2; ARFGAP2; FES; DUSP22; VPS13D; DDX21; PYCARD; EI24; MFSD10; LRCH4; FLJ42627; ATP8A1; FRAT2; CS; QARS; RHBDF2; AHCY; BCL7A; M6PRBP1; EHD4; CYBA; DGKD; GLT25D1; RASSF1; MPV17; TBCC; AMPD2; ABR; TBC1D9B; BRPF1; RNFT1; DUSP3; DDEF2; DVL1; MARCH3; GDF11; MYH9; GMIP; SH3BGRL3; RPS6KA1; FLT3; CLASP2; IKBKB; MYO1F; PLXNB2; ACAA1; CYB561D2; JMJD1C; HCLS1; RASA1; PUS1; CLEC2D; VNN1; SMARCA2; ZNF394; ABAT; IGF2BP3; COQ2; MAP4; ASXL1; LOC100137047-PLA2G4B; RPP40; GLT8D1; BCAS4; SIRT7; IDUA; SH3YL1; C22orf9; PRKCE; KCTD5; KCNK12; MAN2B1; ZEB2; MAST3; C12orf5; AK2; CDKN1B; PTCH1; CD72; C1orf164; ADCY9; FEZ2; TUBB6; FAIM; GPM6B; SCPEP1; NLRP3; PCDHGC3; DAD1; PCDHGA1; MPZL1; ABHD4 |
| E2A-PBX1 | HDAC4 | <0.001 | 0 | + | up | 400 | 51 | SOCS2; ACSL5; CCND2; RNASET2; GALNT1; C1orf38; LTB4R; HPCAL1; SH2B3; XBP1; HHEX; EPHB4; EGFL7; SMC6; ITPR1; GPX1; SYNGR1; C13orf18; INTS3; FUCA1; CD97; UTRN; PTPN2; NUCKS1; HDAC4; MMD; CYFIP2; MAGEF1; PBK; ELOVL5; TOR3A; ITPKB; CSRP1; ARL4C; UCHL5; SS18L2; MAP1A; PTBP2; SCCPDH; SYNJ2; FH; TUSC4; RHOBTB1; TRIB2; ODC1; TMEM183A; D4S234E; ACAD8; PITPNC1; ADARB1; FAT |
| MLL | HDAC4 | <0.001 | 0 | + | up | 500 | 60 | MME; CD52; MYO5C; FHIT; COL5A1; DNTT; SMAD1; GFOD1; ALOX5; ITGA5; ELK3; SCHIP1; FAM65A; STK32B; EGLN1; DUSP26; TCF4; HCP5; GIMAP4; SLC35E3; MYO1B; FGD1; NAV1; tcag7.1314; ZNF467; ASB13; HIF1A; SUOX; MGC5370; EFNA1; RGL1; PRO2268; APP; ERG; RAPGEF3; HLA-F; CCND2; POU4F1; PDE4B; UTRN; HLA-A; ITGA6; SEMA6A; SPTA1; SERPINB6; RASL10A; CRIM1; CYTL1; GNA11; IFIT2; NOS2A; SCARF1; GRAMD4; ITGAE; DNAJB6; CDKN2D; UCK2; CAPG; SLC4A7; ADAM10 |
| T-ALL | HDAC7 | <0.001 | 0.0326 | + | up | 500 | 91 | PAX5; CD19; BLNK; BTK; IGHM; CD72; FLJ20674; MARCH3; EVI5; CYB5R1; LOC90925; LY86; TULP4; ADCY9; KCTD15; ERO1LB; VPREB1; GALNT14; SLC35F2; ZEB2; ALDH3A2; VIPR1; ECHDC3; TST; KMO; BCAS4; CD58; IL1B; EHD3; CSF2RB; SCPEP1; SPIB; NCF1; KLHL2; HMG2L1; CD2AP; CASP8; UBR4; CNOT6; CD300A; RHOG; SELL; ZNF512B; ENOSF1; TSPAN7; RARRES3; SEPT6; SIGIRR; STAM; LCMT1; CENTB1; RAD21; NF1; FGFR1; GNAQ; STAT5B; LOC150759; SFI1; GNPDA1; ICAM3; TCF20; PLCG1; EVL; CCDC92; LAX1; RHOH; TRGV9; SLC9A3R1; NDFIP1; TRGC2; TARP; IL27RA; SEMA4D; FAM117A; LCP2; RGS10; CDK5RAP3; SELPLG; NOTCH1; LIME1; STAT5A; CD6; SEPW1; TOX; TCF7; DENND2D; IL23A; BIN2; LCK; SCD; CD3D |
| T-ALL | SMARCC1 | 0.003 | 0.08862 | + | up | 2500 | 962 | CD74; HLA-DRA; HLA-DRB1; PAX5; CD19; BLNK; HLA-DPA1; C14orf139; HLA-DPB1; HLA-DMA; HLA-DRB5; CD79B; MEF2C; CD24; TCL1A; BTK; CD79A; VPREB3; JUP; PLCG2; CIITA; POU2AF1; TFEB; IGHM; CHD7; GALNAC4S-6ST; CTNNA1; RIPK2; TCF4; HLA-DQB1; INSR; HLX; LAMC1; STX7; SLC27A3; EGLN1; LAPTM5; PTPN18; LAT2; HLA-DMB; BANK1; TSPAN13; STX3; HLA-DRB6; PLXNB2; PFTK1; CD9; PTPRE; HCP5; GNG7; AUTS2; PTK2; RHBDF2; LILRA6; LILRA2; TRIM38; ENG; HLA-B; TLR1; tcag7.1314; CD72; FLJ20674; MARCH3; DYNLT1; HLA-F; FHL1; PDLIM1; LRP10; KIAA0040; STK32B; PHLPP; LILRB1; CSDA; SOCS2; LARGE; NLRP1; TSPAN14; 3.8-1; FADS3; RGL1; ADCY6; PDE4B; FAM65A; LY86; ADCY9; C17orf60; OFD1; KCTD15; HLA-DOA; ERO1LB; UNC119; VPREB1; CTGF; HIST2H2AA3; HLA-J; BTG2; GALNT14; PXDN; FAM49A; CIDEB; SCML2; C1orf38; SAV1; HLA-G; SCARF1; MBP; SPINT1; GRB10; RASSF2; S100A13; KIAA1033; CTBP2; PSD3; DENND3; CDKN1A; CDYL; C10orf10; NEIL1; RFTN1; CYFIP1; CRIM1; MYO5C; ALDH3A2; GAB1; P4HA2; KIAA0495; LRRK1; MDM2; SLC35D2; VIPR1; ELK3; LILRB2; WFS1; IRF8; CPM; OGFRL1; ECHDC3; BTN2A2; ZRANB1; LILRB3; TSC22D3; RAI14; ARSD; KMO; SSH3; BCL2L2; FES; GNA11; NPY; TCL6; MME; NPR1; LRIG1; KIF13A; SLC35E3; MYLK; IL1B; FAM50B; IGHG1; CAST; TRMT12; GSN; HLA-DRB3; SPIB; MYLIP; TERF2; BCAR3; RAB13; NCF1; PIP5K1B; RAPGEF3; DRAM; KLHL2; GH1; TAP2; GNAI1; CD180; SFMBT1; SLC2A5; TPST1; IL6ST; GNA12; LDLRAP1; DIP2C; PPFIBP1; DRP2; DACT1; KANK2; ARHGEF12; HHEX; S100A1; C14orf113; WASF1; HMHB1; C14orf132; ZNF274; STAG3; FUCA1; PHACTR1; TLR2; CELSR1; IRF7; LDOC1; DYRK3; HIST1H2BF; SYT11; COL5A1; RNASET2; HLA-DOB; LRMP; SCHIP1; CORO2B; CXorf21; NUAK2; DGCR6; SLC16A2; OR2A9P; HIST1H2BG; PAOX; PPP3CC; IRF6; C12orf49; ISG20; F13A1; ACOT9; KLF11; CYP2B7P1; TCIRG1; UNC93B1; OCA2; HLA-DQA1; BRF1; ALOX5; LIMD1; RNF19B; SERPINI2; EGFL7; ZNF193; ABCB4; TLE1; FGD2; CYBB; SMAD3; PCSK6; ECM1; HLA-A; GSTM5; NAV1; HIST1H2BK; MGC5370; LMO2; SH2B2; PRO2268; UNC13B; PDE8B; CCR1; NCF2; SRRD; PPM1F; BMPR2; STAT2; ELL3; RIN2; COBL; MTSS1; DUSP6; C9orf167; SGSH; SPPL2B; IFI35; LTB4R; MAN1A1; EMP2; CBR1; HIST2H2BE; BMP2; RASL10A; CRMP1; ENTPD1; GPM6B; FGD1; P2RY14; ERG; HCK; CDC25C; CTSB; SAMD4A; JAM2; HIST1H3D; CCDC81; RCAN1; KHDRBS3; TNS1; RALB; SSX4; NRGN; HLA-DRB4; GABBR1; LHFP; PBX2; HLA-E; MPEG1; AASS; DUSP3; CRAT; KCNA5; ODZ4; GPR132; EAF2; SERHL2; HIST1H2BE; DDEF1; CAP2; HIST1H2BN; MXD3; MYRIP; IGSF3; DSP; OR1A1; MRC1; NOL4; CYB5R2; IFIT3; KCTD7; BIN1; OLFML2A; LTBP2; FAM3C; FLT3; TBXA2R; FOXP1; OR7A5; CEACAM21; HIST1H2BH; NIPSNAP3B; ENTPD3; SEMA5A; TMEM2; LTBR; MS4A1; CD86; CXCR6; SEMA6D; CD40; MICALL1; SLC7A7; TBC1D9; EPHB3; IFIT2; ELOVL2; KCNS3; BACE1; ATP10B; SLC11A1; HIST1H2AM; ID3; CSHL1; SLC15A2; NLRP2; GNAZ; HIST1H2AJ; GAS8; EFNB1; KLF2; ADAM19; HIST1H1C; HSPA2; HOMER2; SPRY4; IL13RA1; ASB13; VCL; PTPRG; DDR1; SEMA3F; NEBL; C5orf4; ADCK2; HIST1H4H; FLJ20489; RHOQ; BLK; ULK2; FER1L3; SPTLC2; ZNF467; TRPM2; SLC17A7; LDB2; FLOT2; CKMT2; B4GALT1; ANKRD55; REEP1; ITGA5; LOH3CR2A; IL3RA; KIAA0774; ECM2; KIAA0746; CYP2B6; CLOCK; C10orf56; KCNJ2; C11orf24; CENTA1; HIST1H2BI; FKSG49; MYO10; DPEP1; MUC5AC; IRAK3; SH2D3C; INTS3; EPHA7; GJC1; MGP; MLPH; ITIH4; IFI30; C6orf145; RBM47; UGCG; TGFB1I1; GIMAP4; BC37295_3; DNTT; ZNF117; SIGLEC15; UTRN; TBC1D16; EGFR; CLEC4E; LOC643641; AK5; PLVAP; FERMT1; ATP10D; SPTA1; NFATC4; OPHN1; STARD13; SLC2A1; FLT1; SP4; IMPACT; TCL1B; PIK3IP1; TNFSF13; ALDH3B1; KCNMB1; SEMA6A; KIF1B; AQP1; SUOX; IGKV1OR2-108; PTGS1; CD52; CENTD1; ERBB2; MS4A6A; HIST1H2BD; EVC; HSPG2; SYNPO; DKK1; HAP1; CDS2; RBMS3; C5; LRRC17; SCN1B; MRPS34; PCNP; THOC7; SLC38A2; SMARCC1; SUPT6H; EIF4E; SF3B3; NOLC1; FAM89B; HDAC2; POLR2B; FUBP3; MAPBPIP; EXOSC10; CHERP; TNPO3; C12orf41; GLOD4; SNRPE; ZNHIT1; PARL; POP4; C1orf77; C19orf10; PIH1D1; CHMP2A; UPF1; ACACA; YY1; TSN; YARS; FAM60A; UBE3A; PMS2L8; LUC7L2; UQCRFS1; CRKL; GPI; RPL24; BXDC5; GNG5; PSMC4; UCHL3; AASDHPPT; RUNX1; QRICH1; TXNDC1; PSMB7; TPRKB; CCT7; PI4KA; ITGB3BP; HNRNPA2B1; PSCD2; TARDBP; REST; PPP1R7; NIF3L1; PAAF1; YLPM1; SFRS9; FAM128B; RNASEH2B; HYPK; MORF4; NDUFA9; DHPS; NFYB; TTC33; BTBD1; POLR2I; RBBP4; COPG; VPS13B; TBC1D2B; ARS2; BMS1; ADRM1; H2AFV; HSPA9; CCT2; C19orf50; LOC130074; ADSS; YY1AP1; MRPL22; ZXDC; CTCF; C15orf44; C19orf29; UQCRC1; TIMM17A; NPEPPS; TXNL1; SCAMP1; CCDC90A; IMP3; ACLY; BCKDHA; ZBTB11; COX6A1; ALG8; DRG1; FBXO7; PAIP1; NDUFB7; SRP72; WDR6; ISG20L2; DCTN6; SRP9; COX4NB; CHCHD2; C9orf82; BCL7B; PSMA5; CCDC72; UQCRQ; C14orf166; ERBB2IP; NF2; XPOT; KARS; EIF4G2; DCTN2; SEPT7; SNRPD1; POLR2F; MRPL20; MARCKSL1; MRPL11; TRMT5; PFN1; HNRPA3P1; ATP5A1; OLA1; TMED3; ATP5E; ZNF131; RIF1; CYCS; SUMO2; MRPL4; IVNS1ABP; GOLPH3; TCEB2; PGD; C3orf28; ALG6; SUCLG1; MRPL23; ARPC1A; ZC3H4; BRD7; SNRPB; COX7A2L; MBTPS1; BAZ1B; LOC645139; FLJ11506; SKP1; SECISBP2; PSMA4; MRPS15; ASNSD1; AATF; PDCD5; LOC552889; ANP32E; MBD3; LIG1; TPI1; FLJ14154; TAF9; HSPE1; CFDP1; ALDOA; R3HDM1; NAT13; SHMT2; UQCRH; HSPC152; EIF2AK1; STX10; HMGN4; RNF4; DAZAP1; COPS5; ATXN10; SSBP1; CASC3; HNRNPR; SEC24C; UBP1; GMCL1; SUPT5H; YWHAQ; UBE4A; RBM14; NAP1L4; COX6B1; KHSRP; SNRPG; LONP1; CDV3; FKSG30; HSPA8; GCSH; C1orf149; XRCC6; USP1; POLR1D; RPA1; MDH2; TUBA1B; HNRPAB; NSMAF; USP39; PTOV1; LDHA; PSMD14; YBX1; PMS2L1; C2orf25; RNASEH2A; ATP5J2; ATP1A1; LRRC40; NUP93; YWHAH; PPT1; NDUFB4; PTCD3; HADH; DDX39; NDUFA4; SMC3; METT11D1; NGRN; TMEM160; ALDH18A1; BID; hCG_1776980; CDC34; TOMM70A; TBL2; PATZ1; TRIM28; FTO; SACM1L; TDG; TMEM208; RNMT; PCID2; EDEM3; TCP1; GTF2F2; ARMC1; GSPT1; NDUFC2; KATNB1; ARF4; DDX1; MAGOHB; MTX1; SAFB2; NUBP2; THOC5; ATF4; POLRMT; SND1; RANBP5; SLC25A44; DDX18; ZMYM2; C1orf41; PRPF19; POLR3E; ZNF574; C11orf10; IHPK2; PSMD2; SNX4; EID1; TMPO; LARP1; HAX1; BCKDHB; MAP2K2; NDUFS8; WDR18; MGA; PREB; SUMO1; WDR59; SNRPF; LOC730107; KHDRBS1; LSM3; GOT2; CUL4A; MTHFD2; NUP37; NXT1; STMN1; SHFM1; PSMD8; PRKRA; ATP5SL; FBXW2; C9orf16; TERF2IP; CCDC86; CHCHD3; C16orf80; ILF2; MRPL3; LSM5; GATAD2A; TUBA1C; PPP1CC; RSAD1; DPP3; TCEB1P; NIT2; CTR9; SAMM50; GARS; WDR82; ARFGEF1; NIPSNAP1; ACTL6A; MYOD1; SERP1; METTL5; RFXANK; DYNC1H1; ATP5G3; PIN1; INPP4A; C20orf24; TCEB1; PRUNE; DLG1; CBX3; SAC3D1; IKBKAP; SLC25A46; UBA2; TTC27; MRPL9; UNG; DMXL2; C13orf23; CEP57; C12orf47; FARSA; BZW1; MPHOSPH10; CALM2; MRPL16; HDAC1; SCP2; TSPYL4; TIMM13; EPM2AIP1; AMD1; MDH1; NOLA1; GORASP2; CCBL2; PANK4; CLEC3B; DBF4; GPR89B; UBL5; KEAP1; POGK; MSL2L1; RFXAP; AZIN1; PAICS; NOL11; NDUFA6; NDUFA7; AOF2; COX7C; EIF3M; TMOD1; OXSR1; FBL; MDM1; DGUOK; PSMC2; POLR2H; METTL9; CUTA; COX6C; TAF2; RFC3; GCDH; GPR89A; TRIM33; EFTUD1; PSMB2; DOCK2; UBAP2; MRPL48; IDH2; PPIF; SSR2; SNRPD2; NDUFB6; HLTF; STAU1; VDAC3; SPECC1L; MRPL13; TRIM37; MSH2; SLC2A3P1; C5orf13; ANP32B; MIF; ATP13A3; THEM2; UBE2V2; KIAA0494; C16orf61; NDUFA13; RPIA; CLNS1A; DNAJB6; RTN3; KIAA0241; ZBED4; NDUFA3; C19orf53; ZC3H13; GADD45GIP1; SEC61G; TFG; SPAST; NCOR2; EXOSC8; ATF2; ATMIN; ZDHHC4; C12orf11; LEPROTL1; SLC25A38; PCM1; HMG2L1; MRP63; ST13; HYOU1; ATIC; PGM1; UBR4; GMDS; CNOT6; CNOT7; C1orf174; PMF1; MTMR2; DRAP1; KIAA0152; INTS9; SEC61B; LDHB; GRSF1; LCMT1; DNAJC9; RAD21; NUP50; ATP6V1B2; TFPT; LOC150759; FDPS; LSM4; PAFAH1B3; CLPP; FBXL12; PTP4A2; CD47; LANCL1; SEMA4D; HIRA; C9orf78 |
| T-ALL | SMARCC2 | 0.009 | 0 | + | up | 2500 | 980 | CD74; HLA-DRA; HLA-DRB1; PAX5; CD19; BLNK; HLA-DPA1; C14orf139; HLA-DPB1; HLA-DMA; HLA-DRB5; CD79B; MEF2C; CD24; TCL1A; BTK; CD79A; SNX2; VPREB3; JUP; PLCG2; CIITA; NCF4; POU2AF1; TFEB; IGHM; CHD7; GALNAC4S-6ST; CTNNA1; RIPK2; TCF4; HLA-DQB1; INSR; HLX; LAMC1; GGA2; STX7; SLC27A3; EGLN1; LAPTM5; PTPN18; LAT2; HLA-DMB; BANK1; TSPAN13; STX3; HLA-DRB6; PLXNB2; PFTK1; CD9; PTPRE; BACH2; HCP5; GNG7; PTK2; RHBDF2; LILRA6; LILRA2; TRIM38; TLR1; tcag7.1314; CD72; FLJ20674; MARCH3; DYNLT1; SIPA1; CSRP2; HLA-F; CDK9; FHL1; PDLIM1; KIAA0040; STK32B; LILRB1; LARGE; NLRP1; 3.8-1; FADS3; RGL1; ADCY6; PDE4B; GPD1L; LY86; ADCY9; C17orf60; OFD1; KCTD15; HLA-DOA; ERO1LB; VPREB1; CTGF; HIST2H2AA3; DOK3; HLA-J; BTG2; FOXO1; FAM49A; CIDEB; SCML2; C1orf38; PRDX1; SCARF1; MBP; SPINT1; GRB10; QRSL1; S100A13; ZMYND8; KIAA1033; PSD3; DENND3; HEXB; CDKN1A; CDYL; C10orf10; NEIL1; CYFIP1; CRIM1; ALDH3A2; GAB1; GPX1; P4HA2; KIAA0495; CYBA; LRRK1; MDM2; SYNGR1; SLC35D2; VIPR1; ELK3; WFS1; IRF8; KIAA0323; OGFRL1; ECHDC3; TST; BTN2A2; ZRANB1; LILRB3; RAI14; ARSD; ETS2; KMO; SSH3; BCAS4; TGIF1; FES; GNA11; NPY; TCL6; NPR1; KIF13A; SLC35E3; IL1B; FAM50B; IGHG1; SCPEP1; CAST; GSN; HLA-DRB3; SPIB; MYLIP; TERF2; BCAR3; RAB13; NCF1; ATP6V0D1; PIP5K1B; RAPGEF3; DRAM; KLHL2; PFKL; MANBA; AK1; TAP2; CD180; TPST1; LDLRAP1; IQCK; PPFIBP1; DRP2; C14orf106; DACT1; KANK2; ARHGEF12; HHEX; S100A1; C14orf113; CLCN7; WASF1; HMHB1; C14orf132; PHACTR1; TLR2; EPHB4; CELSR1; IRF7; LDOC1; DYRK3; HIST1H2BF; COL5A1; HLA-DOB; LRMP; SCHIP1; CXorf21; NUAK2; DGCR6; SLC16A2; OR2A9P; HIST1H2BG; SYT1; PAOX; IRF6; C12orf49; F13A1; ACOT9; DGKD; KLF11; CYP2B7P1; UNC93B1; OCA2; HLA-DQA1; BRF1; ALOX5; HBEGF; PRKCE; PDK1; LIMD1; PCDH9; SERPINI2; ZNF193; MFSD10; ABCB4; TLE1; FGD2; EHD4; CYBB; ADRBK1; SMAD3; PCSK6; CRTAP; ECM1; ACTR2; GSTM5; NAV1; HIST1H2BK; MGC5370; IGF2BP3; LMO2; SIDT2; SH2B2; PRO2268; UNC13B; PDE8B; CCR1; NCF2; SRRD; SFRS2IP; MAP3K5; PPM1F; BMPR2; STAT2; ELL3; RIN2; COBL; MTSS1; TAZ; KIAA1539; C9orf167; SGSH; C9orf45; SPPL2B; IFI35; LTB4R; EMP2; NCOA3; CBR1; HIST2H2BE; RASL10A; CRMP1; ENTPD1; GPM6B; FGD1; MAST3; P2RY14; HCK; CDC25C; SAMD4A; ST6GALNAC4; TNS3; JAM2; HIST1H3D; CCDC81; NINJ1; KHDRBS3; SDC2; TNS1; SSX4; NRGN; HLA-DRB4; GABBR1; LHFP; MPEG1; SLC43A1; DUSP3; CRAT; CLEC2D; SKIL; PEX16; ODZ4; SMTN; TRABD; GPR132; EAF2; SERHL2; HIST1H2BE; FAM108B1; BASP1; CAP2; HIST1H2BN; MXD3; SMURF1; MYRIP; IGSF3; DSP; OR1A1; MRC1; NOL4; CYB5R2; IFIT3; KCTD7; BIN1; OLFML2A; LTBP2; FAM3C; FLT3; MBNL2; FOXP1; OR7A5; HIST1H2BH; NIPSNAP3B; ENTPD3; SEMA5A; LTBR; CD86; CXCR6; TCF3; SEMA6D; CD40; SLC7A7; APBB1IP; TBC1D9; EPHB3; MMP11; DUSP22; IFIT2; ELOVL2; BACE1; ATP10B; PGCP; SLC11A1; RNFT1; GLT25D1; HIST1H2AM; ID3; CSHL1; SLC15A2; NLRP2; GNAZ; HIST1H2AJ; GAS8; EFNB1; ADAM19; HIST1H1C; HSPA2; HOMER2; EZR; IL8; SPRY4; IL13RA1; VCL; SEMA3F; NEBL; C5orf4; ADCK2; HIST1H4H; RHOQ; ITIH3; BLK; ULK2; FER1L3; SPTLC2; ZNF467; TRPM2; SLC17A7; CKMT2; ANKRD55; REEP1; LOH3CR2A; IL3RA; KIAA0774; ECM2; KIAA0746; CYP2B6; C10orf56; KCNJ2; C11orf24; SRGAP2; FLJ10357; CENTA1; HIST1H2BI; FKSG49; DPEP1; MUC5AC; APAF1; IRAK3; SH2D3C; USP10; EPHA7; GJC1; IGLJ3; MLLT4; MGP; MLPH; ITIH4; IFI30; C6orf145; RBM47; UGCG; TGFB1I1; BC37295_3; ZNF117; SIGLEC15; UTRN; TBC1D16; EGFR; CLEC4E; AK5; PLVAP; FERMT1; SPTA1; NFATC4; OPHN1; STARD13; SLC2A1; APBB2; SP4; TCL1B; TNFSF13; ALDH3B1; KCNMB1; SEMA6A; KIF1B; SAMHD1; AQP1; SUOX; IGKV1OR2-108; C1orf78; PTGS1; PCDHGC3; PCDHGA1; ERBB2; MS4A6A; EVC; HSPG2; SYNPO; LILRB4; DKK1; HAP1; TGIF2; RBMS3; C5; LRRC17; SCN1B; PCNP; THOC7; SLC38A2; SMARCC1; NDUFAF1; SUPT6H; RABGEF1; DYNC1I2; SF3B3; RCHY1; FAM89B; NCOR1; POLR2B; FUBP3; EXOSC10; CHERP; PRR4; C12orf41; TMEM134; USP47; PARL; POP4; C1orf77; SYNE2; PSIP1; SLC20A1; ACACA; YY1; TSN; YARS; FAM60A; UBE3A; LUC7L2; UQCRFS1; CRKL; PANK2; RPL24; RAB7A; BXDC5; KIAA0528; GNG5; R3HDM2; CYFIP2; PROSC; QRICH1; PSMB7; CCT7; HSP90AA1; PI4KA; HNRNPA2B1; PSCD2; SETD5; TARDBP; REST; RPL36; PPP1R7; C5orf3; YLPM1; SFRS9; COX7A2; FAM128B; MORF4; MAN1A2; NDUFA9; PIK3R4; DHPS; BTBD1; POLR2I; RBBP4; NCOA1; ARS2; BMS1; H2AFV; HSPA9; CCT2; LOC130074; ZFAND6; APPBP2; NOSIP; ADSS; YY1AP1; MRPL22; CTCF; C15orf44; UQCRC1; MUT; NPEPPS; TXNL1; SCAMP1; UGP2; ACLY; JOSD1; H3F3A; ZBTB11; COX6A1; ALG8; DRG1; FBXO7; PAIP1; CCDC93; SRP72; WDR6; ISG20L2; DCTN6; SRP9; CHCHD2; C9orf82; TCEA1; B3GAT3; BCL7B; PSMA5; CCDC72; UQCRQ; C19orf60; C14orf166; ERBB2IP; NF2; XPOT; NCK1; KARS; SUCLA2; EIF4G2; DCTN2; NAB1; SEPT7; STIM1; HMGN3; MRPL20; MRPL11; C11orf73; PFN1; HNRPA3P1; ATP5A1; OLA1; ATP5E; ZNF131; RIF1; CYCS; SUMO2; GOLPH3; TCEB2; ICMT; ALG6; MED23; SUCLG1; ZC3H4; BRD7; HSPBAP1; COX7A2L; MBTPS1; BAZ1B; DPM3; LOC645139; ZC3H11A; C1orf9; SKP1; SECISBP2; ELMO2; PSMA4; MRPS15; ASNSD1; AATF; LOC552889; ANP32E; C19orf56; MBD3; TPI1; TAF9; HSPE1; CFDP1; ALDOA; R3HDM1; NAT13; UQCRH; HSPC152; EIF2AK1; CSTF3; HMGN4; MFAP3; RNF4; DAZAP1; COPS5; ATXN10; SSBP1; CASC3; RPN2; FLJ11286; HNRNPR; HDAC7; UBP1; SUPT5H; YWHAQ; BCAS2; UBE4A; RBM14; NAP1L4; COX6B1; KHSRP; VPS45; SNRPG; LONP1; CDV3; FKSG30; HSPA8; RBM7; C1orf149; XRCC6; USP1; POLR1D; RPA1; DZIP3; TUBA1B; HNRPAB; TTC31; NSMAF; SERTAD2; PTOV1; LDHA; PSMD14; YBX1; NRD1; C2orf25; ATP5J2; ATP1A1; NSUN5B; GPBP1L1; LRRC40; CASD1; C16orf57; PSMD1; PCMT1; NUP93; YWHAH; PPT1; NDUFB4; NMI; PTCD3; HADH; NDUFA4; SMC3; NGRN; NFATC2IP; TDRD3; ANAPC13; hCG_1776980; TOMM70A; PATZ1; TRIM28; FTO; RAB5A; SACM1L; TDG; PCID2; EDEM3; TCP1; GTF2F2; ARMC1; GSPT1; ZC3HAV1; NDUFC2; ARF4; CCDC53; RUFY1; DDX1; SAFB2; THOC5; PUS3; ATF4; POLRMT; ZNF83; RANBP5; SLC25A44; DDX18; UBR5; ZMYM2; C1orf41; POLR3E; ZNF574; DGCR8; C11orf10; IHPK2; PSMD2; SNX4; EID1; TMPO; STK17A; LARP1; UNC50; HAX1; MAP2K2; SLC33A1; NDUFS8; TADA3L; TMED5; MGA; SUMO1; WDR59; LOC730107; KHDRBS1; FAM48A; LSM3; GOT2; CUL4A; NUP37; DGKZ; UBA5; NXT1; ATXN7; PSMD8; PRKRA; FBXW2; TERF2IP; CHCHD3; C16orf80; ILF2; MRPL3; LSM5; GATAD2A; TUBA1C; PPP1CC; RSAD1; TCEB1P; CTR9; HIBCH; SAMM50; WDR82; ACTL6A; MYOD1; FAM62A; SERP1; METTL5; DYNC1H1; CD46; ATP5G3; INPP4A; C20orf24; TCEB1; PRUNE; DLG1; CBX3; SAC3D1; IKBKAP; SLC25A46; UBA2; TTC27; SETMAR; MRPL9; DMXL2; C13orf23; LASP1; CEP57; PHF20; MEA1; BZW1; MPHOSPH10; CALM2; MRPL16; HDAC1; ERCC5; SCP2; TSPYL4; EPM2AIP1; AMD1; MDH1; NOLA1; GORASP2; CCBL2; CLEC3B; GPR89B; UBL5; VPS8; MAPK6; CSGALNACT2; MSL2L1; NOL11; TANK; NDUFA6; REXO2; FTHP1; ARHGAP15; AOF2; COX7C; EIF3M; TMOD1; OXSR1; FBL; MDM1; CDC16; JARID2; DGUOK; PSMC2; RTN4; POLR2H; METTL9; CUTA; C19orf2; TROVE2; TAF2; GCDH; GPR89A; TRIM33; EFTUD1; PSMB2; DOCK2; CHMP7; ADA; UBAP2; GIMAP6; MRPL48; TUBA1A; IDH2; SSR2; SNRPD2; NDUFB6; STAU1; MRPL13; SMARCAL1; TRIM37; MSH2; SLC2A3P1; ANP32B; MIF; ATP13A3; THEM2; UBE2V2; KIAA0494; SLC5A3; C16orf61; NDUFA13; PRR14; CLNS1A; CDC25B; AZI2; DNAJB6; RTN3; KIAA0241; NDUFA3; C19orf53; ZC3H13; TFG; C11orf48; SPAST; UFD1L; FBXO46; CDR2; NCOR2; HINT1; ATF2; ATMIN; ZDHHC4; C12orf11; LEPROTL1; SLC25A38; PCM1; HMG2L1; GTF3A; ST13; HYOU1; ATIC; UBR4; FAF1; RHOG; TMEM135; CNOT7; C1orf174; DRAP1; KIAA0152; INTS9; LDHB; RARRES3; FADS1; SEPT6; ABCD3; GRSF1; LCMT1; CENTB1; ABCC1; RAD21; FADS2; NUP50; NF1; TFPT; LOC150759; FDPS; THYN1; KCTD9; LSM4; KIFAP3; SSBP3; FDFT1; TM2D3; SC4MOL; EVL; FAM130A1; RCN1; PTP4A2; CD47; LANCL1; EXTL2; INSIG1; SEMA4D; LCP2; C9orf78; CMAH; NUCB2; HMGCS1 |
| 2nd_AML | SMARCD2 | <0.001 | 0 | + | up | 1500 | 10 | EXDL2; LMAN1; FKTN; ACD; MASP1; ARPC1B; C16orf33; POLR3K; IMPDH1; BLOC1S1 |
| T-ALL | SMARCA1 | <0.001 | 0 | + | up | 750 | 159 | CD74; HLA-DRA; HLA-DRB1; CD19; BLNK; HLA-DPA1; C14orf139; MEF2C; BTK; SNX2; JUP; TFEB; CTNNA1; RIPK2; HLA-DQB1; LAMC1; INPP5D; GGA2; CUL1; BCL11A; LAPTM5; PTPN18; NUBP1; ROGDI; GNG7; LILRA6; TRIM38; ENG; HLA-B; TLR1; PPP3CA; DYNLT1; SIPA1; CSRP2; CDK9; FHL1; KIAA0040; REEP5; C17orf60; KCTD15; HLA-DOA; DOK3; CIDEB; NUDT3; PRDX1; HLA-G; GRB10; CDKN1A; FPGS; MEF2A; GPX1; CYBA; IRF8; FES; CECR5; ATP6V0D1; RCBTB1; PSMB9; LDLRAP1; MEF2D; RBM38; C14orf106; C14orf113; PRKCD; CXorf21; ZBED1; RPS6KA1; TBC1D5; HIP1; RAB4B; NAV1; GALNT12; CLIP4; ASRGL1; FAM13A1; GPA33; HEG1; ITGAE; AZI2; WWOX; PLS1; RYK; CR2; TXK; ATHL1; CDR2; PALLD; KIF21B; FZD6; CD300A; RCBTB2; PAQR3; DENND1C; ZNF529; LRRC1; GOLGA8G; DEXI; ITPR2; WBP5; PDLIM5; PKIA; RASGRP1; OSBPL3; LOC440295; GNPDA1; S100A11; SSBP3; CHRNA5; ATP8A2; PLCG1; CLGN; CD1E; PCBP3; PIP4K2C; TRGV9; TRGC2; EXTL2; ZNF167; TARP; LIMA1; SNX10; SLC19A2; SELPLG; AGL; NOTCH3; C9orf78; VAT1; CMAH; ATP1B1; C14orf135; LZTFL1; CD247; EPHB6; CD2; CD6; GATA3; GALNT6; NGFRAP1; ITK; DENND2D; CHI3L2; CD28; AQP3; USP20; PTPN7; BIN2; LAT; BCL11B; PRKCQ; LCK; SCD; CD7; TRAT1; UBASH3A; CD3E; TRD@; MAL; SH2D1A; CD3D |
| Relapse | SMARCA2 | <0.001 | 0.01129 | + | down | 750 | 13 | DBN1; ZNF253; CDCA3; MAGED1; CDC25B; ERCC6L; HJURP; MYH10; SALL2; EXO1; IGFBP7; MFSD1; LEPROT |
| T-ALL | SMARCD1 | 0.001 | 0 | + | up | 2500 | 1018 | CD74; HLA-DRA; HLA-DRB1; PAX5; CD19; BLNK; HLA-DPA1; C14orf139; HLA-DPB1; HLA-DMA; HLA-DRB5; CD79B; MEF2C; CD24; TCL1A; BTK; CD79A; SNX2; VPREB3; JUP; PLCG2; CIITA; NCF4; POU2AF1; IGHM; CHD7; GALNAC4S-6ST; RIPK2; TCF4; HLA-DQB1; INSR; HLX; LAMC1; GGA2; STX7; SLC27A3; BCL11A; EGLN1; LAPTM5; PTPN18; LAT2; HLA-DMB; BANK1; TSPAN13; STX3; HLA-DRB6; PFTK1; CD9; CD22; PTPRE; BACH2; HCP5; GNG7; PTK2; RHBDF2; LILRA6; LILRA2; TRIM38; ENG; HLA-B; TLR1; tcag7.1314; FLJ20674; MARCH3; SIPA1; CSRP2; HLA-F; CDK9; FHL1; PDLIM1; KIAA0040; PIK3CG; STK32B; LILRB1; SOCS2; LARGE; NLRP1; TSPAN14; 3.8-1; FADS3; RGL1; ADCY6; PDE4B; FAM65A; LY86; ADCY9; C17orf60; KCTD15; HLA-DOA; ERO1LB; VPREB1; CTGF; HIST2H2AA3; DOK3; HLA-J; BTG2; FOXO1; PXDN; CLCC1; FAM49A; CIDEB; NUDT3; SCML2; C1orf38; HLA-G; SCARF1; MBP; SPINT1; GRB10; S100A13; KIAA1033; PSD3; DENND3; CDKN1A; CDYL; C10orf10; NEIL1; CRIM1; MYO5C; ALDH3A2; GAB1; GPX1; P4HA2; CYBA; LRRK1; MDM2; SYNGR1; SLC35D2; VIPR1; ELK3; LILRB2; WFS1; IRF8; OGFRL1; ECHDC3; BTN2A2; LILRB3; RAI14; ARSD; ETS2; KMO; SSH3; TGIF1; FES; GNA11; SCARB2; NPY; TCL6; MME; NPR1; KIF13A; SLC35E3; MYLK; IL1B; FAM50B; CSF2RB; TRMT12; GSN; HLA-DRB3; SPIB; MYLIP; TERF2; BCAR3; RAB13; NRIP1; RAG1; NCF1; PIP5K1B; RAPGEF3; DRAM; KLHL2; MANBA; TAP2; GNAI1; CD180; SCARB1; SLC2A5; TPST1; APP; LDLRAP1; DIP2C; PPFIBP1; DRP2; ARHGAP25; C14orf106; DACT1; KANK2; ARHGEF12; HHEX; S100A1; C14orf113; CLCN7; SPI1; WASF1; C14orf132; ZNF274; STAG3; FUCA1; TLR2; EPHB4; CELSR1; IRF7; LDOC1; DYRK3; HIST1H2BF; SYT11; COL5A1; HLA-DOB; SCHIP1; CXorf21; NUAK2; DGCR6; SLC16A2; OR2A9P; HIST1H2BG; PAOX; IRF6; C12orf49; F13A1; ACOT9; KLF11; CYP2B7P1; UNC93B1; OCA2; HLA-DQA1; ALOX5; PDK1; EWSR1; LIMD1; PCDH9; RNF19B; SERPINI2; EGFL7; ZNF193; ABCB4; TLE1; FGD2; EHD4; CYBB; ADRBK1; SMAD3; PCSK6; CRTAP; HIP1; ECM1; ACTR2; HLA-A; GSTM5; NAV1; HIST1H2BK; MGC5370; LMO2; SH2B2; PRO2268; UNC13B; PDE8B; CCR1; NCF2; SRRD; SFRS2IP; PPM1F; BMPR2; STAT2; ELL3; RIN2; COBL; MTSS1; KIAA1539; DUSP6; C9orf167; SGSH; C9orf45; SPPL2B; IFI35; LTB4R; MAN1A1; EMP2; CD200; NCOA3; CBR1; HIST2H2BE; BMP2; RASL10A; CRMP1; ENTPD1; FGD1; P2RY14; ERG; ZSCAN16; HCK; CDC25C; CTSS; SAMD4A; ST6GALNAC4; TNS3; JAM2; HIST1H3D; KHDRBS3; IRF1; SDC2; TNS1; SSX4; NRGN; HLA-DRB4; GABBR1; PBX2; HLA-E; SLC43A1; DUSP3; CRAT; KCNA5; ODZ4; SMTN; TRABD; GPR132; SERHL2; HIST1H2BE; FAM108B1; DDEF1; CAP2; JAK2; HIST1H2BN; MYRIP; IGSF3; DSP; OR1A1; MRC1; NOL4; CYB5R2; IFIT3; KCTD7; OLFML2A; LTBP2; FLT3; MBNL2; TBXA2R; B2M; HPCAL1; FOXP1; OR7A5; CEACAM21; HIST1H2BH; NIPSNAP3B; ENTPD3; SEMA5A; LTBR; MS4A1; CD86; CXCR6; TCF3; SEMA6D; CD40; SLC7A7; POLD4; APBB1IP; TBC1D9; EPHB3; IFIT2; BACE1; ATP10B; PGCP; SLC11A1; HIST1H2AM; ID3; CSHL1; NLRP2; GNAZ; HIST1H2AJ; GAS8; EFNB1; ADAM19; HIST1H1C; HSPA2; HOMER2; STAP1; EZR; IL8; SPRY4; IL13RA1; TP53I11; ASB13; PTPRG; DDR1; SEMA3F; NEBL; C5orf4; ADCK2; HIST1H4H; FLJ20489; RHOQ; ITIH3; BLK; ULK2; FER1L3; SPTLC2; ZNF467; TRPM2; SLC17A7; LDB2; FLOT2; CKMT2; ANKRD55; REEP1; ITGA5; LOH3CR2A; IL3RA; KIAA0774; ECM2; KIAA0746; CYP2B6; CLOCK; C10orf56; KCNJ2; C11orf24; FLJ10357; CENTA1; HIST1H2BI; GNG11; FKSG49; MYO10; DPEP1; TXNIP; MUC5AC; IRAK3; SH2D3C; INTS3; USP10; EPHA7; GJC1; IGLJ3; MLLT4; ALDOC; MGP; MLPH; ITIH4; IFI30; RBM47; UGCG; GIMAP4; BC37295_3; DNTT; ZNF117; SIGLEC15; UTRN; TBC1D16; EGFR; CLEC4E; AK5; PLVAP; FERMT1; ATP10D; SPTA1; NFATC4; OPHN1; STARD13; SLC2A1; FLT1; APBB2; SP4; TCL1B; TNFSF13; ALDH3B1; SEMA6A; SAMHD1; AQP1; SUOX; IGKV1OR2-108; C1orf78; PTGS1; CD52; CENTD1; ERBB2; MS4A6A; HIST1H2BD; EVC; HSPG2; HEY2; SYNPO; LILRB4; DKK1; HAP1; TGIF2; PSCD4; RBMS3; LRRC17; SCN1B; NDUFV2; PPM1G; PCGF1; MRPS34; PCNP; THOC7; SLC38A2; SMARCC1; NDUFAF1; SUPT6H; DYNC1I2; SF3B3; NOLC1; FAM89B; NCOR1; PIGH; POLR2B; FUBP3; MAPBPIP; C1D; EXOSC10; CHERP; UCRC; PRR4; C12orf41; SNRPE; ZNHIT1; USP47; PARL; POP4; C1orf77; PIH1D1; CHMP2A; SLC20A1; SLC30A9; ACACA; YY1; TSN; YARS; FAM60A; UBE3A; LUC7L2; UQCRFS1; CRKL; PANK2; STAMBP; GPI; RPL24; RAB7A; BXDC5; KIAA0528; GNG5; R3HDM2; ACAT2; PSMC4; CYFIP2; QRICH1; PSMB7; TPRKB; CCT7; HSP90AA1; PI4KA; HNRNPA2B1; SLC4A7; PSCD2; SETD5; TARDBP; REST; PPP1R7; PAAF1; YLPM1; SFRS9; COX7A2; FAM128B; HYPK; TBCE; MORF4; MAN1A2; TMCO1; NDUFA9; PIK3R4; UBB; DHPS; NFYB; BTBD1; POLR2I; RBBP4; COPG; VPS13B; ARS2; SGSM2; BMS1; ADRM1; H2AFV; HSPA9; CCT2; C19orf50; LOC130074; NOSIP; ADSS; PSMA7; YY1AP1; MRPL22; ZXDC; CTCF; C15orf44; C19orf29; UQCRC1; TIMM17A; NPEPPS; TXNL1; SCAMP1; ACLY; JOSD1; BCKDHA; H3F3A; ZBTB11; COX6A1; ALG8; DRG1; FBXO7; PAIP1; NDUFB7; SRP72; WDR6; ISG20L2; DCTN6; SRP9; COX4NB; CHCHD2; C9orf82; B3GAT3; BCL7B; PSMA5; CCDC72; UQCRQ; C19orf60; C14orf166; ERBB2IP; NF2; XPOT; KARS; EIF4G2; DCTN2; SEPT7; NDUFB8; SNRPD1; POLR2F; MRPL20; MARCKSL1; PFN1; HNRPA3P1; ATP5A1; OLA1; ATP5E; ZNF131; CYCS; SUMO2; GOLPH3; TCEB2; FASTKD1; ICMT; PGD; ALG6; PPME1; SUCLG1; ZC3H4; BRD7; SNRPB; MORF4L2; NSUN5C; HSPBAP1; COX7A2L; MBTPS1; BAZ1B; DPM3; LOC645139; FLJ11506; C1orf9; SKP1; SECISBP2; ELMO2; PSMA4; MRPS15; ASNSD1; AATF; LOC552889; ANP32E; ATP5H; MBD3; TPI1; FLJ14154; TAF9; HSPE1; CFDP1; ALDOA; R3HDM1; NAT13; SHMT2; UQCRH; HSPC152; EIF2AK1; STX10; HMGN4; BTG3; RNF4; DAZAP1; COPS5; ATXN10; SSBP1; CASC3; C18orf10; RPN2; HNRNPR; UBP1; YWHAQ; BCAS2; UBE4A; RBM14; NAP1L4; COX6B1; KHSRP; VPS45; SNRPG; LONP1; CDV3; EIF4E2; C1orf149; XRCC6; USP1; POLR1D; RPA1; MDH2; TUBA1B; HNRPAB; FRG1; NSMAF; USP39; DTYMK; PTOV1; LDHA; PSMD14; YBX1; NRD1; C2orf25; ATP5J2; ATP1A1; COX8A; HSD17B7; SPINT2; COX17; LRRC40; C16orf57; PSMD1; PCMT1; NUP93; NDUFB4; PTCD3; HADH; DDX39; NDUFA4; GMPS; METT11D1; NGRN; NFATC2IP; ANAPC13; hCG_1776980; TOMM70A; PATZ1; TRIM28; FTO; TDG; PCID2; TCP1; GSPT1; NDUFC2; TXNL4A; ARF4; CCDC53; RUFY1; DDX1; MAGOHB; MTX1; SAFB2; NUBP2; THOC5; ATF4; POLRMT; ZNF83; RANBP5; SLC25A44; DDX18; ZMYM2; C1orf41; PRPF19; POLR3E; DGCR8; C11orf10; IHPK2; PSMD2; SNX4; EID1; TMPO; LARP1; UNC50; HAX1; MAP2K2; NDUFS8; KIAA0999; TMED5; PREB; SUMO1; LARS2; WDR59; SNRPF; LOC730107; KHDRBS1; LSM3; GOT2; CUL4A; BOLA2; MTHFD2; NUP37; SF3B5; UBA5; NXT1; HK1; SHFM1; PSMD8; PRKRA; ATP5SL; FBXW2; C9orf16; BIRC2; TERF2IP; CCDC86; CHCHD3; C16orf80; ILF2; MRPL3; LSM5; GATAD2A; TUBA1C; PPP1CC; RSAD1; DPP3; TCEB1P; NIT2; CTR9; HIBCH; SAMM50; GARS; WDR82; ACTL6A; MYOD1; CEP63; SERP1; LSM1; METTL5; SRPK1; DYNC1H1; ATP5G3; PIN1; INPP4A; C20orf24; TCEB1; PRUNE; DLG1; CBX3; SAC3D1; NDUFS6; IKBKAP; SLC25A46; UBA2; TTC27; MRPL9; PHF20; MEA1; BZW1; MPHOSPH10; CALM2; MRPL16; HDAC1; ERCC5; OSBP; CRY1; SCP2; TSPYL4; EPM2AIP1; AMD1; MDH1; GORASP2; CCBL2; CLEC3B; DBF4; GPR89B; UBL5; VPS8; MSL2L1; DECR1; NOL11; NDUFA6; NDUFA7; AOF2; COX7C; EIF3M; GSS; TMOD1; FBL; MDM1; CDC16; JARID2; DGUOK; PSMC2; RTN4; POLR2H; METTL9; CUTA; COX6C; TAF2; GCDH; GPR89A; TRIM33; EFTUD1; PSMB2; DOCK2; PPM1A; ADA; VAPA; UBAP2; MRPL48; TUBA1A; IDH2; SSR2; SNRPD2; NDUFB6; STAU1; VDAC3; LETM1; SPECC1L; MRPL13; SMARCAL1; TRIM37; MSH2; SLC2A3P1; ANP32B; ALG9; MIF; ATP13A3; THEM2; KIAA0494; C16orf61; NDUFA13; RPIA; PRR14; ITGAE; CLNS1A; CDC25B; RTN3; KIAA0241; NDUFA3; C19orf53; ZC3H13; SEC61G; TFG; C11orf48; SPAST; UFD1L; FBXO46; CDR2; NCOR2; EXOSC8; MTMR6; HINT1; SLC3A2; ATF2; ATMIN; ZDHHC4; LEPROTL1; ADH5; SLC25A38; PCM1; HMG2L1; MRP63; ST13; HYOU1; ATIC; UBR4; AACS; GMDS; FAF1; CNOT7; C11orf51; PMF1; MTMR2; DRAP1; COX11; KIAA0152; INTS9; SEC61B; LDHB; FADS1; ABCD3; DCUN1D1; GRSF1; LCMT1; DNAJC9; RAD21; FADS2; NUP50; TFPT; SFI1; FDPS; C11orf49; THYN1; LSM4; KIFAP3; SSBP3; FDFT1; RCN1; CLPP; PTP4A2; SLC1A4; CD47; LANCL1; EXTL2; SEMA4D; CDK5RAP3; AGL; C9orf78; NUCB2; HMGCS1 |
| TEL-AML1 | SMARCA4 | <0.001 | 0 | + | up | 400 | 54 | PTGES3; IQGAP2; MYC; PRPS2; CD44; C15orf39; CASP1; C11orf24; ANXA2; MSL3L1; CTNND1; NAGPA; NDRG1; DYRK4; S100A4; ALDH3B1; GPM6B; CLASP1; CHD9; ZNF672; DBN1; NUP205; SEPHS1; TMCC1; GYS1; FOXO1; GTF2IRD1; FAM65A; CUX1; ITPR3; SMARCA4; HMG20A; RICS; RNF31; TXNRD1; DNMT3A; ZDHHC3; VAV1; EIF2AK3; ZNF91; KNTC1; NARFL; WASF2; RASA4; LBA1; SCARB1; RY1; PTP4A3; NRN1; ARHGAP29; TNFRSF21; ABHD3; TCFL5; TNS1 |
| CCR | SMARCA4 | <0.001 | 0 | + | down | 1000 | 29 | TSPAN32; S100A4; MLC1; GREM1; LGALS1; CCPG1; LTBR; CCNA1; MATK; MAP3K5; ATP8B4; MGST2; SERPINB1; ABHD4; NLRP3; CD302; MYO1F; MAP7; ARTN; PTGES3; HK2; SFXN3; ORC2L; ZNF107; ZNF273; ZNF43; ANP32E; ZNF675; DBN1 |
| T-ALL | SMARCAL1 | <0.001 | 0 | + | up | 400 | 67 | CD74; HLA-DRA; HLA-DRB1; PAX5; CD19; BLNK; HLA-DPA1; HLA-DPB1; HLA-DMA; HLA-DRB5; CD79B; MEF2C; CD24; TCL1A; VPREB3; JUP; POU2AF1; IGHM; CHD7; TCF4; HLA-DQB1; INSR; GGA2; EGLN1; LAPTM5; PTPN18; HLA-DRB6; BACH2; GNG7; tcag7.1314; FLJ20674; LARGE; 3.8-1; FADS3; FAM65A; HLA-DOA; ERO1LB; FOXO1; GTF2IRD1; C10orf10; NEIL1; MYO5C; ALDH3A2; GAB1; MDM2; VIPR1; RAI14; SSH3; LDHB; DCUN1D1; GRSF1; LCMT1; RAD21; NUP50; ETFB; FDPS; SLC25A20; THYN1; LSM4; TXN; RCN1; CD47; LANCL1; IMPA1; SEMA4D; FAH; C9orf78 |
| Hyperdip>50 | SUV39H1 | <0.001 | 0 | + | up | 1000 | 188 | VAV3; EML4; ABCC4; GPATCH2; EWSR1; KIAA0564; PIGB; PLCB1; GCLC; ELF1; PDE8A; TPD52; STAM2; RPS6KA2; SENP7; MCTP2; LRRC8B; E2F5; PCDH9; FPGT; DENND4A; PRKACB; GLS; CD84; C20orf30; MAGED4B; CST3; SAMSN1; SLC46A3; C9orf45; RHOBTB3; PAM; FCGR3B; ZNF364; HIPK1; CBLB; DLEU1; MINA; BTG1; MRPL35; TNFRSF21; NIPSNAP3B; ENPP4; AGPS; TMEM38B; GIMAP6; PTPRD; TCL6; SERPINA1; PIK3R1; GNS; APC; FADS3; MARCH7; TES; PIP5K1B; NUDT4; F11R; GIMAP5; SLC7A5P1; C10orf6; RBMX2; DEK; NDUFA1; PSMG2; SMS; USP11; PTP4A1; E2F3; CBARA1; SGCB; C4orf27; RANBP9; DKC1; SAP30BP; CCT8; ATP5J; FANCE; STX8; PGRMC1; BDH2; UXT; MID1IP1; KIF4A; C11orf67; VBP1; WRB; MORF4L2; ING2; CANT1; SSR1; UBL4A; TBC1D25; PIP4K2B; C10orf119; HMGB3; FBXO9; PRPS2; PRPS1; PSMG1; RBBP7; CASP7; HMG4L; BAG5; SLC43A3; PHF10; ZMYM3; VRK1; ARMCX6; TBP; C21orf45; FMR1; PHF3; EBP; PARP2; NGDN; KDSR; TK1; CCDC22; RPL10L; ZNF673; LOC390183; MCTS1; ERCC6L; GLA; HMGN1; C21orf66; PGK1; SUMO3; SLC35A2; DONSON; NDUFB11; F8A1; BRCC3; NSDHL; DYRK1A; SUV39H1; RAB9A; PRDX4; PDHA1; ZBED1; HDAC6; UCHL5IP; M-RIP; CETN2; ORC3L; WDR7; TIMM17B; STAG2; HUWE1; MTMR1; PHKA2; PIN4; ACOT2; SLC19A1; CHAF1B; PQBP1; ARMCX5; MPP1; POLA1; GSPT2; UBE2A; MAGEH1; LAS1L; RRP1B; MGC39900; GPKOW; HNRPH2; SOD1; UBQLN2; SLC9A6; FTSJ1; USP9X; ARMCX1; RNF113A; MED12; MTCP1; DHRS4; RP6-213H19.1; PSMD10; TCEAL1; HCCS; GPRASP1; SCML2; TCEAL4; C14orf147; UPF3B; MORC4 |
| CCR | SUV39H1 | <0.001 | 0 | + | up | 750 | 17 | AHNAK; BAALC; SECTM1; CDC42EP3; PDE8A; GBP2; FOXP1; KLF7; SLC35D2; MKI67; HJURP; ARHGAP19; MCM10; TOP2A; MAGED1; ANP32E; HMG4L |
| T-ALL | CBX3 | <0.001 | 0 | + | up | 1500 | 608 | CD74; HLA-DRA; HLA-DRB1; PAX5; CD19; BLNK; HLA-DPA1; C14orf139; HLA-DPB1; HLA-DMA; HLA-DRB5; CD79B; MEF2C; CD24; TCL1A; BTK; CD79A; VPREB3; JUP; PLCG2; CIITA; NCF4; POU2AF1; TFEB; IGHM; CHD7; GALNAC4S-6ST; RIPK2; TCF4; HLA-DQB1; INSR; HLX; STX7; SLC27A3; EGLN1; LAPTM5; PTPN18; LAT2; HLA-DMB; BANK1; STX3; HLA-DRB6; PFTK1; CD9; PTPRE; BACH2; HCP5; GNG7; PTK2; RHBDF2; LILRA6; LILRA2; TRIM38; ENG; HLA-B; TLR1; tcag7.1314; FLJ20674; HLA-F; FHL1; PDLIM1; KIAA0040; STK32B; LILRB1; CSDA; SOCS2; LARGE; NLRP1; 3.8-1; FADS3; RGL1; ADCY6; PDE4B; FAM65A; LY86; TULP4; ADCY9; C17orf60; KCTD15; HLA-DOA; ERO1LB; UNC119; VPREB1; CTGF; HIST2H2AA3; HLA-J; GALNT14; CIDEB; SCML2; C1orf38; HLA-G; SCARF1; MBP; SPINT1; S100A13; DENND3; C10orf10; NEIL1; CRIM1; MYO5C; ALDH3A2; GAB1; P4HA2; LRRK1; MDM2; SLC35D2; VIPR1; ELK3; LILRB2; WFS1; IRF8; CPM; OGFRL1; ECHDC3; BTN2A2; LILRB3; TSC22D3; RAI14; ARSD; KMO; SSH3; FES; GNA11; TCL6; MME; NPR1; KIF13A; SLC35E3; MYLK; IL1B; FAM50B; CAST; TRMT12; HLA-DRB3; SPIB; MYLIP; TERF2; BCAR3; RAB13; NCF1; PIP5K1B; RAPGEF3; DRAM; KLHL2; TAP2; GNAI1; CD180; SLC2A5; TPST1; LDLRAP1; DIP2C; PPFIBP1; DRP2; DACT1; KANK2; ARHGEF12; HHEX; S100A1; C14orf113; C14orf132; STAG3; PHACTR1; TLR2; CELSR1; IRF7; LDOC1; HIST1H2BF; SYT11; COL5A1; HLA-DOB; SCHIP1; CORO2B; NUAK2; DGCR6; SLC16A2; OR2A9P; HIST1H2BG; IRF6; C12orf49; F13A1; CYP2B7P1; UNC93B1; OCA2; HLA-DQA1; ALOX5; PDK1; LIMD1; PCDH9; RNF19B; SERPINI2; EGFL7; ZNF193; ABCB4; FGD2; CYBB; ADRBK1; SMAD3; PCSK6; CRTAP; HIP1; ECM1; ACTR2; HLA-A; GSTM5; NAV1; MGC5370; SH2B2; PRO2268; UNC13B; PDE8B; CCR1; NCF2; BMPR2; STAT2; ELL3; RIN2; MTSS1; C9orf167; SGSH; C9orf45; SPPL2B; LTB4R; EMP2; CBR1; BMP2; RASL10A; ENTPD1; FGD1; P2RY14; CDC25C; SAMD4A; JAM2; HIST1H3D; CCDC81; KHDRBS3; SSX4; NRGN; HLA-DRB4; GABBR1; PBX2; DUSP3; KCNA5; ODZ4; GPR132; SERHL2; HIST1H2BE; CAP2; HIST1H2BN; IGSF3; DSP; OR1A1; MRC1; NOL4; IFIT3; OLFML2A; LTBP2; FLT3; TBXA2R; FOXP1; OR7A5; CEACAM21; ENTPD3; SEMA5A; LTBR; MS4A1; CD86; CXCR6; SEMA6D; CD40; TBC1D9; EPHB3; IFIT2; ELOVL2; BACE1; ATP10B; SLC11A1; HIST1H2AM; CSHL1; NLRP2; HIST1H2AJ; GAS8; EFNB1; ADAM19; HIST1H1C; HSPA2; HOMER2; IL8; SPRY4; IL13RA1; TP53I11; ASB13; PTPRG; DDR1; SEMA3F; NEBL; C5orf4; ADCK2; HIST1H4H; FLJ20489; RHOQ; ULK2; FER1L3; ZNF467; SLC17A7; LDB2; CKMT2; ANKRD55; REEP1; ITGA5; LOH3CR2A; IL3RA; KIAA0774; ECM2; KIAA0746; CYP2B6; C10orf56; KCNJ2; HIST1H2BI; FKSG49; MYO10; DPEP1; TXNIP; MUC5AC; IRAK3; SH2D3C; USP10; EPHA7; GJC1; MGP; MLPH; ITIH4; IFI30; C6orf145; RBM47; UGCG; TGFB1I1; GIMAP4; BC37295_3; ZNF117; SIGLEC15; UTRN; EGFR; CLEC4E; AK5; PLVAP; FERMT1; NFATC4; OPHN1; STARD13; FLT1; SP4; TCL1B; TNFSF13; ALDH3B1; KCNMB1; SEMA6A; KIF1B; SAMHD1; AQP1; SUOX; IGKV1OR2-108; PTGS1; CD52; ERBB2; MS4A6A; EVC; LILRB4; DKK1; HAP1; RBMS3; LRRC17; SCN1B; LRRC40; ZNF146; PSMD1; PCMT1; NUP93; NDUFB4; PTCD3; HADH; DDX39; NDUFA4; SMC3; METT11D1; NGRN; ANAPC13; hCG_1776980; TOMM70A; PATZ1; TRIM28; RAB5A; SACM1L; TDG; TMEM208; PCID2; TCP1; ARMC1; NDUFC2; TXNL4A; ARF4; CCDC53; DDX1; MAGOHB; MTX1; SAFB2; THOC5; ATF4; RANBP5; DDX18; UBR5; ZMYM2; C1orf41; PRPF19; POLR3E; DGCR8; C11orf10; IHPK2; PSMD2; SNX4; EID1; TMPO; LARP1; UNC50; UQCR; NDUFS8; PREB; SUMO1; SNRPF; LOC730107; KHDRBS1; LSM3; GOT2; CUL4A; BOLA2; NUP37; SF3B5; NXT1; STMN1; SHFM1; PSMD8; PRKRA; ATP5SL; FBXW2; SLC39A6; BIRC2; TERF2IP; CHCHD3; C16orf80; ILF2; MRPL3; LSM5; GATAD2A; TUBA1C; PPP1CC; TCEB1P; CTR9; MLF1IP; HIBCH; SAMM50; GARS; WDR82; ACTL6A; MYOD1; SERP1; LBR; LSM1; METTL5; RFXANK; SRPK1; DYNC1H1; ATP5G3; PIN1; C20orf24; TCEB1; PRUNE; CBX3; SAC3D1; NDUFS6; IKBKAP; SLC25A46; TMEM14A; UBA2; MRPL9; UNG; PHF20; SUB1; BZW1; CALM2; MRPL16; HDAC1; SCP2; TSPYL4; EPM2AIP1; MDH1; NOLA1; GORASP2; CLEC3B; DBF4; GPR89B; UBL5; KEAP1; MSL2L1; DECR1; PAICS; NOL11; NDUFA6; NDUFA7; AOF2; COX7C; EIF3M; TMOD1; OXSR1; FBL; MDM1; DGUOK; PSMC2; RTN4; POLR2H; METTL9; CUTA; COX6C; TAF2; RFC3; GCDH; GPR89A; TRIM33; EFTUD1; PSMB2; VAPA; UBAP2; MRPL48; TUBA1A; IDH2; SSR2; SNRPD2; NDUFB6; HLTF; STAU1; VDAC3; SPECC1L; MRPL13; TRIM37; MSH2; SLC2A3P1; C5orf13; ANP32B; MIF; ATP13A3; THEM2; UBE2V2; KIAA0494; C16orf61; NDUFA13; RPIA; CLNS1A; KIAA0241; ZBED4; NDUFA3; C19orf53; ZC3H13; SEC61G; TFG; C11orf48; SPAST; UFD1L; EXOSC8; MTMR6; ATF2; ATMIN; ZDHHC4; LEPROTL1; ADH5; SLC25A38; PCM1; HMG2L1; MRP63; GTF3A; ST13; ATIC; UBR4; FAF1; CNOT7; C1orf174; C11orf51; PMF1; COX11; KIAA0152; SIVA1; INTS9; SEC61B; LDHB; DCUN1D1; GRSF1; LCMT1; DNAJC9; RAD21; NUP50; TFPT; FDPS; LSM4; PTP4A2; CD47; LANCL1; SEMA4D; MLLT11; C9orf78; C14orf135; NUCB2 |
| T-ALL | CBX1 | <0.001 | 0 | + | up | 400 | 93 | CD74; HLA-DRA; HLA-DRB1; CD19; BLNK; HLA-DPA1; HLA-DPB1; HLA-DMA; HLA-DRB5; CD79B; CD79A; SNX2; JUP; CHD7; GALNAC4S-6ST; RIPK2; HLX; INPP5D; STX7; SLC27A3; EGLN1; PTPN18; LAT2; BANK1; TSPAN13; STX3; ENG; TLR1; GAB2; CDK9; PDLIM1; STK32B; CSDA; SOCS2; LARGE; ADCY6; PDE4B; HIST2H2AA3; CLCC1; CIDEB; C1orf38; S100A13; DENND3; C10orf10; MYO5C; GAB1; GPX1; LRRK1; MDM2; SYNGR1; SLC35D2; ELK3; OGFRL1; ECHDC3; LILRB3; CAPN3; SSH3; SCARB2; SIVA1; RAP1GDS1; SEC61B; HDAC4; DNAJC9; PKIA; RAD21; RASGRP1; NUP50; TNFAIP8; FDPS; C11orf49; SLC25A20; THYN1; KCTD9; LSM4; HN1; SRPK2; ECT2; ELOVL5; ARL4C; PAFAH1B3; PTP4A2; INSIG1; MLLT11; VAT1; LZTFL1; NUCB2; HMGCS1; STAU2; NBR1; LAT; BCL11B; LCK; SCD |
| MLL | CBX4 | <0.001 | 0.02215 | + | up | 400 | 81 | MGC29506; CD52; MYO5C; ZSCAN18; EXDL2; SV2A; SERPINB9; DNTT; TNFRSF14; PHF15; ZNF135; GFOD1; ALOX5; ITGA5; NEDD4; SCHIP1; C11orf75; IL1B; DUSP26; ID3; ARMCX2; GIMAP4; DAB2; MYO1B; YES1; FGD1; NAV1; RYK; tcag7.1314; ZNF467; SUOX; CASK; SETBP1; MGC5370; EFNA1; RGL1; PRO2268; SALL2; PLCH1; GPR56; ZNF667; TREML2; SPAG16; CEP68; GIMAP6; RAPGEF3; C10orf10; CCND2; PECAM1; C6orf32; ITGA6; DAPK1; RSBN1; LST1; SERPINB6; A2M; CS; BCL7A; M6PRBP1; DGKD; GLT25D1; TBCC; TBC1D9B; APEH; EP400; MYH9; GMIP; RAP2A; GLUD1; CLASP2; BCAT1; ZNF394; MAP4; ASXL1; SIRT7; IDUA; PRKCE; ZEB2; CHPT1; C1orf164; RHOBTB3 |
| CCR | CBX5 | 0.002 | 0 | + | down | 1000 | 12 | TSPAN32; CCPG1; CDC42EP3; GBP2; KLF7; SLC35D2; MKI67; TMEM48; HJURP; MCM10; TOP2A; ANP32E |
| Hyperdip>50 | MYST2 | <0.001 | 0.04189 | + | up | 750 | 100 | OR7E38P; RAG2; DALRD3; EWSR1; EPB41L2; AEBP1; NCKAP1L; BCL2L1; CD38; AKAP12; FAAH; HPS4; SYK; MCTP2; E2F5; PCDH9; RHOBTB1; CD84; VEGFB; TUSC2; CST3; ARNTL2; IL7R; NME3; DIP2C; FCGR3B; HIPK1; ZNF124; PRKAB1; ALDH4A1; MT1E; KCNMB3; SGSM3; ORAI2; LOC91316; NKTR; FLJ13769; MAZ; TM9SF1; OGT; SLC35A1; PRPS1; RBBP7; BAG5; C4orf15; C4orf41; BACH1; LAMP2; FMR1; PHF3; SIRT1; ACSL4; NGDN; RDH11; MCTS1; LMBRD1; HMGN1; VPS26A; ALDH6A1; USP16; VAMP7; PGK1; SUMO3; DYRK1A; MSN; TTC3; MARCH5; SFRS15; PDHA1; SH3BGRL; HDAC6; WDR7; UBL3; STAG2; HUWE1; TMEM164; CPD; CUL4B; ARMCX5; RBPJ; POLA1; C21orf33; CXorf45; ATP6AP2; MAGEH1; LAS1L; RRP1B; MORC3; HNRPH2; ABCB7; UBQLN2; CYB5A; PHB; FTSJ1; USP9X; RNF113A; PIGP; RP6-213H19.1; PSMD10; UPF3B |
| TEL-AML1 | MYST3 | 0.004 | 0 | + | up | 2500 | 558 | ADK; PTGES3; SORD; AKAP1; GPX7; IQGAP2; C22orf9; ATP13A2; MYC; PRPS2; ACVR1B; KIAA0564; GALNT2; PUS7; CD44; PTGER4; UCK2; MINA; NOL14; ITM2C; CCDC86; RRS1; AP1S2; C15orf39; DIAPH2; C11orf24; LPXN; ANXA2; DPH4; PKIG; NUP210; EIF4EBP1; IMPA2; VIM; DKFZp667G2110; ZBTB24; MPZL1; CTNND1; ZNF593; PCBD1; SYNJ2; MT1X; FLJ11184; CDKN2D; MAPKBP1; MT1L; POLR3D; CCDC69; FAM98A; MGST3; NIP7; NRXN2; ZMAT3; CTSC; CIB1; FXN; MT1H; ASAHL; PTPN6; TTLL12; NCF1; FAM53B; PUS1; NFATC1; INPP1; CCDC94; SMPD2; TAGLN2; MT1P2; S100A4; FCHO1; BCAT1; C9orf16; BNIP1; MT2A; ANXA2P2; GRAMD4; LRMP; ALDH3B1; PPAT; PBX3; ENDOD1; IPO4; APOLD1; ABCB4; C12orf44; CD96; FABP5; GPM6B; FAIM; KLHL21; GLUD1; VPREB1; CSRP1; MT1G; CXorf21; CLEC11A; NLE1; RRBP1; EXOSC5; STS; PDLIM1; FASN; CLEC2D; FAM26B; RHBDF2; TMEM121; GLRX2; RPL27; LIMD2; SCPEP1; HDAC9; TRIB2; CD86; LDLRAP1; DET1; TERT; XYLT1; ME3; SERPINB8; NR1H2; LOC92482; MT1F; TREX1; MAP3K1; SYT11; TLR2; CHST6; IGFBP7; ATP1A3; NR1D2; PTER; CCDC81; P2RX5; AHR; SCO2; GRB2; SAMD4A; TNK2; FLNA; ARL2; BOP1; RIMS3; YWHAE; MATK; TBC1D8; BSPRY; ABCB1; VDR; IL17RA; KLHL2; RNF24; GTPBP3; LYN; RPS6KA1; C12orf10; ADARB1; DCPS; C20orf27; BIK; SEMA4A; ARF6; CD248; C11orf21; XTP3TPA; MKL1; RPP40; LTK; BLK; LOC391132; DAK; C21orf91; ENC1; MT1E; CD320; OGG1; SPARC; PLGLB1; dJ222E13.2; EMR2; KANK1; LRRFIP2; SIT1; LGALS1; GSTM4; TRPV2; MRPL12; APEH; CAST; CPM; NUAK2; RCC1; CD48; EHD4; RHOQ; BCL6; SPIB; CLN5; SPON2; RABIF; HCRP1; HK2; IQSEC1; CD9; TSPAN32; PLCB1; NP; CD84; LOC100137047-PLA2G4B; RBM47; SCLY; ECHDC2; ARHGEF11; KCNA3; JARID1C; DOK1; FAM125B; QTRT1; ALCAM; CTNNBL1; FRAT2; PDGFA; MS4A6A; PPCDC; ENDOG; TSEN34; CTSO; SIDT2; SETD6; EXOC7; MRTO4; GLDC; FOSL2; S100A10; PLXNC1; ATP2B1; C6orf62; RANGAP1; SLC29A1; PTGES2; RPS11; MYT1L; H6PD; EAF2; TBX21; RIN2; VNN2; GLT25D1; APOBEC3F; BAG2; SET; ARPC4; NLRP3; FAM30A; FGR; RPL27A; TMEM106B; SPPL2B; PRKCD; KIAA0125; MAN2B1; RGS16; RAP2A; CCDC14; CENPB; IL13RA1; KLRK1; CSF1R; RNASE6; ANXA6; C9orf9; NAGLU; TRIM8; QPRT; COL4A3BP; TUSC4; TNPO2; MBNL1; MXD4; SLC2A6; PYCR1; ITGB7; PIGB; ERP29; ITPKB; MPEG1; PRKCE; RHOB; ZNF239; DFNA5; PSCDBP; C14orf101; C20orf103; EIF2C3; RNF144A; KIF1B; CAMKK2; CTBP1; BDH1; SMAD3; CAV2; PPP2R1B; KLHL23; SNTB1; S100A11; OSBPL1A; CLEC7A; TPBG; HIP1; TBXA2R; SAMHD1; SLAMF1; DSCR3; ANGPT2; RRP9; FGF9; UST; LAMA2; LAT2; BLCAP; FAM60A; RNASEN; ZMYND11; AARS; ATP2B4; MCFD2; DNAJA1; POMT1; UBE3C; CCNL2; BTN3A1; CLINT1; ADNP; FUT1; MGC29506; SIN3B; C1RL; RBM5; WSB2; SYNJ1; PCAF; ZNF12; CCNT2; ZKSCAN1; CTDSP2; PSME4; SNAP23; STAT5B; ZMYM3; RNF38; PPP1R12A; PEX19; PHF2; FLJ10404; EIF1B; LGALS8; RING1; POLB; C11orf75; NIT1; YTHDF1; C20orf11; LUC7L; TCF7L2; HECA; DNAJB4; ZNF675; DNAJC10; DMXL2; ZCCHC8; FLJ10213; KIAA0174; CALM3; UBE2I; PQLC1; ITGB1BP1; NARF; H2AFV; RUFY3; PHTF1; RSBN1; HNRNPA0; ZMYM1; PUM1; SETD1B; MAN1A1; ZMYM2; RAB3GAP1; ZNF273; MAGED2; TLOC1; LAPTM4A; SH3TC1; SNRPN; ZNF43; CRKL; KBTBD2; C12orf35; CHD1; RAB1A; NT5C2; IFRG15; POLS; MAPK6; C1orf165; SCMH1; MYST4; RSRC2; YTHDC2; RAP2C; GDI1; MSRB2; KIAA0265; RYK; PCGF3; PVRIG; WDR37; MECP2; IPW; ZNF281; KIAA0515; SSBP2; GTF2E1; BRD1; TNKS; KIAA0317; MRPL49; NASP; SPTBN1; PBXIP1; SFRS8; GOLGA8A; UPF2; PRKX; PRKACA; C10orf18; STMN1; PHF1; BNIP3L; FOXK2; PIP5K1C; CREBBP; PPM1B; SPTAN1; MAPK14; HRB; ACSF2; RIT1; RHOF; YES1; PATZ1; ARFIP1; POU2F1; RAB22A; HIST1H2BD; ZMIZ1; MTF2; ZNF107; NBPF1; N4BP1; CAB39; ALDH5A1; GAK; MYST3; RBMS1; TRAF5; CLSTN1; OXSR1; YEATS2; DCP2; PLCG1; KIAA0232; BTBD3; CD200; DPYSL2; CCDC93; PARC; SRBD1; MAP4K3; ACSL3; ARHGEF18; REL; WSB1; ENTPD4; MAPK8IP3; RAPGEF2; FBXW7; ZNF217; CLASP1; CHD9; CENTB2; JMJD2B; POLE; NUP205; SEPHS1; PLCL2; MLXIP; SERINC5; VAMP5; FAM134B; FHIT; LEMD3; TMCC1; FAM50A; B4GALT6; SLC35E3; CUX1; AIF1; MKL2; GNPTAB; INTS3; PIK3IP1; ABHD10; SMARCA4; SETD4; UBE2E3; FCGRT; GRINA; FKBP1A; HMG20A; ISG20; BMP2; NMT2; ELK3; ITPR1; TRIM24; RNF31; TXNRD1; FYB; ZDHHC3; VGLL4; EDEM1; RECK; PRKCB1; VAV1; EIF2AK3; ZNF91; CHCHD7; WASF2; RASA4; RY1; SPTA1; ENPP4; CYB5R2; WDFY3; PCCA; DSG2; C13orf18; IDI1; FUCA1; FCHSD2; CBFA2T3; PIK3C3; NR3C2; ARHGAP29; ABHD3; TCFL5; C10orf26 |
| Normal | MBD2 | <0.001 | 0 | - | up | 1500 | 63 | NDUFA1; PSMA7; MRPL34; COPS3; COX6C; COX7B; FRG1; RBBP7; PSMC5; PSMB3; UBE2A; ATP5J; UCRC; TIMM8B; ARPC5; ENSA; MAPBPIP; SLC25A5; NDUFB8; EEF1E1; ATP5G1; EIF4A3; NDUFS6; RAN; COX6A1; SFRS2B; PSMD2; SF3B5; PGAM1; IQGAP1; MDH1; NDUFS1; PSMA3; HSPA5; MCTP2; TP53TG3; CCR5; APOL6; GRIK2; MXI1; RASL10A; EGFL7; KAL1; AKAP12; GNG11; FLRT3; EFR3B; PRKG2; ZNF239; ZNF192; CSHL1; CDH11; REEP1; FLT1; PCDH9; PCBP4; FER1L3; SLC6A16; AGMAT; PBX2; TXNRD3; MPPED2; HEY2 |
| E2A-PBX1 | MBD1 | <0.001 | 0 | - | down | 1500 | 343 | MSN; CTDSP2; LASP1; AMZ2; OGT; TMED10; CNDP2; SAPS2; MORC3; PLEKHM1; C14orf1; SEL1L; UBE4A; EXOC1; CSNK1D; PPP2CB; C14orf159; GMFB; KIAA1109; FAM134C; SFRS18; RPN2; CLINT1; CRLF3; ATP6AP2; UBE2D3; LAMP2; POFUT2; PAFAH1B1; UNC50; RPL17; RP6-213H19.1; ARL8B; SSR4; WSB1; SERINC1; ZNF706; TM9SF1; GMPR2; TMEM131; ZMYM4; GOLGA5; TMEM59; RUVBL1; C3orf63; HELZ; ZDHHC7; MAPK14; MCM3AP; RHOT1; HERC1; TMEM41B; CMTM6; GLOD4; UBR2; CUL5; LAMP1; ISCU; RNF111; VTI1B; PGK1; SFRS17A; SLC38A2; FOXJ3; SPTLC1; BTAF1; YIPF6; UBR4; NCOA4; NT5C2; SMCHD1; SNX1; MRFAP1L1; FRYL; UXT; GAK; PIGG; RBL2; KIDINS220; TRIM33; LRRC47; ACTR10; GGNBP2; MAPRE2; ROCK1; WDR37; PCGF3; KIAA0907; NUDT9; ZKSCAN1; CDIPT; UBE2Z; ZFAND6; CXorf45; C19orf56; SFRS5; RNASEN; C5orf15; PAPOLA; MDN1; C21orf66; VPS26A; TMEM1; RAB6A; ATRX; LRRC37A4; DENR; EIF4A2; ASF1A; PIP4K2B; NDUFB11; PPT1; ZNF451; GALNT7; DCN; PI4KAP1; VAMP7; MTCH1; RNMT; DCK; THOC2; CRYZL1; RAB7A; CUL4B; RAB6C; C21orf33; SLC9A6; METAP1; SPAG7; PNN; DCTN2; DERL2; SDF2; DDX3X; HNRPA3P1; GPATCH8; MSL-1; PDHA1; COX15; ANKRD49; SEC63; TTC3; ADAR; ZDHHC6; METT11D1; ZNF574; PARP6; LMBRD1; PHF8; IRAK1BP1; TLK1; PCNT; MPHOSPH9; HNRPH3; METTL3; SPEN; MAN1A2; TINAGL1; SLC22A5; ADCK2; PLAC4; NEBL; HIST1H2BN; BSPRY; ARL4A; ZNF528; DDR1; ABCC4; ATP1A3; TMEM63A; TAT; KMO; OR7E38P; MYOZ2; TCL6; COL1A1; PARD6A; C6orf60; SLC35F5; NCF1; MPP6; ANKRD55; VIPR2; RGS5; BCL9; EPAS1; UCK2; ERO1LB; CDC14B; E2F2; SEMA6D; RASIP1; KCTD20; MUC5AC; CD24; FADS3; TPR; PLGLB2; NR1D2; PHLDA2; ENTPD3; BCAS4; LRP4; PRL; CLEC2D; SPPL2B; AKAP12; SORT1; PCSK6; CPM; GJC1; RGS2; FLJ13769; SLFN12; MLPH; C6orf124; AASS; KCNJ2; BASP1; KCNJ3; RHOQ; SNTB2; IL8; CD84; FERMT1; CD48; PIP5K1B; SEMA5A; MDM4; PCSK5; CD72; PAX5; PLS3; FLJ20674; PDE4D; ATRNL1; GLDC; TRAK2; GPATCH2; RBBP5; KRAS; ADAM19; TRIM16; C1orf135; NFATC4; BMPR2; CELSR2; RAG2; ARHGAP8; TTC9; ST6GALNAC4; ITPKB; ZNF124; ARHGEF11; CSRP1; TCF3; SPAG6; SORBS2; MYT1L; MYL4; JAM2; PAWR; IGHM; GATM; SH3PXD2A; PPFIBP1; KCNJ16; TNK2; BIK; FAM152A; EPB41L2; VDR; TMSL8; EMP2; BACH2; NRGN; PRR5; CORO2B; PPFIA4; ARNTL2; SRGAP2; MAP3K1; PHACTR1; SH3BP4; EWSR1; F13A1; LTBP2; DOCK9; RHOBTB1; CASC1; TRIB2; TGFBR2; MAP1B; GPR176; PSAT1; PIGZ; ODZ4; ENDOD1; D4S234E; QRSL1; GNAZ; KCNA3; FNDC3B; ALDH1A1; GALNT14; SYT1; TMEM121; DOCK10; CALD1; RASAL1; ELOVL2; CCDC81; IL12RB2; IGSF3; APBB2; KCNMB3; ELL3; BLK; IRF4; KIAA1305; AOX1; ROR1; SAMD4A; LRMP; KANK1; ADARB1; DACT1; KCNJ12; FAT; FHOD3; SORBS1; SEMA4C; SLAMF1; GP5; HIP1R; NID2; PRKCZ; KIAA0802; SYNPO; PSEN2; SLC27A2; MERTK; PBX1 |
| Normal | MBD1 | <0.001 | 0.05189 | - | down | 1500 | 69 | PRDX3; NDUFA1; TRMT5; COPS3; COX7B; FRG1; HMGN2; RBBP7; PSMC5; UBE2A; ATPIF1; ATP5J; HMGN1; UCRC; FLII; SOD1; RCOR1; HMGN3; AHSA1; PECI; CCDC56; EIF4A3; RWDD1; PSMD2; SF3B5; SLC9A6; PHB; PSMA3; NDUFA8; MCTP2; GPHN; TP53TG3; CCR5; APOL6; GRIK2; CBLB; RASL10A; PDE8B; TOX3; KAL1; HIP1; AKAP12; FLRT3; EFR3B; PRKG2; ZNF239; CD84; ZNF192; CSHL1; CDH11; ANGPT2; REEP1; RAG2; LRRC8B; PCDH9; PCBP4; GLDC; FER1L3; SLC6A16; AGMAT; TXNRD3; MPPED2; HEY2; LHFPL2; CHST7; CHST2; STAP1; PTPRM; CENTG2 |
| Pseudodip | MBD1 | 0.005 | 0 | - | down | 2500 | 37 | C4orf41; SIRT1; RRAGA; NUP54; RAB5A; CD46; PSMC6; COPS4; LAMP2; TCEAL1; HNRPH2; NDUFB8; PIN4; BZRAP1; GRIK5; SSH3; ATP1A3; ANXA4; CTNS; E2F2; ERBB2; GPR176; SH2B2; PCDH9; CXCR3; DLGAP4; dJ222E13.2; HCRP1; MPO; SERHL2; PYCR1; COL9A3; TAP2; AEBP1; SLIT2; PALM; SFRS6 |
| Hyperdip>50 | MBD4 | <0.001 | 0 | - | down | 1000 | 257 | IGF2R; RNF40; EFTUD1; ANP32A; EFHA1; HDAC1; THADA; MCRS1; H1FX; C1orf160; RER1; TH1L; TCF12; MAP2K2; YLPM1; RMND5A; HDGF; TMEM183A; ADAM17; GOLGA1; NGLY1; C13orf23; UPF1; PPME1; IQCC; DLEU2L; PSCD2; MPHOSPH8; ZNF131; TFG; MZF1; PMS2L8; DKFZP564O0523; DDA1; MSL2L1; ARID4B; BTBD2; IPO9; RHOT2; SR140; IL7R; GTPBP8; PCM1; SFRS12; PMS2L3; MDM1; PMS2L1; RBBP4; ADNP; MCAM; PTCD3; MBD4; UBE2N; SNRP70; PMS2L11; ZNF364; CLEC16A; HIPK1; C5orf5; VPS13A; IQCB1; SNRPA; PSMF1; NOL12; FBXL14; ARIH2; RFWD3; HP1BP3; GNB1; WNK1; ORAI2; LONP1; MYO9B; DCTN1; GTF2F1; IRF3; ATR; EDC4; RAB5B; NKTR; BRD4; MRPS31; PPM1G; ANKHD1; HNRPUL1; HTATIP; NSUN6; RABL2A; C12orf47; TWF1; FAM89B; ANAPC5; PRPF38B; TRIM28; PIK3R1; C1orf77; ZMYND8; KLF12; NDUFA3; PIN1; LOC389168; NDUFS8; PHKG2; CAPN1; ING3; MLH1; HNRNPU; ZNF44; LOC23117; SLC25A36; POLR2J4; STAB1; S100A13; IFI6; SCARF1; BANK1; LILRB2; LDOC1; KIAA0427; CCND2; IER3; SLC16A2; ST7; SGSH; BACE1; NOS2A; DAPK1; ULK2; NPR1; TREML2; SPRY4; SCN1B; LARGE; NEDD9; RIN2; C17orf86; IFI30; OAS2; HLA-DMB; BCL2L2; SYNJ2BP; OR2A9P; ECM2; CYB561; XAF1; HERC6; SLC35E3; TIE1; HCP5; HLA-F; DUSP3; ARSD; NEO1; VIPR1; OAS1; EPHA7; ZNF516; BCAR3; ELK3; NPY; NDST1; PLEK; NCF2; ZNF467; HLA-E; KIAA1462; SERPINB6; PRSS7; F2RL1; PDK3; TBC1D9; IGFBP4; ACSL5; TNFSF13; RGL1; FGD1; WDFY3; STYK1; TNFRSF10B; CD86; CLEC4E; RPL10L; ERG; PPM1F; DDX60; TMEM165; KIAA1166; EXDL2; IL1B; PPAP2B; FLT3; GSTA4; IRF9; MAGT1; OCRL; IFIT5; FAM49A; SPIN2B; KBTBD11; IFIT3; IFI44L; MRC1; H2AFY2; PDE4B; NGFR; TNNI2; GNA11; RRAGB; CD1C; PLVAP; LGMN; CPD; C10orf10; IFIT1; CIDEB; SIGLEC15; ASB9; OGFRL1; ENOX2; RBM47; PLCH1; PET112L; TLR2; CSF3R; ALOX5; CELSR1; SH3BP2; PRKAR2B; CYTL1; ECHDC3; ARHGEF10; PIP3-E; CYB5A; EFNB1; SETD3; PDXK; MX1; SETBP1; PIGP; LOC57228; ALDH3B1; LTB4R; DHRS4; CEBPE; SH3BP5; TCEAL1; ITSN1; MYBPC2; ACOT9; SCML2; MS4A6A; TCEAL4; C10orf56; IL13RA1; MORC4; IL3RA; ZNF185 |
| E2A-PBX1 | MBD3 | <0.001 | 0 | - | down | 500 | 64 | UQCR; MED16; NCOR2; GPX4; CDC34; RPN2; TRMT1; NUCB1; VIPR1; SEMA5A; TLE1; KIF13A; LRRN2; FLJ20674; ALDH3A2; BMPR2; ARHGEF11; LOC90925; KLHL2; JAM2; IGHM; GATM; PPFIBP1; VDR; EMP2; BACH2; NRGN; KIAA0040; IL1B; CORO2B; PPFIA4; LTBP2; CASC1; MAP1B; SLC15A2; CSF2RB; ODZ4; FNDC3B; ALDH1A1; CALD1; RASAL1; ELOVL2; CCDC81; IGSF3; KCNMB3; ELL3; IRF4; AOX1; ROR1; SAMD4A; DACT1; KCNJ12; FAM3C; FHOD3; SLAMF1; GP5; HIP1R; NID2; PRKCZ; KIAA0802; SYNPO; PSEN2; MERTK; PBX1 |
| CCR | BAZ2B | <0.001 | 0 | - | down | 750 | 16 | LEPROT; MPG; ARL6IP5; GSTK1; STX12; MFSD1; FEZ2; MKI67; TCF4; HJURP; COL1A1; TOP2A; EIF4EBP2; MAGED1; HMG4L; DBN1 |
| E2A-PBX1 | SETDB1 | <0.001 | 0 | - | down | 1500 | 283 | KIAA0430; SNAP23; AKAP11; STX16; TTC31; SLC35E2; LETMD1; UBE4A; EXOC1; CSNK1D; UBE3B; FAM134C; TMEM189-UBE2V1; OS9; ZZZ3; UNC50; MBTPS1; IKBKB; ARL8B; WIPF1; GSTK1; HERC2P2; GMPR2; ARF3; TMEM131; ZMYM4; ATRN; WDR42A; JOSD3; R3HDM2; MBD3; C3orf63; ZDHHC7; CCBL2; RHOT1; CHD9; HERC1; POLRMT; TMEM41B; CMTM6; PIP5K3; MAP7D1; CUL5; RNF111; CCNT2; SCAMP1; PIGF; EZH1; ARHGAP1; FOXJ3; ZDHHC17; FNTA; SPTLC1; ST13; TXNDC14; UBR4; GPBP1L1; GTF3C3; OXA1L; NT5C2; RPL15; DOCK2; SNX1; ATP5D; TMEM39B; C1orf164; BICD2; PIGG; KIDINS220; TRIM33; LRRC47; MYT1; ACTR10; GGNBP2; VPS13C; KIAA0907; ZKSCAN1; CDIPT; UBE2Z; ZFAND6; SFRS5; RNASEN; PARP4; C5orf15; MDN1; KIAA0494; DENR; EIF4A2; C1orf63; DMTF1; TMEM87A; TAF1C; FOXK2; RNMT; CSNK2A2; RAB7A; RAB6C; DCTN2; C19orf60; ANAPC13; HNRPA3P1; GPATCH8; VKORC1; MSL-1; SCAMP3; GORASP2; METT11D1; PARP6; LASS2; NIF3L1; MPHOSPH9; TINAGL1; SLC22A5; ADCK2; DEPDC1; PLAC4; NEBL; HIST1H2BN; RALGPS2; BSPRY; ZNF528; DDR1; TAT; TPST1; KMO; MYOZ2; COL1A1; PARD6A; C6orf60; SLC35F5; ANKRD55; VIPR2; PXDN; CD9; ZNF669; ZNF257; RGS5; BAMBI; EPAS1; DEF8; C14orf132; CDC14B; E2F2; SEMA6D; RASIP1; KCTD20; MUC5AC; CD24; FADS3; TCF4; NR1D2; EGLN1; GINS4; ENTPD3; HIST1H4C; LRP4; PRL; VASH2; SPPL2B; AKAP12; SORT1; SPINT1; PCSK6; ITIH4; CPM; GJC1; MLPH; C6orf124; MYLK; AASS; TJP2; KCNJ2; RAI14; KCNJ3; SPA17; SNTB2; IL8; CD84; EXO1; FERMT1; VIPR1; SEMA5A; MDM4; PTTG3; PCSK5; LRIG1; TLE1; KIF13A; CKAP4; LRRN2; PLS3; FLJ20674; PDE4D; ALDH3A2; RHOB; ATRNL1; RHBDL2; CD59; TRAK2; ADAM19; TRIM16; C1orf135; NFATC4; BMPR2; TTC9; COBL; ARHGEF11; ZNF385D; KLHL2; SPAG6; SORBS2; MYT1L; MYL4; JAM2; PAWR; GATM; ARL4C; SH3PXD2A; PPFIBP1; KCNJ16; TNK2; VDR; TMSL8; EMP2; P4HA2; NRGN; IL1B; CORO2B; PPFIA4; ARNTL2; SRGAP2; MAP3K1; PHACTR1; SH3BP4; F13A1; LTBP2; DOCK9; CASC1; MAP1B; FGF9; GPR176; PSAT1; ODZ4; D4S234E; GNAZ; KCNA3; FNDC3B; ALDH1A1; GALNT14; TMEM121; CALD1; RASAL1; ELOVL2; CCDC81; IL12RB2; IGSF3; KCNMB3; ELL3; BLK; IRF4; AOX1; ROR1; SAMD4A; KANK1; ADARB1; DACT1; KCNJ12; FAT; NCAPD3; NP; FHOD3; SORBS1; SLAMF1; GP5; HIP1R; NID2; PRKCZ; KIAA0802; SYNPO; PSEN2; SLC27A2; MERTK; PBX1 |
| Normal | DNMT1 | <0.001 | 0 | - | down | 500 | 40 | NUCKS1; TRMT5; KIAA0101; TYMS; RBX1; HMGN2; CDC2; CCNB2; TUBA1C; MCM6; RPA3; RFC4; H2AFZ; NDUFA6; CBX5; TOP2A; ARL6IP1; CKS1B; RAN; SFRS2B; PSMD2; TUBB2C; TP53TG3; CCR5; GRIK2; PDE8B; TOX3; KAL1; EFR3B; PRKG2; ZNF239; CSHL1; ANGPT2; REEP1; FLT1; FER1L3; SLC6A16; AGMAT; PBX2; MPPED2 |
| Relapse | DNMT1 | <0.001 | 0 | - | down | 400 | 23 | SHCBP1; MCM10; TUBB; NCAPH; TOP2A; TPX2; SPAG5; ANP32E; KIF20A; CCNB2; CDC45L; BUB1; KIF11; PLK4; TIMELESS; MAD2L1; KIF2C; H2AFZ; CKS1B; ZWINT; RAD51; NPR3; FLJ13197 |
| Hyperdip>50 | DNMT3B | <0.001 | 0 | - | down | 1000 | 227 | C11orf49; NSUN5; SLC25A12; PIGB; EFHA1; HDAC1; GCLC; TMCO1; MTX2; NSUN5B; EXTL2; NSUN5C; YLPM1; AKAP2; BID; ARF4; NGLY1; C13orf23; MRPL16; ATIC; DLEU2L; LOC137886; PTBP2; TFG; PMS2L8; PALM2-AKAP2; XYLT1; MSL2L1; AGL; RHOBTB1; AUH; ARID4B; KIAA0182; DNAJB6; SR140; SLC25A46; PCM1; SFRS12; MDM1; SLC46A3; PMS2L1; RBBP4; SLC4A7; CD47; ADNP; RHOBTB3; C1orf181; PTCD3; CYFIP2; SHMT2; LZTFL1; FAM60A; GCC2; VPS13A; TMED5; IQCB1; SSX2IP; TGDS; KARS; FBXL14; PTP4A2; DYNC2LI1; ZC3H7A; Gcom1; C2orf25; TFRC; PWP1; DSTN; IPW; ATR; RRP15; ANKHD1; SCP2; NSUN6; FUBP1; TMEM38B; ZNF195; PATZ1; TWF1; ANAPC5; TFB2M; CEP110; PRPF38B; EPRS; UBA3; NME7; CTSC; MTIF2; ELOVL5; SLC25A13; SNX4; MTMR2; SNRPE; CBFB; ZBTB11; INSIG1; ITM2A; SH2B3; HIST1H2AI; BANK1; CCDC109B; GABBR1; MGP; DSE; LILRB2; CD9; LDOC1; STX7; CCND2; SLC16A2; TSC22D3; C14orf113; BACE1; NOS2A; NPR1; TREML2; HLA-B; SPRY4; SCN1B; LARGE; CENTD1; RHBDF2; HIST1H1C; RIN2; IFI30; MID1IP1; TFEB; HLA-DMB; DNASE1L1; CBR3; FHL1; CDKN1A; HLA-G; ECM2; XAF1; HLA-J; SLC35E3; HCP5; NAV1; HLA-F; TGIF1; KANK2; ARSD; XBP1; NEO1; VIPR1; OAS1; EPHA7; FUT7; IRF7; ELK3; NPY; SLC43A3; NDST1; KLF11; NCF2; ZNF467; PRSS7; TBC1D9; IGFBP4; TMEM50B; PDLIM1; IFI35; TRPM2; TNFSF13; RGL1; FGD1; WDFY3; FAM26B; CD86; IKBKG; ERG; PPM1F; PPAP2B; IRF9; KIAA0774; FAM49A; KBTBD11; IFIT3; C17orf60; MRC1; UNC93B1; PDE4B; TNNI2; GNA11; CDC42BPB; PLVAP; LGMN; IRF8; TLR1; C10orf10; IFIT1; CIDEB; SIGLEC15; ENOX2; RBM47; TLR2; ALOX5; CELSR1; DLG3; SH3BP2; CYBB; CYTL1; ECHDC3; NDE1; CBR1; ARHGEF10; PIP3-E; IL6R; EFNB1; IFNGR2; PDXK; MX1; SETBP1; LOC57228; ALDH3B1; LTB4R; CEBPE; SH3BP5; HDHD1A; ITSN1; MYBPC2; ACOT9; SCML2; ADAM8; IL13RA1; IL3RA |
| TEL-AML1 | DNMT3B | <0.001 | 0 | - | down | 1500 | 334 | IQGAP2; KIAA0564; FAM120A; ATP8A1; NIT2; DECR1; PTPN2; MPZL1; CABC1; SYNJ2; AMD1; SLC39A8; BRP44L; PXMP3; MRPL3; CTSC; ALDH9A1; JARID2; CASP4; PPIF; NEUROD1; LETM1; MTX2; SRPRB; SRP72; UCHL5; WAPAL; CD96; KLHL21; C15orf15; TPP2; MINPP1; MRE11A; EIF3M; HEATR3; TFB2M; WDR43; TRIB2; ITGA4; HADH; XYLT1; DHRS7; SMAP1; RFK; PTER; KTELC1; OSBPL3; ATIC; BRP44; STX6; VPS54; ASAH1; SLC1A4; GALC; WWOX; LRPPRC; HINT1; C21orf91; AACS; LRRFIP2; SLC7A1; POGK; MRPL9; NUP98; RPIA; POLR3E; HSPA14; ATP2C1; PRMT3; C11orf57; HK2; LGTN; LDHB; CLNS1A; TMCO1; RRP15; BZW2; CPSF6; CAPRIN2; MRPS30; GPSM2; AGL; FAM8A1; UBA5; ADA; TRMT11; PCM1; PLXNC1; CEPT1; PIK3CB; SHQ1; KIAA0182; H6PD; SNRPE; FAH; C2CD2; ITGAE; KIAA0125; NSUN5; USP20; THOC7; KIAA0776; IKBKAP; EPRS; C12orf11; LARP1; AMPD2; LANCL1; IDH1; WDR67; GCSH; TEX261; ANKRD27; PIGB; CDC2L6; ATP13A3; DLG3; SPP1; MYO1B; SCARF1; CD74; WSB2; KIAA0427; CSDA; CD97; SNX2; POU4F1; PRSS7; C11orf75; TAZ; LAPTM5; FAIM3; CDS2; CCND2; SOCS2; TCF4; HIST1H2AE; PQLC1; AGPAT2; PPP1R16B; EFNA4; NRXN3; HLA-DOA; IGKV1OR2-108; DOK3; IL24; HLA-DQB1; MAN1A1; HLA-DRA; MAP4K2; HIST1H2BK; EPHB4; SH3TC1; TP53TG3; RNF141; SMTN; BCL3; IGHA1; VCL; CEBPE; LARGE; PTPN12; HLA-DPB1; MAMLD1; HLA-DPA1; AKAP12; MDM2; SCML2; HLA-DRB1; APBB1IP; HSPB1; DUSP26; FAM127B; HIST1H2AJ; NPY; SERPINB9; HIST2H2AA3; CD52; GIMAP4; HIST1H3H; PFTK1; IFI16; HIST1H2BI; TMEM140; HLA-DRB5; CTGF; PLXNA3; HIST2H2BE; C9orf91; PTPRE; HIST1H2BE; WFS1; DGCR6; KIAA1539; TCL1B; CHD7; CTNNA1; TGFB1I1; MYLK; CLCN7; HIST1H2BG; HRK; HLA-DRB6; HIST1H2BH; SSX4; CCR1; MEF2C; CD40; KANK2; CEACAM1; EFEMP2; HLA-DMA; HLA-DMB; C1orf78; HIST1H2BD; AP1B1; EFNA1; CRIM1; PTPRG; CSF3R; tcag7.1314; MME; VPREB3; UTRN; LAIR1; SSX2; ST3GAL6; FADS3; ZNF274; MGC5370; RGL1; TSPAN14; LST1; PYHIN1; NPR1; MYO5C; DIP2C; FAM127A; ERG; HIST1H2BF; NEIL1; CD79A; C1orf38; HMGB3; SIGLEC15; JUP; FBXW7; CACNA1A; FGD1; JMJD2B; LILRA2; HS3ST1; PLCL2; MLXIP; LDOC1; VAMP5; GAB1; COL5A1; PXN; SLC27A3; DPEP1; FHIT; FAM50A; POU2AF1; FOXO1; GTF2IRD1; INSR; SLC35E3; PTK2; FAM65A; BTN2A2; TRIB1; LTB; SCHIP1; ITPR3; EGFL7; INTS3; C14orf132; ALOX5; NOL4; PRO2268; FCGRT; AK1; EPHA7; ISG20; BMP2; GNG7; ELK3; CKMT2; PSD3; LASS4; TBC1D8B; ITPR1; STK32B; GPR17; DUOX1; EDEM1; GABBR1; CD27; SDC1; KHDRBS3; NARFL; FERMT2; GRK5; SEMA6A; HLA-DOB; TBC1D9; H1F0; STAG3; SPTA1; CYB5R2; WDFY3; PID1; MAPK13; MDK; PTP4A3; RAG1; FUCA1; NRN1; TRPM4; SEMA3F; PDLIM7; TERF2; DRAM; PTPRK; NR3C2; SPANXA1; KCNK3; GBA3; ARHGAP29; TMEM16A; FBN2; TNS1; PCLO |
| Hyperdip>50 | HDAC1 | <0.001 | 0 | - | down | 1000 | 207 | EFTUD1; ANP32A; EFHA1; HDAC1; TMCO1; EIF3K; MAP2K2; YLPM1; PSME4; BID; NUP93; NDUFA7; ARF4; PSMA5; MRPL16; ATIC; UQCRH; IDH3B; RTCD1; FLJ14154; ZNF131; TFG; MRPL48; WDR61; MSL2L1; NHP2L1; HSPC152; DNAJB6; SR140; SLC25A46; PCM1; CIAPIN1; SFRS12; MDM1; RBBP4; CD47; C1orf181; COX7A2L; PTCD3; SNRP70; EXOC3; NGRN; ATP5L; FIBP; TAF9; TMED5; SNRPA; KARS; FBXL14; PTP4A2; ZC3H7A; CYCS; LONP1; C2orf25; PWP1; C19orf53; ATR; ADSL; ADAM10; IHPK2; PPM1G; UGP2; ANKHD1; SFI1; SCP2; ATF4; ETF1; TWF1; ANAPC5; NDUFA13; PRPF38B; C1orf77; NDUFB3; ATP5B; UBA3; DLG1; COX5A; HSBP1; SNX4; LOC440354; MTCH2; NDUFA3; PIN1; YARS; NDUFS8; CBFB; MLH1; ZBTB11; ARPP-19; ZDHHC4; GABARAPL2; INSIG1; GABBR1; MGP; CD9; LDOC1; KIAA0427; CCND2; IER3; C14orf113; BACE1; NOS2A; NPR1; TREML2; IFIH1; HLA-B; SPRY4; SCN1B; LARGE; HIST1H1C; NEDD9; RIN2; OAS2; HLA-DMB; HERC5; HLA-G; OR2A9P; ECM2; CYB561; XAF1; HLA-J; HERC6; SLC35E3; POU4F1; HCP5; NAV1; HLA-F; KANK2; ARSD; NEO1; VIPR1; SERPING1; OAS1; EPHA7; ZNF516; FUT7; IRF7; ELK3; NPY; NDST1; ZNF467; SERPINB6; PRSS7; F2RL1; PDK3; TBC1D9; PDLIM1; IFI35; RGL1; FGD1; WDFY3; CD86; CLEC4E; ERG; TMEM165; EXDL2; IL1B; PPAP2B; GSTA4; IRF9; PTPRE; KIAA0774; IFIT5; IFIT3; IFI44L; C17orf60; MRC1; H2AFY2; PDE4B; NGFR; TNNI2; GNA11; CDC42BPB; LGMN; TLR1; C10orf10; IFIT1; CIDEB; SIGLEC15; OGFRL1; RBM47; TLR2; DDEF1; CSF3R; ALOX5; CELSR1; DLG3; CYBB; CYTL1; ECHDC3; PIP3-E; IL6R; EFNB1; MX1; SETBP1; LOC57228; LTB4R; CEBPE; SH3BP5; ITSN1; MYBPC2; SCML2; MS4A6A; C10orf56; IL13RA1; IL3RA; ZNF185 |
| Normal | HDAC1 | <0.001 | 0 | - | down | 1000 | 56 | PSMA7; MRPL34; COX6C; BID; UCRC; TIMM8B; ENSA; MAPBPIP; NDUFA6; NDUFS6; RAN; COX6A1; MRP63; PSMD2; FKSG30; MRPL13; MDH1; NDUFS1; CD34; TP53TG3; CCR5; MYLIP; APOL6; GRIK2; RASL10A; PDE8B; TOX3; EGFL7; GNG11; FLRT3; CHD7; EFR3B; PRKG2; ABCB4; ZNF239; ZNF192; CSHL1; RXRA; CDH11; ANGPT2; ITGA6; REEP1; FLT1; PCBP4; FER1L3; AGMAT; LGR5; PBX2; TXNRD3; MPPED2; HEY2; LHFPL2; CHST7; GFOD1; PTPRM; CENTG2 |
| Normal | HDAC2 | <0.001 | 0 | - | down | 500 | 44 | PRDX3; NDUFA1; TRMT5; COPS3; COX7B; FRG1; LAGE3; HMGN2; SMS; RBBP7; PSMC5; UBE2A; TBC1D25; ATP5J; HMGN1; SOD1; HMGN3; PECI; NDUFB8; CCDC56; EIF4A3; H2AFZ; C4orf27; RWDD1; SF3B5; PGAM1; MRPL13; PSMA3; NDUFA8; TP53TG3; CCR5; APOL6; GRIK2; CBLB; RASL10A; FLRT3; EFR3B; PRKG2; ZNF192; CSHL1; REEP1; PCDH9; FER1L3; AGMAT |
| Pseudodip | HDAC2 | <0.001 | 0 | - | down | 1500 | 27 | C4orf41; SIRT1; NUP54; RAB5A; PSMC6; COPS4; TCEAL1; HNRPH2; NDUFB8; PIN4; BZRAP1; GRIK5; SSH3; GLT25D1; HRK; ERBB2; GPR176; SH2B2; PCDH9; dJ222E13.2; HCRP1; SERHL2; TAP2; ATM; SLIT2; PALM; SFRS6 |
| Hyperdip>50 | HDAC4 | <0.001 | 0 | - | down | 500 | 68 | C11orf49; EFTUD1; NSUN5; BTG3; HDAC1; ODC1; TMCO1; MTX2; PKIA; EXTL2; C9orf16; FLJ11506; AKAP2; TMEM183A; C13orf23; MRPL16; ATIC; C16orf24; LOC137886; PTBP2; MINPP1; FLJ14154; ITPKB; TFG; MRPL48; PALM2-AKAP2; MSL2L1; AGL; RHOBTB1; PIK3R3; KIAA0182; DNAJB6; SLC25A46; GTPBP8; SLC4A7; NFATC1; CSRP1; CYFIP2; IFI35; ACSL5; TRPM2; RGL1; FGD1; TNFRSF10B; ERG; PPM1F; PTPRE; FAM49A; IFIT3; C17orf60; MRC1; PDE4B; TNNI2; GNA11; LGMN; IRF8; TLR1; CIDEB; DDEF1; ALOX5; CYBB; CYTL1; ECHDC3; EFNB1; LOC57228; LTB4R; SCML2; IL3RA |
| Normal | HDAC4 | <0.001 | 0 | - | down | 1500 | 86 | CAPG; NUCKS1; NDUFS5; PSMA7; APIP; MRPL34; PITPNC1; COX6C; TOR3A; MAGEF1; NUBP2; RNPEP; NANS; SS18L2; PSMB3; SCCPDH; BID; SEC24D; ARL3; UCRC; SYNJ2; TIMM8B; ARPC5; PTTG1; FH; MAPBPIP; CCNB2; TUBA1C; RPA3; HN1; NDUFB8; CBX1; ATP5G1; H2AFZ; NDUFA6; NDUFS6; GPI; COX6A1; SFRS2B; INTS7; MRP63; PSMD2; SF3B5; TUBB2C; PGAM1; FKSG30; MRPL13; GRAMD4; NCAPD3; MDH1; NDUFS1; CD34; TP53TG3; CCR5; MYLIP; APOL6; MXI1; DLGAP4; RASL10A; PDE8B; EGFL7; AKAP12; GNG11; CHD7; MEF2A; ABCB4; ZNF187; PTGDR; APOBEC3G; RXRA; TLE4; ITGA6; FLT1; CASK; PCBP4; AP1B1; FER1L3; CAPN3; LGR5; PBX2; HEY2; LHFPL2; GFOD1; STAP1; PTPRM; CENTG2 |
| TEL-AML1 | HDAC4 | <0.001 | 0.08862 | - | down | 1000 | 237 | PRPS2; SMYD2; PTGER4; UCK2; CCDC86; LPXN; DPH4; NUP210; DECR1; PTPN2; CABC1; ZNF593; ACOT7; SYNJ2; CDKN2D; BRP44L; PXMP3; MRPL3; CTSC; ALDH9A1; CIB1; JARID2; PTPN6; ACAT2; NFATC1; PPIF; FDX1; C9orf16; LETM1; MTX2; C8orf33; GRAMD4; UCHL5; WAPAL; MRLC2; CD96; TPP2; CSRP1; MINPP1; SRI; TFB2M; TRIB2; SF3B5; MRPS34; RFK; RAP2B; LSM4; ATIC; BRP44; STRAP; ADARB1; ADAM10; ARL3; CDK2AP2; SLC1A4; AACS; POGK; MRPL9; APEH; KATNA1; RPIA; HSPA14; SPON2; RABIF; LDHB; CLNS1A; TMCO1; CPSF6; CAPRIN2; FH; HLA-DOA; IGKV1OR2-108; DOK3; HLA-DQB1; MAN1A1; HLA-DRA; HIST1H2BK; EPHB4; SH3TC1; TP53TG3; TNFRSF10B; AHDC1; RFTN1; LARGE; PTPN12; SLC12A6; C1orf165; HLA-DPB1; MAMLD1; HLA-DPA1; SCMH1; MDM2; SCML2; HLA-DRB1; DUSP26; HIST1H2AJ; NPY; SERPINB9; HIST2H2AA3; RDX; CD52; GIMAP4; PFTK1; IFI16; HIST1H2BI; TMEM140; HLA-DRB5; CTGF; HIST2H2BE; PTPRE; HIST1H2BE; WFS1; DGCR6; KIAA1539; TXNIP; CHD7; MYLK; APP; CLCN7; HIST1H2BG; HRK; HLA-DRB6; HIST1H2BH; SSX4; CCR1; MEF2C; TMEM2; CD40; LYL1; KANK2; EFEMP2; HLA-DMA; HLA-DMB; ZCCHC14; AP1B1; EFNA1; CRIM1; PTPRG; CSF3R; ZMIZ1; tcag7.1314; MME; VPREB3; HHEX; CLCC1; UBA7; UTRN; LAIR1; SSX2; ST3GAL6; PLCXD1; ZNF274; MGC5370; RGL1; TSPAN14; CD200; LST1; NPR1; MYO5C; ERG; HIST1H2BF; REL; CD79A; C1orf38; ZNF117; ENTPD4; SIGLEC15; MAPK8IP3; IL6ST; JUP; FBXW7; FGD1; LILRA2; HS3ST1; OCA2; MLXIP; LDOC1; GAB1; COL5A1; SLC27A3; DPEP1; FHIT; POU2AF1; FOXO1; INSR; SLC35E3; PTK2; FAM65A; BTN2A2; TRIB1; SCHIP1; ARHGAP24; EGFL7; INTS3; DNMBP; ALOX5; NOL4; PRO2268; AK1; EPHA7; ISG20; BMP2; GNG7; ELK3; CKMT2; PSD3; TBC1D8B; ITPR1; STK32B; DSC2; EDEM1; GABBR1; MSR1; SMAD1; WASF2; SEMA6A; SCARB1; HLA-DOB; TBC1D9; STAG3; GNG11; SPTA1; CYB5R2; WDFY3; MDK; C13orf18; PTP4A3; RAG1; FUCA1; SEMA3F; TERF2; DRAM; NR3C2; KCNK3; HAP1; TNS1; PCLO |
| BCR-ABL | HDAC9 | 0.009 | 0 | - | down | 1500 | 160 | FRAT2; GLUL; DGKD; WASF1; RABL4; RCOR1; ORC5L; GCNT1; CLASP2; ZEB2; RB1; C1GALT1; KTELC1; BLK; ODC1; SLC4A7; HK2; APBB2; PPFIA1; PTPN2; P2RX5; FLI1; SNX4; LOC57228; CLSTN1; FHIT; PRKCH; SQRDL; PLXND1; KHDRBS3; TCF7L2; EMP2; P2RY5; CTDSP2; MDM2; GIPC1; TMEM134; DDR1; PDXK; MGC29506; NEDD4; PTPLA; BRF1; RASL10A; HIF1A; CCDC88C; ETV6; MME; NEDD9; CRIP2; HDAC7; HLA-DPB1; SH3BP5; ITPR1; EMILIN1; OPTN; PRKCA; BBC3; TXNIP; C13orf15; LGMN; ST3GAL5; LAIR2; HLA-DQB1; EDEM1; LST1; YES1; EGLN1; HBS1L; HSPB1; PVRIG; C11orf75; PXN; IFITM1; MYLK; LARGE; CD52; MED9; ILVBL; SERHL2; PECAM1; A2M; IFITM2; VAMP5; MAP3K11; SYNE2; PTPRD; EFNA1; FXYD6; ID3; RPL23AP13; GPR56; ZNF467; DNTT; TncRNA; HLA-DQA1; XBP1; GAB1; DUSP26; RAB13; CD34; CTNNBIP1; AMBRA1; SERPINB9; RAI14; MRC1; STARD3; LAIR1; MYO5C; PTPN18; RAB11A; NAV1; TNFRSF14; SV2A; SCHIP1; FBXW7; FYN; FZD6; MYO1B; BST1; IFITM3; FARP1; PDE9A; PHF15; ITGA6; CD99; CENTD1; C6orf32; CD27; NRXN3; BMPR1B; GIMAP4; PALM; FLJ20489; ITGA5; CCND2; OSBPL10; GIMAP6; FSCN1; RAPGEF3; COLQ; ARHGEF17; MGC4655; GADD45A; GIMAP5; SEMA6A; ASB13; FAM129A; PRX; TUBA4A; COL6A3; NPDC1; CDS2; CASP10; CNN3; ECM1; SLC2A5; LOC26010; OLFML2A; CTDSPL |
| E2A-PBX1 | HDAC6 | <0.001 | 0 | - | down | 750 | 125 | MSN; ARHGAP4; OGT; WDR44; TMED10; MORC3; EXOC1; CSNK1D; TMEM30A; KIAA1109; ZFP106; FAM134C; TUBGCP2; CLINT1; ATP6AP2; UBE2D3; TMEM164; LAMP2; PAFAH1B1; RPS6KA3; RP6-213H19.1; SSR4; WSB1; SERINC1; TM9SF1; ARAF; WDR42A; RUVBL1; ZDHHC7; GCN5L2; MCM3AP; IL10RB; GDI1; KIAA0831; GLOD4; WDR45; ASMTL; UBR2; VTI1B; PGK1; SFRS17A; MECP2; BTAF1; RGS5; CDC14B; SEMA6D; MUC5AC; FADS3; ENTPD3; HIST1H4C; SPPL2B; AKAP12; CPM; FLJ13769; MLPH; C6orf124; MYLK; KCNJ2; CD84; FERMT1; CD48; PIP5K1B; SEMA5A; MDM4; PCSK5; PLS3; ATRNL1; GLDC; TRAK2; GPATCH2; TRIM16; C1orf135; RAG2; ST6GALNAC4; ZNF124; ARHGEF11; CSRP1; TCF3; MYL4; JAM2; PAWR; GATM; PPFIBP1; KCNJ16; BIK; EPB41L2; VDR; EMP2; CORO2B; ARNTL2; MAP3K1; EWSR1; LTBP2; CASC1; MAP1B; GPR176; ODZ4; QRSL1; TSSC1; FNDC3B; ALDH1A1; SYT1; CALD1; RASAL1; ELOVL2; IL12RB2; APBB2; KCNMB3; IRF4; AOX1; ROR1; DACT1; KCNJ12; FAT; SORBS1; SEMA4C; SLAMF1; GP5; HIP1R; NID2; PRKCZ; KIAA0802; SYNPO; PSEN2; PBX1 |
| Normal | HDAC6 | <0.001 | 0 | - | down | 1500 | 68 | HPRT1; SLC10A3; NDUFA1; HSDL2; COX7B; LAGE3; SMS; RBBP7; UCHL5IP; PSMC5; UBE2A; TBC1D25; ETHE1; ATP5J; PGD; HMGN1; ARMCX1; FLII; SOD1; BCAP31; C21orf59; RCOR1; HMGN3; AHSA1; SLC25A5; LRPAP1; EIF4A3; EBP; SLC9A6; PHB; AMPD3; HSPA5; MCTP2; GPHN; TP53TG3; APOL6; GRIK2; CBLB; RASL10A; TOX3; KAL1; AKAP12; FLRT3; EFR3B; PRKG2; ZNF239; PTGDR; CD84; ZNF192; CSHL1; ANGPT2; REEP1; GRAMD1B; FLT1; RAG2; LRRC8B; PCDH9; GLDC; FER1L3; AGMAT; PHYH; TXNRD3; MPPED2; HEY2; LHFPL2; STAP1; PTPRM; CENTG2 |
| Pseudodip | HDAC6 | <0.001 | 0.06117 | - | up | 1000 | 27 | SPIN2B; GSPT2; PIGA; ALG13; BACH1; RP2; C4orf41; SIRT1; RRAGA; NUP54; TMEM164; PSMC6; COPS4; LAMP2; TCEAL1; HNRPH2; PIN4; BZRAP1; GRIK5; ANXA4; ERBB2; GPR176; PCDH9; dJ222E13.2; SERHL2; SLIT2; SFRS6 |
| Normal | HDAC3 | 0.006 | 0 | - | down | 2500 | 100 | HPRT1; LMAN2; TRMT5; NDUFB2; COPS3; COX6C; LAGE3; HMGN2; PSMC5; PSMB3; ATPIF1; TBC1D25; ATP5J; PGD; HMGN1; COPS6; UCRC; FLII; BCAP31; ENSA; RCOR1; HMGN3; AHSA1; PECI; NDUFB8; CCDC56; EIF4A3; NDUFS6; GPI; ARL6IP1; RAN; COX6A1; SFRS2B; MRP63; RWDD1; PSMD2; SF3B5; PGAM1; U2AF1; PSMD4; IQGAP1; MDH1; NDUFS1; PSMA3; HSPA5; NDUFA8; MCTP2; CD34; GPHN; RIMS3; TP53TG3; CCR5; APOL6; GRIK2; MXI1; CBLB; DLGAP4; RASL10A; PDE8B; TOX3; EGFL7; KAL1; HIP1; AKAP12; FLRT3; EFR3B; PRKG2; ABCB4; ZNF239; PTGDR; CD84; ZNF192; CSHL1; RXRA; CDH11; ANGPT2; LIG4; REEP1; RP1-21O18.1; FLT1; RAG2; LRRC8B; PCDH9; PCBP4; GLDC; RHBDL2; FER1L3; SLC6A16; CAPN3; AGMAT; LGR5; PBX2; TXNRD3; MPPED2; HEY2; LHFPL2; CHST7; STAP1; PTPRM; CENTG2 |
| MLL | HDAC7 | <0.001 | 0 | - | down | 750 | 104 | MGC29506; CD2AP; ATP2B4; PRKCH; TNFRSF14; C11orf75; GIMAP4; FAM134B; YES1; RHOF; RYK; NDFIP1; PBXIP1; EDEM1; FAM117A; ZNF512B; TSPAN7; GPR56; SLC5A3; ABLIM1; CEP68; LTB; GIMAP6; CCDC92; POGZ; PPP1R16B; FGFR1; PLCG1; C6orf32; GIMAP5; LST1; BTN3A3; CANT1; BEX4; STAT5B; NF1; ENOSF1; ITPR1; ICAM3; PVRIG; SERINC5; HDAC7; SPTAN1; MKL2; KIAA0317; SETD5; FAM102A; FYN; FUT8; ZMAT3; SERPINB8; WIT1; SPIB; NIP7; SMURF1; HDAC9; SERPINI2; NR5A2; PCDHGA11; RHOQ; EHD4; XYLT1; DGKD; B3GALNT1; MPEG1; UCK2; TBCC; RGS16; FOSL2; FXN; MARCH3; PUS7; SPG20; STARD13; CLEC2D; VNN1; VLDLR; ADK; PTGES3; BCAS4; STS; PRKCE; ZEB2; IGFBP7; PTCH1; THSD7A; CEBPA; CD72; C11orf24; CD44; GREM1; ADCY9; TUBB6; ACSL1; GPM6B; SCPEP1; RHOBTB3; MPZL1; MAP7; ABHD4; ATP8B4; MAP3K5; CCNA1; C20orf103 |
| Hyperdip>50 | SMARCC1 | <0.001 | 0 | - | down | 2000 | 529 | TAF10; RNF40; EFTUD1; ANP32A; TOMM22; SLC25A3; ITPA; EFHA1; HDAC1; THADA; FUS; MCRS1; PGLS; PAIP1; C1orf160; C9orf16; RER1; EIF3K; TH1L; GDE1; MAP2K2; YLPM1; PSME4; RMND5A; PRKCSH; BID; NUP93; TMEM183A; NDUFA7; ARF4; NGLY1; SF3A1; C13orf23; PSMA5; UPF1; IQCC; MRPL16; ATIC; TXN2; PSCD2; WDR77; MRPS27; UQCRH; HEATR1; IDH3B; RTCD1; ZNF131; MAT2A; TFG; MRPL48; EIF4ENIF1; WDR61; PMS2L8; MSL2L1; NHP2L1; C20orf30; HSPC152; BRD9; MASP1; SR140; NCBP2; AP3B1; SLC25A46; PCM1; CIAPIN1; SFRS12; PMS2L3; MDM1; ARPC1A; OGFOD1; PMS2L1; RBBP4; CD47; ADNP; MCAM; C1orf181; UCHL3; COX7A2L; PTCD3; STUB1; SHMT2; ACP1; FAM60A; CLEC16A; ATP5G2; NGRN; FIBP; TAF9; C12orf10; EIF3D; SNRPA; ARID1A; PSMF1; KARS; NOL12; FBXL14; PTP4A2; ARIH2; RFWD3; ZC3H7A; GNB1; Gcom1; CYCS; SNRPF; EIF3EIP; LONP1; C2orf25; MYO9B; PWP1; DCTN1; GTF2F1; C19orf53; ATR; ADSL; CALU; INPP4A; CSNK2A1; TEX10; IHPK2; PPM1G; ANKHD1; HNRPUL1; HTATIP; SCP2; ATF4; FUBP1; ETF1; RABL2A; ZNF195; C12orf47; PATZ1; FAM89B; ANAPC5; NDUFA13; TRIM28; CYC1; EPRS; C1orf77; ATP5B; PNKP; UBA3; DLG1; COX5A; PPP6C; ERGIC3; PAFAH1B3; SUPT5H; DDX41; HSBP1; SNX4; CAPRIN1; MTCH2; RPL24; EIF3J; NDUFA3; DNAJA3; PIN1; USP21; YARS; OBFC2B; SNRPE; NDUFS8; PHKG2; CBFB; MLH1; MPV17; HNRNPU; ZBTB11; ZDHHC4; GABARAPL2; PCBP2; ASXL1; DRAP1; SMPD4; BZW1L1; RNASEH1; PRPF19; TERF2IP; VPS13B; HNRPK; SMARCC1; EIF3I; SKP2; EIF4G1; ACADM; EXOSC2; SFRS9; APEH; SERBP1; TUFM; ATP6V1B2; ATP5SL; UBAP2L; DDX39; POLR3E; YEATS2; HIATL1; HMOX2; CHD4; GRSF1; PSMC3; ATP1B3; PRCC; RBM8A; CSDE1; C5orf13; GTF3C3; GPR89B; HYOU1; LEPROTL1; GTF3C2; CIAO1; SSSCA1; PTDSS1; ZNHIT1; HARS; WDR70; ATP5D; ATP5G3; KHSRP; GTF2I; ATP6V0A2; NDUFA10; POLR2J; HNRNPA1; SF3B1; GSPT1; TIA1; RANBP1; MAP4; MRPL3; FXR1; DHPS; CCT5; FIS1; C20orf11; EIF3M; COPZ1; COX4NB; SLMO2; DCTN6; FAM120A; OAZ1; CHMP1A; EIF2B5; COX4I1; AP3D1; SEP15; C3orf60; RBM39; ST13; CPSF6; BCL7B; ACO2; HMGB1; USP7; TUBB3; C20orf24; ASCC2; EIF2B1; THOC5; SKIV2L2; YKT6; UFC1; FBL; NCBP1; ACTL6A; TFPT; MAMLD1; PHF1; SERPINB9; WFS1; NR4A2; SLC6A16; IFI27; SIRPA; VNN1; OPTN; HIST1H2BH; C14orf139; STK32B; MYO1B; 3.8-1; BTK; HIST1H2AJ; NUAK2; TSPAN13; CEACAM6; RAI14; HIST1H2BI; IGKV1OR2-108; HERC3; LAT2; ST3GAL6; P4HA2; GALNAC4S-6ST; TRIM38; ADM; CNN2; PRO2268; EFNA1; HIST1H2AM; NR4A1; LHFP; HLA-DRB5; SCNN1A; KLF9; DGCR6; C1orf38; HLA-DPA1; TCL1A; CLOCK; TCF4; HLA-DRB1; LMO2; SLC27A3; CTBP2; CD69; CAST; HIST1H2BK; LXN; LILRB3; KIF13A; LILRB1; BLNK; EMP1; VPREB1; TLR7; EIF2AK2; MGC5370; FXYD6; HLA-DRB6; SCHIP1; P2RY14; EPHB3; MAP3K8; CCR6; GPR171; HLA-DQB1; KLHL2; OFD1; LY86; CRAT; CENTA2; PTK2; PRKAG2; ECM1; ENTPD1; C14orf132; HSPA1B; TBKBP1; TNFRSF1B; STAB1; S100A13; SCARF1; HIST1H2AI; BANK1; GABBR1; MGP; DSE; LILRB2; CD9; LDOC1; KIAA0427; STX7; HSPA2; CCND2; IER3; SLC16A2; ST7; SGSH; TSC22D3; C14orf113; BACE1; NOS2A; ULK2; NPR1; TREML2; HLA-B; SPRY4; SCN1B; LARGE; RHBDF2; HIST1H1C; NEDD9; ZRANB1; RIN2; IFI30; KIAA0513; TFEB; KIF1B; HLA-DMB; BCL2L2; FHL1; OR2A9P; ECM2; GPR56; CYB561; XAF1; HLA-J; HERC6; SLC35E3; POU4F1; TIE1; RCAN1; HCP5; NAV1; HLA-F; DUSP3; KANK2; NEO1; VIPR1; OAS1; EPHA7; ZNF516; FUT7; IRF7; ELK3; NPY; NDST1; KLF11; NCF2; ZNF467; KIAA1462; SERPINB6; PRSS7; F2RL1; PDK3; TBC1D9; DYNLT3; IGFBP4; PDLIM1; HLA-A; IFI35; ACSL5; TRPM2; TNFSF13; RGL1; FGD1; WDFY3; STYK1; CD86; CLEC4E; CCL3; ERG; PPM1F; TMEM165; ERCC6L; EXDL2; LGALS3BP; IL1B; PPAP2B; FLT3; GSTA4; IRF9; PTPRE; MGAT4A; MAGT1; OCRL; KIAA0774; FAM49A; PECAM1; KBTBD11; IFIT3; IFI44L; C17orf60; MRC1; UNC93B1; H2AFY2; PDE4B; NGFR; SAT1; TNNI2; GNA11; RRAGB; CD1C; CDC42BPB; PLVAP; LGMN; IRF8; TLR1; C10orf10; IFIT1; CIDEB; SIGLEC15; ASB9; OGFRL1; ENOX2; RBM47; PLCH1; TLR2; CSF3R; ALOX5; CELSR1; SH3BP2; PRKAR2B; CYBB; CYTL1; ECHDC3; NDE1; CBR1; ARHGEF10; ZFYVE26; PIP3-E; IL6R; EFNB1; IFNGR2; PDXK; MX1; SETBP1; LOC57228; ALDH3B1; LTB4R; CEBPE; SH3BP5; HDHD1A; ITSN1; MYBPC2; ACOT9; GPRASP1; SCML2; MS4A6A; ADAM8; SCML1; C10orf56; IL13RA1; MORC4; IL3RA; ZNF185 |
| Normal | SMARCD2 | <0.001 | 0 | - | down | 1500 | 69 | ARPC1B; TRSPAP1; LMAN2; MRPL34; COPS3; ARPC3; C16orf33; PSMC5; MAGOH; PSMB3; DCI; FLII; PTTG1; FH; MAPBPIP; AHSA1; CTNNAL1; RFC4; PDIA6; EIF4A3; CBX5; TWF2; ARL6IP1; RAN; CLTB; GTF2E2; COX6A1; SFRS2B; PSMD2; TUBB2C; U2AF1; PSMD4; NDUFS1; HSPC157; CD34; GATA3; GPHN; TP53TG3; CCR5; APOL6; GRIK2; TOX3; KAL1; FLRT3; EFR3B; PRKG2; ZNF239; CD84; ZNF192; CSHL1; RXRA; ITGA6; REEP1; RP1-21O18.1; FLT1; CASK; PRUNE2; RGS9; FER1L3; SLC6A16; AGMAT; PHYH; TXNRD3; MPPED2; LHFPL2; CHST7; GFOD1; CHST2; PTPRM |
| CCR | SMARCA2 | <0.001 | 0.08084 | - | down | 500 | 19 | LEPROT; MLC1; ARL6IP5; GSTK1; WIPI1; MAP3K5; ADCY9; MFSD1; HK2; COMT; FEZ2; CDC25B; NUDT11; TCEA2; EIF4EBP2; MAGED1; SALL2; ZNF253; DBN1 |
| Hyperdip>50 | SMARCD1 | 0.008 | 0 | - | down | 2500 | 570 | TAF10; C11orf2; RNF40; C11orf49; EFTUD1; BTG3; ANP32A; SLC25A3; ITPA; EFHA1; HDAC1; LOC51035; MCRS1; TMCO1; PAIP1; C1orf160; EXTL2; C9orf16; EIF3K; FLJ11506; TH1L; MAP2K2; YLPM1; PSME4; RMND5A; HDGF; NUP93; TMEM183A; TTC15; NDUFA7; PCGF1; ADAM17; ARF4; NGLY1; PSMA5; MRPS34; PPME1; MRPL16; ATIC; USP4; PSCD2; WDR77; MRPS27; UQCRH; PTBP2; IDH3B; RTCD1; FLJ14154; ZNF131; TFG; MRPL48; WDR61; CTPS; MSL2L1; NHP2L1; REPIN1; HSPC152; BRD9; MASP1; SR140; NCBP2; AP3B1; SLC25A46; UCKL1; PCM1; CIAPIN1; SFRS12; MDM1; RBBP4; SF3A2; SLC4A7; CD47; C1orf181; COX7A2L; PTCD3; STUB1; SNRP70; EXOC3; FAM60A; CLEC16A; NGRN; PTPRA; FIBP; TAF9; TMED5; C12orf10; EIF3D; SNRPA; ARID1A; CHP; PSMF1; KARS; FBXL14; PTP4A2; ARIH2; C16orf57; ZC3H7A; CYCS; SNRPF; EIF3EIP; LONP1; C2orf25; PWP1; DCTN1; C19orf53; ATR; ADSL; EDC4; INPP4A; ZNF580; IHPK2; TMEM87A; ARFIP2; PPM1G; ANKHD1; SFI1; HTATIP; SCP2; ATF4; FUBP1; ETF1; PATZ1; TWF1; FAM89B; ANAPC5; NDUFA13; NUP133; TFB2M; TRIM28; EPRS; C1orf77; ATP5B; UBA3; DLG1; COX5A; PPP6C; MTIF2; DDX41; HSBP1; SNX4; LOC440354; MTCH2; EIF3J; NDUFA3; PIN1; YTHDC2; YARS; OBFC2B; SNRPE; NDUFS8; CBFB; SPTAN1; MLH1; ARPP-19; ZDHHC4; GABARAPL2; MARS; PMS1; GOLGB1; FADS1; SPSB3; DRAP1; SMPD4; WDR59; BZW1L1; PRPF19; ZFP64; TERF2IP; GNPAT; VPS13B; HNRPK; SMARCC1; EIF3I; SKP2; ACADM; SFRS9; APEH; TUFM; ATP5SL; UBAP2L; DDX39; POLR3E; TTC1; YEATS2; HIATL1; HMOX2; CHD4; GRSF1; NAT11; ATP1B3; PRCC; RBM8A; CSDE1; GTF3C3; GPR89B; HYOU1; LEPROTL1; GTF3C2; ABCD3; PTDSS1; SSBP3; ZNHIT1; HARS; DYNLL1; ATP5G3; KHSRP; GTF2I; NDUFA10; HNRNPA1; SF3B1; COPG; GSPT1; HAX1; RANBP1; UBA5; MAP4; MRPL3; FXR1; DHPS; CCT5; HRAS; C20orf11; EIF3M; COPZ1; PDE6D; SIAH2; COX4NB; SLMO2; DCTN6; FAM120A; POMP; HSPA4; MFN1; CHMP1A; EIF2B5; COX4I1; VRK3; AP3D1; VPS13C; SEP15; C5orf28; CDC16; FNBP4; NUCB2; NIPA2; RBM39; ST13; CPSF6; BCL7B; ACO2; USP7; HSPBAP1; TUBB3; C20orf24; ASCC2; EIF2B1; THOC5; YKT6; ZC3H13; UFC1; FBL; LYRM1; ACTL6A; TFPT; SMTN; MAMLD1; SERPINB9; WFS1; SLC6A16; IFI27; SIRPA; SLITRK5; MYO1F; VNN1; OPTN; HIST1H2BH; C14orf139; LRP3; STK32B; MYO1B; 3.8-1; BTK; HIST1H2AJ; CYB5R2; NUAK2; TSPAN13; CEACAM6; RAI14; HIST1H2BI; ZNF443; IGKV1OR2-108; HERC3; NUDT3; LAT2; ST3GAL6; P4HA2; HHEX; GALNAC4S-6ST; TRIM38; ADM; CNN2; PRO2268; EFNA1; HIST1H2AM; SYNGR1; DNTT; HLA-DRB5; SCNN1A; DGCR6; C14orf106; TNFSF10; LY6E; COL18A1; C1orf38; HLA-DPA1; MYLIP; TCL1A; CLOCK; TCF4; HLA-DRB1; TSPAN14; EPHB4; SLC27A3; RNF141; ALOX5AP; HIST1H2BK; LILRB3; KIF13A; LILRB1; BLNK; EMP1; VPREB1; TLR7; MANBA; EIF2AK2; MGC5370; FXYD6; HLA-DRB6; ZNF165; SCHIP1; P2RY14; EPHB3; ABCD1; CCR6; GPR171; HLA-DQB1; KLHL2; LY86; CRAT; PTK2; PRKAG2; MED9; KIAA0368; ECM1; ENTPD1; C14orf132; GPX1; MBIP; HSPA1B; GPR132; USP18; HRSP12; TNFRSF1B; IFI44; STAB1; SPTLC2; FLJ43663; S100A13; IFI6; SCARF1; SH2B3; HIST1H2AI; BANK1; GABBR1; MGP; DSE; LILRB2; CD9; LDOC1; KIAA0427; STX7; TWSG1; HSPA2; CCND2; IER3; SLC16A2; ST7; SGSH; C14orf113; BACE1; MX2; NOS2A; DAPK1; ULK2; NPR1; TREML2; IFIH1; HLA-B; SPRY4; SCN1B; LARGE; RHBDF2; HIST1H1C; NEDD9; RIN2; IFI30; KIAA0513; PLEKHA1; OAS2; HLA-DMB; HERC5; CBR3; FHL1; CDKN1A; HLA-G; OR2A9P; ECM2; CYB561; XAF1; HLA-J; HERC6; SLC35E3; POU4F1; TIE1; HCP5; NAV1; HLA-F; DUSP3; TGIF1; KANK2; ARSD; XBP1; NEO1; BRWD1; VIPR1; SERPING1; OAS1; EPHA7; ZNF516; FLNB; FUT7; BCAR3; IRF7; ELK3; NPY; FRMD4B; NDST1; PLEK; NCF2; ZNF467; KIAA1462; SERPINB6; PRSS7; F2RL1; PDK3; TBC1D9; LSP1; DYNLT3; IGFBP4; TMEM50B; PDLIM1; HLA-A; IFI35; ACSL5; TRPM2; TNFSF13; RGL1; FGD1; WDFY3; STYK1; TNFRSF10B; NFE2; ETS2; CD86; CLEC4E; GALNT3; CCL3; IL17RA; ERG; PPM1F; DDX60; TMEM165; ERCC6L; EXDL2; LGALS3BP; IL1B; PPAP2B; TAF9B; FLT3; GSTA4; IRF9; PTPRE; MGAT4A; MAGT1; CDYL; OCRL; KIAA0774; IFIT5; FAM49A; PECAM1; KBTBD11; IFIT3; IFI44L; C17orf60; MRC1; PDE4B; NGFR; KCTD12; TNNI2; GNA11; CAMK1; RRAGB; CD1C; CDC42BPB; PLVAP; LGMN; HSPA1A; IRF8; TLR1; C10orf10; IFIT1; CIDEB; SIGLEC15; ASB9; OGFRL1; RPS6KA3; RBM47; PLCH1; GSPT2; TLR2; DDEF1; CSF3R; ALOX5; CELSR1; DLG3; SH3BP2; PRKAR2B; CYBB; CYTL1; ECHDC3; NDE1; CBR1; ARHGEF10; PIP3-E; LYST; IL6R; EFNB1; IFNGR2; PDXK; MX1; SETBP1; LOC57228; ALDH3B1; LTB4R; CEBPE; SH3BP5; HDHD1A; ITSN1; MYBPC2; ACOT9; SCML2; MS4A6A; PLP2; ADAM8; SCML1; C10orf56; IL13RA1; MORC4; IL3RA; ZNF185 |
| Normal | SMARCE1 | <0.001 | 0.0326 | - | down | 1000 | 42 | NDUFA1; COPS3; COX6C; COX7B; FRG1; RBBP7; PSMC5; PSMB3; ATP5J; HMGN1; ENSA; SLC25A5; NDUFB8; ATP5G1; EIF4A3; NDUFS6; COX6A1; RWDD1; PSMD2; SF3B5; PGAM1; NDUFS1; PSMA3; MCTP2; TP53TG3; CCR5; APOL6; GRIK2; RASL10A; EGFL7; KAL1; FLRT3; EFR3B; PRKG2; ZNF239; ZNF192; CSHL1; REEP1; FLT1; FER1L3; AGMAT; TXNRD3 |
| Pseudodip | SMARCE1 | 0.003 | 0.08862 | - | down | 2500 | 39 | SLC39A8; C4orf41; SIRT1; RRAGA; NUP54; RAB5A; CD46; PSMC6; COPS4; ICK; ATXN3; ARHGDIB; HNRPH2; NDUFB8; PIN4; MRCL3; BZRAP1; GRIK5; SSH3; HRK; ANXA4; E2F2; ERBB2; GPR176; SH2B2; PCDH9; CXCR3; DLGAP4; dJ222E13.2; HCRP1; MPO; SERHL2; PYCR1; COL9A3; TAP2; ATM; SLIT2; PALM; SFRS6 |
| TEL-AML1 | SMARCE1 | 0.009 | 0 | - | down | 1500 | 271 | NME2; C1QBP; CCDC28A; DECR1; PTPN2; AMD1; RP3-377H14.5; BRP44L; MBD2; MRCL3; ACAT2; CSGALNACT2; ME2; C8orf33; ATP5G1; SRP72; OLA1; C14orf108; MRLC2; PSMG2; SLC25A5; UTP18; EIF2S1; RPL23A; RSU1; MRPS35; RPL19; SF3B5; SMAP1; PSMD9; SF3A3; SRPK1; NDUFV2; BTF3; STRAP; STX6; APEX1; MRPS18B; IDE; DDX47; TCP1; SNRPD1; MRPS10; MRPL9; FAM110A; GTF2H5; NUP98; RPIA; HSPA14; LDHB; NOL7; IGBP1; CCDC53; CPSF6; MRPS30; VPS4A; CUTA; ZNF22; EIF2B2; C14orf166; ATP5H; ARPC5; DHX40; SEH1L; CCT2; GTF2A2; CAMK2G; SNRPE; PFN1; ZNF32; C14orf156; THOC7; KIAA0776; NDRG3; HSP90AA1; PARK7; PSMA4; UXT; WRB; GIMAP5; UAP1L1; SUOX; SPP1; HEY2; DHRS3; TP63; CD74; EPB42; SP4; TMCC2; ATF5; MPL; ALAS2; SLC35F2; ERAF; DNM1; GYPB; PCDH9; SCARB2; SOCS2; ODZ4; AGPAT2; SELENBP1; MCTP2; OAS3; NRXN3; HLA-DOA; IGKV1OR2-108; DOK3; SLC4A1; HLA-DRA; TP53TG3; IGHA1; LARGE; SLC12A6; MORC1; HLA-DPB1; MAMLD1; AKAP12; MDM2; HLA-DRB1; APBB1IP; DUSP26; GYPA; CA2; GIMAP4; PFTK1; TSPAN5; TMEM140; HLA-DRB5; CTGF; UBE2H; WFS1; TCL6; TCL1B; COBL; TXNIP; MYLK; GTDC1; SSX1; CLCN7; HIST1H2BG; HRK; SSX4; CCR1; MEF2C; CD40; LYL1; CEACAM1; HPS4; EFEMP2; C1orf78; ATN1; PTPRG; VPREB3; UTRN; SSX2; FADS3; AMIGO2; MAGED4B; PYHIN1; NPR1; C12orf49; MYO5C; DIP2C; IL18RAP; CD79A; ZNF117; LRP8; LPHN3; FGD6; CACNA1A; FGD1; HS3ST1; OCA2; GAB1; FLRT3; COL5A1; CHL1; SLC27A3; PFKFB2; DPEP1; FHIT; POU2AF1; INSR; SIDT1; TRIB1; SCHIP1; IL1RAP; ARHGAP24; C15orf5; ITPR3; EGFL7; NOL4; PRO2268; CCNJL; EPHA7; CKMT2; TBC1D8B; STK32B; RICS; GPR17; LGALS7; DSC2; DUOX1; CACNB2; SPON1; CD27; MSR1; AJAP1; SDC1; KHDRBS3; NARFL; SMAD1; FARP1; FERMT2; GRK5; SEMA6A; EHD2; SDC2; TFPI; SCN3A; LONRF1; SH3GLB2; SCARB1; HLA-DOB; ANGPTL2; H1F0; STAG3; DLGAP2; GNG11; SPTA1; NRTN; PID1; MAPK13; ABCG2; MDK; EPOR; PLAG1; GREB1; C13orf18; PTP4A3; RAG1; NRN1; TRPM4; SEMA3F; CXCR7; TSPYL5; PDLIM7; TERF2; DRAM; PTPRK; MYO10; NR3C2; SPANXA1; KCNK3; TUSC3; GBA3; ARHGAP29; TNFRSF21; TMEM16A; LOC654342; NOVA1; HAP1; FBN2; TCFL5; TNS1; PCLO; BIRC7; CLIC5; ARHGEF4 |
| E2A-PBX1 | SMARCA5 | <0.001 | 0 | - | down | 2500 | 570 | CIRBP; MSN; LASP1; KIAA0430; TM9SF3; SNAP23; ITGB1; UQCR; ITM2B; AMZ2; OGT; TMED10; CNDP2; RNF13; UBE4A; EXOC1; CSNK1D; PPP2CB; GMFB; TMEM30A; UBE3B; KIAA1109; ZFP106; FAM134C; SFRS18; TMEM189-UBE2V1; TUBGCP2; ZZZ3; RPN2; C2orf28; ATP6AP2; UBE2D3; PAFAH1B1; UNC50; RP6-213H19.1; ARL8B; WIPF1; SSR4; WSB1; SERINC1; ZNF706; TM9SF1; GMPR2; ARF3; TMEM131; ZMYM4; ELMO2; RAB1A; ITFG1; TMEM59; HTRA2; C3orf63; HELZ; MAP2K1; ZDHHC7; MAPK14; MCM3AP; IL10RB; RHOT1; HERC1; VAMP3; CMTM6; GLOD4; CUL5; LAMP1; ISCU; RPN1; RNF111; VTI1B; PGK1; SCAMP1; PIGF; SFRS17A; ARHGAP1; SLC38A2; FOXJ3; TMEM49; ZDHHC17; FNTA; SPTLC1; BTAF1; ST13; TXNDC14; C15orf24; UBR4; GPBP1L1; TMEM111; GTF3C3; FTHP1; NT5C2; SMCHD1; DOCK2; TMEM39B; MRFAP1L1; NMT1; FRYL; UXT; GAK; PIGG; RBL2; KIDINS220; TRIM33; LRRC47; ACTR10; GGNBP2; ATP6AP1; TBC1D25; OSBPL2; MAPRE2; ROCK1; NISCH; NUDT9; CDIPT; UBE2Z; ZFAND6; C19orf56; SFRS5; RNASEN; C5orf15; PAPOLA; MDN1; C21orf66; VPS26A; TMEM1; KIAA0494; RAB6A; ATRX; DENR; PSMB5; EIF4A2; ASF1A; PIP4K2B; NDUFS4; NDUFB11; DMTF1; PPT1; TMEM123; FBXW4; SENP6; DCN; PI4KAP1; FBXL5; VAMP7; FOXK2; MTCH1; RNMT; TM9SF2; DCK; RAB7A; ARL1; PANK4; CUL4B; RAB6C; PJA2; METAP1; PNN; DCTN2; DERL2; SDF2; C19orf60; ANAPC13; DDX3X; HNRPA3P1; GPATCH8; ATP6V0C; CUL2; MSL-1; HISPPD1; PDHA1; GORASP2; RNF10; SEC63; TTC3; RCN2; ADAR; ZDHHC6; METT11D1; NMI; ZNF574; LASS2; PHF8; IRAK1BP1; NIF3L1; TLK1; PCNT; MPHOSPH9; HNRPH3; METTL3; SPEN; MAN1A2; SUB1; RAB2A; ZMPSTE24; MARK3; CYB5R3; ZNF294; MYST4; UBP1; RALBP1; RAB14; SON; ANAPC5; UTP3; BCAP31; DYRK1A; SAP30BP; TMEM147; ALG5; CBWD1; BAT1; PGS1; CNPY2; KIAA0265; SEC31A; BECN1; RBM26; FTSJ1; ZFR; ATP6V1H; KLHDC2; CANX; EBAG9; PCID2; PRKRIR; CAB39; PLEKHB2; ACSL3; MMS19; SUMO2; DNAJC13; HUWE1; PHF3; HNRPDL; VAPB; PMS1; C10orf76; NOLA3; SRF; SDCCAG1; C10orf6; ANAPC10; SMAD4; ALG8; SH3BGRL; RBM25; TXNDC15; SLC35A1; STARD7; LAGE3; VPS28; TMEM66; TARS; CSNK1A1; GMFG; NEK9; NOMO2; SEC24B; PGRMC1; SECISBP2; EIF2AK1; LIN37; ARMCX6; LOC130074; RRN3; TMEM208; MED13; DDX42; ZBTB1; ERCC5; USP9X; RABGGTB; ACIN1; RNF139; MED17; TCF25; DGCR2; NPEPPS; RBM39; RDH14; BRWD2; SS18L1; FAM21A; PSMD10; ZCCHC8; RPL36AL; RSRC2; RRP1B; PPIB; SLC30A5; TXNL4A; LAPTM4A; KIAA0196; DUS1L; TIAL1; C6orf211; SMC3; ATP6V1E1; QRICH1; SMARCA5; PHKB; DKC1; MARCH7; RNMTL1; ZNF518A; ALG6; INTS12; SSR1; TINAGL1; SLC22A5; ADCK2; DEPDC1; PLAC4; NEBL; HIST1H2BN; RALGPS2; CD19; BSPRY; ARL4A; ZNF528; DDR1; ZBTB6; ABCC4; TMEM63A; TAT; TPST1; KMO; OR7E38P; MYOZ2; TCL6; COL1A1; PARD6A; C6orf60; SLC35F5; NCF1; MPP6; ANKRD55; TRAK1; TULP4; VIPR2; CD9; ZNF669; ZNF257; RGS5; BCL9; VPREB3; BAMBI; BTK; EPAS1; ERO1LB; C14orf132; CDC14B; EHBP1L1; E2F2; SEMA6D; RASIP1; KCTD20; MUC5AC; CD24; ZNF771; MARCH3; FADS3; TCF4; PLGLB2; TERT; MLLT4; NR1D2; EGLN1; PHLDA2; GINS4; ENTPD3; HIST1H4C; CCDC69; BCAS4; LRP4; PRL; CLEC2D; VASH2; SPPL2B; AKAP12; SORT1; SPINT1; PCSK6; ITIH4; RNF144A; CPM; CRMP1; GJC1; FLJ13769; SLFN12; MLPH; E2F1; C6orf124; MYLK; AASS; DYRK3; TJP2; KCNJ2; C12orf32; BASP1; LOC91316; RAI14; AEBP1; E2F5; KCNJ3; RHOQ; SPA17; SNTB2; CEACAM21; IL8; CD84; FERMT1; FAM64A; CD48; PIP5K1B; VIPR1; SEMA5A; MDM4; PTTG3; ZSCAN16; PCSK5; LRIG1; PADI4; NINJ1; CD38; ITIH3; KIF13A; CD72; PAX5; NOTCH2; LRRN2; PLS3; FLJ20674; PDE4D; ALDH3A2; RHOB; ATRNL1; IGLL1; RHBDL2; GLDC; TRAK2; GPATCH2; RBBP5; ADAM19; TRIM16; AUTS2; C1orf135; NFATC4; BMPR2; IQSEC1; CELSR2; RAG2; ARHGAP8; TTC9; ST6GALNAC4; COBL; ZNF124; ARHGEF11; ZNF385D; TCF3; LOC90925; IL7R; SPAG6; SORBS2; MYT1L; MYL4; JAM2; PAWR; IGHM; COCH; GATM; WASF1; PLXNB1; SH3PXD2A; PPFIBP1; KCNJ16; TNK2; MAP1A; BIK; FAM152A; VDR; TMSL8; CYFIP1; EMP2; P4HA2; BACH2; NRGN; KIAA0040; IL1B; PRR5; CORO2B; PPFIA4; ARNTL2; MAP3K1; PHACTR1; SH3BP4; CD58; EWSR1; FLI1; F13A1; TST; LTBP2; DOCK9; RHOBTB1; CASC1; TRIB2; TGFBR2; MAP1B; FGF9; GPR176; PSAT1; PIGZ; SLC15A2; CSF2RB; ODZ4; ENDOD1; D4S234E; QRSL1; TSSC1; GNAZ; KCNA3; ARHGEF9; FNDC3B; ALDH1A1; GALNT14; SYT1; TMEM121; KIAA0922; DOCK10; CALD1; RASAL1; ELOVL2; CCDC81; IL12RB2; IGSF3; APBB2; EAF2; KCNMB3; PLEKHF2; ELL3; BLK; IRF4; KIAA1305; AOX1; ROR1; SAMD4A; LRMP; PITPNC1; KANK1; ADARB1; DACT1; KCNJ12; FAM3C; GOLGA3; FAT; NCAPD3; NP; FHOD3; SORBS1; SEMA4C; SLAMF1; GP5; HIP1R; NID2; PRKCZ; KIAA0802; SYNPO; PSEN2; SLC27A2; MERTK; PBX1 |
| Normal | SMARCA5 | <0.001 | 0 | - | down | 1500 | 75 | LOC552891; HPRT1; PRDX3; NDUFA1; COX7B; FRG1; LAGE3; RBBP7; PSMC5; ARMET; UBE2A; ATPIF1; ATP5J; HMGN1; COPS6; UCRC; FLII; BCAP31; C21orf59; PECI; NDUFB8; CCDC56; ARL6IP1; COX6A1; RWDD1; PSMD2; PGAM1; MRPL13; PSMD4; MDH1; NDUFS1; PSMA3; HSPA5; MCTP2; GPHN; RIMS3; TP53TG3; CCR5; APOL6; GRIK2; RASL10A; PDE8B; TOX3; KAL1; HIP1; AKAP12; FLRT3; EFR3B; PRKG2; ABCB4; ZNF239; PTGDR; CD84; ZNF192; CSHL1; CDH11; ANGPT2; REEP1; FLT1; RAG2; LRRC8B; PCDH9; PCBP4; GLDC; RHBDL2; FER1L3; SLC6A16; AGMAT; TXNRD3; MPPED2; HEY2; LHFPL2; STAP1; PTPRM; CENTG2 |
| MLL | SMARCA4 | <0.001 | 0 | - | down | 400 | 44 | MGC29506; ZNF43; MYH10; AKAP12; ITPR3; HYI; HPS4; FAM65A; DBN1; MTF2; PBXIP1; PTP4A3; ALDH5A1; SCMH1; CDCA4; POGZ; RAB11A; NRN1; WSB2; RHBDF2; RHOQ; EHD4; RGS16; FOSL2; DUSP3; SPG20; FLT3; STARD13; VNN1; PSCDBP; P2RX5; PTGES3; RNASE6; SIDT2; C11orf24; CD44; GPM6B; SCPEP1; NLRP3; MAP7; LGALS1; KLRK1; CCNA1; C20orf103 |
| Relapse | SMARCA4 | <0.001 | 0 | - | down | 1000 | 23 | DBN1; TUBB; SEPHS1; HRB; ANP32E; KIF20A; MYBL2; TIMELESS; ZNF675; MYH10; CDK2AP1; DCP1A; C11orf21; LGALS1; FAM13A1; S100A4; P2RX5; IDH1; NPR3; CD302; LTBR; CCPG1; TSPAN32 |
| TEL-AML1 | SMARCAL1 | <0.001 | 0.04189 | - | down | 2000 | 484 | NME2; NOL14; CCDC86; FAM120A; C1QBP; LPXN; NIT2; DECR1; EEF1B2; GMDS; ACOT7; AMD1; BRP44L; FAM98A; TIMM13; DPP3; MRPL3; CTSC; APOO; MBD2; TTLL12; CASP4; GTPBP6; PPIF; PAICS; LETM1; ME2; PSD4; SRPRB; SRP72; WARS; ABCE1; OLA1; ICT1; C14orf108; MRLC2; PSMG2; LTA4H; C6orf66; NAT10; NPM1; NSF; C15orf15; UTP18; ZNF259; SRI; EIF2S1; EIF3M; HEATR3; RSU1; WDR43; PFKP; MRPS35; HADH; DHRS7; RPL26; SF3B5; PSMD9; HSP90AB1; IL2RG; TMEM109; KCNAB2; RABEPK; SF3A3; SRPK1; GART; NDUFV2; RAP2B; NOC3L; MRPS12; MTHFD2; LSM4; BTF3; ATIC; STRAP; APEX1; MRPS18B; C12orf10; IDE; PROSC; TADA3L; LRPPRC; DDX47; HINT1; MRPL23; TCP1; STARD7; SNRPD1; SLC7A1; MRPL9; APEH; R3HCC1; RPL10A; NUP98; MRPL4; HSPD1; RPIA; IMPDH2; POLR3E; MRPS2; ATP2C1; LGTN; LDHB; CLNS1A; NOL7; RPS27L; BZW2; CCDC53; FASTKD2; POLR2L; MRPS30; VPS4A; AGL; CUTA; UBA5; COX17; ADA; EIF2B2; C14orf166; GSTP1; RPL12; ATP5H; EBNA1BP2; PCM1; NDUFC1; PHB2; PIK3CB; TRAPPC6A; SHQ1; CCT2; GNL3; WDR3; GTF2A2; RPL13; SNRPE; APRT; FAH; PFN1; ITGAE; VAMP3; RPL35; USP20; ZNF32; C14orf156; MAPBPIP; TP53AP1; THOC7; IKBKAP; SLC3A2; EPRS; C12orf11; TXN; ETFB; SNRPF; HSP90AA1; LARP1; TDRD3; PARK7; LANCL1; PSMA4; UXT; WRB; WDR67; GCSH; AP2S1; TEX261; ANKRD27; RPL18; ALDOA; TTC19; ATP13A3; ATMIN; TIMM17A; FBL; SMYD3; COX8A; MDH2; RPS5; MTX1; DRAP1; POLR2H; NOLC1; PRKDC; OSTM1; SLC25A15; LOC730107; ENOPH1; TSFM; KIAA0152; DYNC1H1; TM2D3; PWP1; TOMM70A; EIF3I; C7orf24; DLAT; XPOT; CNIH; CLPP; TUFM; SMARCE1; WDR18; LEPROTL1; C11orf73; RRP1B; NOL11; BXDC5; NOLA2; CBFB; ATP6V1F; ASH2L; MIF; NOL1; TXNL1; C3orf28; HNRPAB; EIF3J; DLG3; SFMBT1; SUOX; SPP1; HEY2; SCARF1; TP63; CD74; KIAA0427; SP4; TMCC2; CSDA; SNX2; POU4F1; HIST1H2AG; MPL; CSF2RB; ALAS2; SLC35F2; PRSS7; ERAF; INPP5D; RP4-691N24.1; ZNF652; LAPTM5; FAIM3; GYPB; PCDH9; SCARB2; CRK; SOCS2; TCF4; C21orf7; ODZ4; HIST1H2AE; AGPAT2; MCTP2; ITIH3; PHTF1; OAS3; NRXN3; HLA-DOA; IGKV1OR2-108; DOK3; SLC4A1; LIN7B; HLA-DQB1; HLA-DRA; MAP4K2; ST8SIA4; IQCK; EPHB4; SH3TC1; TP53TG3; SMTN; KCTD7; IGHA1; CEBPE; LARGE; HLA-DPB1; MAMLD1; HLA-DPA1; AKAP12; MDM2; SCML2; HLA-DRB1; APBB1IP; DUSP26; FAM127B; GYPA; HIST1H2AJ; NPY; SERPINB9; HIST2H2AA3; IRAK3; INPPL1; CD52; GIMAP4; PFTK1; HIST1H2BI; TMEM140; HLA-DRB5; TNS3; CTGF; ALDOC; CACNA2D2; HIST2H2BE; E2F5; PTPRE; HIST1H2BE; WFS1; NCKAP1; DGCR6; TCL6; TCL1B; COBL; DYRK3; TXNIP; CHD7; CTNNA1; MYLK; GTDC1; SSX1; CLCN7; HIST1H2BG; HRK; HLA-DRB6; HIST1H2BH; SSX4; LRRN2; TRPV1; MEF2C; CD40; KANK2; CEACAM1; HPS4; EFEMP2; HLA-DMA; HLA-DMB; ZCCHC14; EFNA1; CRIM1; ATN1; PTPRG; CSF3R; tcag7.1314; MME; VPREB3; HHEX; CLCC1; UTRN; PDK1; SSX2; FADS3; ZNF274; MGC5370; RGL1; PYHIN1; NPR1; C12orf49; MYO5C; DIP2C; IL18RAP; FAM127A; IRS2; ERG; HIST1H2BF; NEIL1; CD79A; C1orf38; ZNF117; SIGLEC15; IL6ST; JUP; LPHN3; FGD6; CACNA1A; FGD1; TRIO; LILRA2; OCA2; MLXIP; LDOC1; GAB1; FLRT3; COL5A1; CHL1; SLC27A3; RALGPS1; PFKFB2; DPEP1; FHIT; POU2AF1; FOXO1; GTF2IRD1; INSR; SLC35E3; PTK2; FAM65A; BTN2A2; SCHIP1; ARHGAP24; EGFL7; PIK3IP1; C14orf132; ALOX5; NOL4; PRO2268; AK1; CCNJL; EPHA7; ISG20; BMP2; GNG7; ELK3; CKMT2; PSD3; LASS4; SOX11; TBC1D8B; EFNB2; STK32B; GPR17; DSC2; DUOX1; GABBR1; CRMP1; CACNB2; SPON1; EIF2AK3; ZNF91; MSR1; SDC1; KHDRBS3; NARFL; SMAD1; FERMT2; SEMA6A; SDC2; ARHGEF12; SCARB1; HLA-DOB; TBC1D9; ANGPTL2; H1F0; STAG3; DLGAP2; GNG11; SPTA1; CYB5R2; WDFY3; PID1; MAPK13; ABCG2; MDK; PLAG1; GREB1; C13orf18; PTP4A3; RAG1; FUCA1; NRN1; TRPM4; SEMA3F; TSPYL5; PDLIM7; TERF2; DRAM; PTPRK; MYO10; NR3C2; SPANXA1; KCNK3; GBA3; ARHGAP29; TNFRSF21; TMEM16A; LOC654342; NOVA1; HAP1; FBN2; TCFL5; TNS1; PCLO; BIRC7; CLIC5; ARHGEF4 |
| Hyperdip>50 | PRDM2 | 0.004 | 0 | - | down | 1500 | 293 | GPATCH2; EWSR1; PIGB; ITPA; EFHA1; LOC51035; ELF1; PAIP1; SMARCA4; PDE8A; GADD45A; TPD52; SPTBN1; AKAP12; YLPM1; PSME4; NADK; TTC15; RPS6KA2; HPS4; ADAM17; STK39; SENP7; RNF41; SYK; NGLY1; OPN3; USP4; MOBKL3; GFPT1; LBA1; PTBP2; PAK2; VPS37B; MZF1; DKFZP564O0523; SMG1; SR140; LRP5L; UCKL1; ANKZF1; CAPN7; SSBP2; SFRS12; TSC2; TGFBR2; ADNP; DIP2C; C9orf45; STUB1; UBE2N; SNRP70; TERF1; FASTKD5; CLEC16A; C5orf5; ZNF586; ARID1A; CHP; SELT; BTG1; ZHX2; ZC3H7A; GNB1; Gcom1; SGSM3; MYO9B; IRF3; ATR; CCNL2; POU2F1; TMEM87A; ANKHD1; ARHGEF7; FUBP1; TWF1; TCL6; SFRS8; PIK3R1; TSSC1; ZMYND8; APC; FADS3; MARCH7; PPP6C; LOC440354; P2RX1; PRDM2; YTHDC2; NUDT4; F11R; TTC17; CUX1; RY1; UBA7; ING3; ROCK2; SLC7A5P1; DDEF2; ARPP-19; GABARAPL2; STK24; SLC25A36; MARS; SPSB3; SMPD4; PELI1; BZW1L1; RNASEH1; HNRPK; TTC13; TAPT1; CDC2L5; ZNF238; NFIL3; TMCC1; SLC35D1; LEMD3; HIATL1; C1orf80; ATP1B3; TRAK1; ATG12; SNX26; C1orf108; WEE1; SERTAD2; CGGBP1; ZNF12; LMF1; HNRNPA1; SF3B1; ARHGAP17; C3orf37; TCL1B; GTDC1; RBM5; ETHE1; SCNN1A; LY6E; COL18A1; AFG3L2; LAGE3; QDPR; TACC3; FAM45B; PTRH2; ALOX5AP; IL2RG; MAD2L1; EIF2AK2; FXYD6; PTTG1IP; EPHB3; KIAA1279; ITGB2; NUP214; GTF2H5; BEX4; TEX2; MED9; GPR132; USP18; ARMCX2; HRSP12; PGRMC2; IFI44; TIMP2; RFC1; HDAC2; APOO; IFI6; ENTPD6; IER3; ST7; DAPK1; ANKRD28; PWP2; TREML2; HSD17B8; E2F3; C16orf62; PDGFA; C4orf27; CFP; KIAA0513; PGRMC1; KIF4A; PLEKHA1; C11orf67; C1GALT1C1; CANT1; IMPA2; UBL4A; DYRK4; HERC6; C10orf119; GCN5L2; C17orf71; PRPS2; NEO1; GSTZ1; OAS1; ZNF516; FUT7; PSMG1; MICA; PLEK; KIAA1462; F2RL1; LSP1; ARMCX6; IL10RB; C21orf45; DLG5; EBP; NFE2; FAM26B; TK1; IL17RA; CSTB; NXT2; DDX60; MCTS1; TMEM165; ERCC6L; KIAA1166; TAF9B; VPS26A; ALDH6A1; SPIN2B; SUMO3; IFIT3; IFI44L; NDUFB11; H2AFY2; STCH; NGFR; PJA1; NSDHL; SUV39H1; TNNI2; CAMK1; CAPN2; UCHL5IP; CD1C; ORC3L; PLVAP; TIMM17B; TMEM164; PIN4; ACOT2; SLC19A1; CHAF1B; IFIT1; TMEM187; ASB9; POLA1; RBM47; C21orf33; PLCH1; PET112L; RRP1; RRP1B; DLG3; MGC39900; GPKOW; SOD1; CYBB; ECHDC3; PIP3-E; LYST; IL6R; EFNB1; PDXK; ARMCX1; MTCP1; PIGP; DHRS4; CEBPE; SH3BP5; PSMD10; HCCS; ITSN1; MYBPC2; MS4A6A; TCEAL4; ADAM8; C10orf56; IL13RA1; MORC4; IL3RA; ZNF185 |
| Normal | SMYD3 | <0.001 | 0 | - | down | 1500 | 72 | PSMA7; MRPL34; COPS3; COX6C; NANS; AKR7A2; SCCPDH; UCRC; TIMM8B; ENSA; FH; HMGN3; MAPBPIP; TUBA1C; NDUFB8; ATP5G1; NDUFA6; NDUFS6; GPI; RAN; COX6A1; SFRS2B; INTS7; MRP63; PSMD2; SF3B5; TUBB2C; PGAM1; MRPL13; MDH1; NDUFS1; HSPA5; NDUFA8; PPP1R16B; TP53TG3; CCR5; MYLIP; APOL6; GRIK2; MXI1; RASL10A; PDE8B; EGFL7; AKAP12; GNG11; FLRT3; CHD7; EFR3B; PRKG2; ZNF239; PTGDR; CSHL1; RXRA; CDH11; LIG4; REEP1; FLT1; CASK; PCDH9; PCBP4; AP1B1; FER1L3; SLC6A16; AGMAT; PBX2; MPPED2; HEY2; CHST7; GFOD1; STAP1; PTPRM; CENTG2 |
| TEL-AML1 | SMYD3 | <0.001 | 0 | - | down | 750 | 195 | SORD; CCDC86; FAM120A; C1QBP; NIT2; EIF4EBP1; DECR1; PTPN2; CABC1; ZNF593; GMDS; NME1; AMD1; BRP44L; FAM98A; TIMM13; DPP3; MRPL3; CTSC; ALDH9A1; TTLL12; ACAT2; PPIF; PAICS; FDX1; ATP5G1; SRPRB; SRP72; ABCE1; OLA1; UCHL5; WAPAL; C6orf66; NAT10; KLHL21; UTP18; ZNF259; SRI; EXOSC5; EIF3M; TFB2M; WDR43; PFKP; MRPS35; SF3B5; SF3A3; GART; NOC3L; MTHFD2; LSM4; ATIC; HSPC111; BRP44; NOC2L; STRAP; XTP3TPA; SNUPN; SLC1A4; UCHL3; LRPPRC; PLXNA3; HIST2H2BE; PTPRE; HIST1H2BE; WFS1; DGCR6; TCL6; TCL1B; TXNIP; CHD7; SSX1; HIST1H2BG; HRK; HLA-DRB6; HIST1H2BH; SSX4; CCR1; CD40; KANK2; CEACAM1; EFEMP2; HLA-DMA; HLA-DMB; AP1B1; EFNA1; CRIM1; PTPRG; tcag7.1314; MME; VPREB3; UTRN; SSX2; ST3GAL6; FADS3; MGC5370; RGL1; TSPAN14; NPR1; C12orf49; MYO5C; DIP2C; FAM127A; ERG; HIST1H2BF; NEIL1; CD79A; C1orf38; ZNF117; SIGLEC15; JUP; CLMN; LPHN3; MEST; CACNA1A; FGD1; LILRA2; MLXIP; LDOC1; GAB1; FLRT3; COL5A1; PXN; SLC27A3; DPEP1; FHIT; POU2AF1; FOXO1; INSR; SLC35E3; PTK2; FAM65A; BTN2A2; LTB; SCHIP1; ARHGAP24; EGFL7; PIK3IP1; ALOX5; NOL4; PRO2268; EPHA7; ISG20; BMP2; GNG7; ELK3; CKMT2; PSD3; LASS4; TBC1D8B; STK32B; GPR17; DSC2; DUOX1; GABBR1; ZNF91; SDC1; NARFL; SMAD1; FERMT2; SEMA6A; SCN3A; HLA-DOB; TBC1D9; H1F0; STAG3; DLGAP2; GNG11; SPTA1; WDFY3; PID1; MAPK13; MDK; C13orf18; PTP4A3; RAG1; FUCA1; TRPM4; SEMA3F; TSPYL5; TERF2; DRAM; PTPRK; NR3C2; SPANXA1; KCNK3; GBA3; ARHGAP29; TMEM16A; NOVA1; HAP1; FBN2; PCLO; BIRC7; CLIC5; ARHGEF4 |
| Normal | EHMT2 | <0.001 | 0 | - | down | 1500 | 76 | NUCKS1; MRPL34; TRMT5; KIAA0101; TYMS; HMGN2; GSTP1; UCHL5IP; PSMC5; MAGOH; PSMB3; TBC1D25; ATP5J; PGD; HMGN1; COPS6; FLII; BCAP31; C21orf59; RCOR1; TUBA1C; MCM6; RFC4; PDIA6; CCDC56; EIF4A3; H2AFZ; CBX5; GPI; ARL6IP1; CKS1B; RAN; SFRS2B; SHMT1; PSMD2; TUBB2C; U2AF1; PSMD4; NDUFS1; PSMA3; AMT; MCTP2; TP53TG3; CCR5; APOL6; GRIK2; CBLB; PDE8B; TOX3; KAL1; FLRT3; EFR3B; PRKG2; ZNF239; CD84; ZNF192; CSHL1; RXRA; CDH11; ANGPT2; ITGA6; REEP1; FLT1; PRUNE2; RGS9; PCBP4; FER1L3; SLC6A16; AGMAT; TXNRD3; MPPED2; HEY2; LHFPL2; CHST7; STAP1; CENTG2 |
| Normal | CBX3 | <0.001 | 0 | - | down | 1500 | 56 | TRMT5; COX6C; RBX1; COX7B; HMGN2; PSMB3; ATPIF1; COPS6; UCRC; ENSA; TUBA1C; MCM6; NDUFB8; RFC4; H2AFZ; NDUFA6; NDUFS6; ARL6IP1; RAN; COX6A1; SFRS2B; MRP63; PSMD2; SF3B5; PGAM1; MRPL13; MDH1; NDUFS1; TP53TG3; CCR5; MYLIP; APOL6; GRIK2; RASL10A; PDE8B; KAL1; FLRT3; CHD7; EFR3B; PRKG2; ABCB4; ZNF239; ZNF192; CSHL1; CDH11; ANGPT2; REEP1; FLT1; PCBP4; FER1L3; SLC6A16; AGMAT; PBX2; MPPED2; LHFPL2; GFOD1 |
| Normal | CBX1 | <0.001 | 0.02215 | - | down | 500 | 60 | NUCKS1; PSMA7; TRMT5; NDUFB2; COX6C; RBX1; TOR3A; MAGEF1; RNPEP; HMGN2; SS18L2; PSMB3; BID; UCRC; SYNJ2; RCOR1; MAPBPIP; CCNB2; TUBA1C; RPA3; HN1; CBX1; H2AFZ; NDUFA6; NDUFS6; CKS1B; COX6A1; SFRS2B; MRP63; PSMD2; SF3B5; FKSG30; MRPL13; NDUFS1; CD34; TP53TG3; MYLIP; MXI1; RASL10A; PDE8B; EGFL7; CHD7; EFR3B; PTGDR; ZNF192; CSHL1; CDH11; CREG1; ANGPT2; REEP1; FLT1; CAPN3; AGMAT; LGR5; PBX2; HEY2; LHFPL2; GFOD1; STAP1; CENTG2 |
| Pseudodip | CBX1 | 0.002 | 0 | - | down | 1500 | 20 | NDUFS5; GLUL; PNMA1; MAGEF1; PSMC6; COPS4; NDUFB8; SNRPN; GRIK5; SSH3; HRK; ANXA4; SMC6; ERBB2; MPO; SERHL2; TAP2; TLE4; SLIT2; PALM |
| Relapse | CBX1 | <0.001 | 0 | - | down | 1500 | 33 | TUBB; TOP2A; ARHGAP19; TPX2; SPAG5; MAGED1; ANP32E; KIF20A; CDC25B; CCNB2; BUB1; BIRC5; MAD2L1; AURKB; AKR1B1; KIF2C; H2AFZ; CDK2AP1; CKS1B; RAD51; C11orf21; CISH; CDC42EP3; NPR3; AMBRA1; LEPROT; PSTPIP2; FOXP1; LTBR; BAALC; FLJ13197; CCPG1; TSPAN32 |
| BCR-ABL | CBX4 | <0.001 | 0 | - | down | 750 | 108 | DGKD; SETD1B; BCOR; CLASP2; ZEB2; ODC1; ULK1; ARL4A; WDR19; BCR; BCL7A; VLDLR; SLC5A3; MFAP3; LGMN; ST3GAL5; DAB2; LST1; YES1; SNX7; C11orf75; IFITM1; LARGE; CD52; MED9; PECAM1; CCR6; A2M; IFITM2; ABLIM1; EFNA1; ID3; BACE1; GPR56; FAM13A1; NLGN4X; ZNF467; DNTT; HLA-DQA1; XBP1; CEACAM6; TMEM156; DUSP26; CD34; SERPINB9; CAMSAP1L1; CEP68; ENPP2; OR7A5; MRC1; LXN; IGFBP4; GPR171; MYO5C; RBM47; NAV1; SLC35D2; TNFRSF14; SV2A; SCHIP1; SP140; FZD6; MYO1B; ARHGAP22; PDE9A; PHF15; ITGA6; CENTD1; IL2RA; LAMA3; C6orf32; BMPR1B; SERPINB6; GIMAP4; NT5E; MTSS1; ITGA5; SECTM1; CTNND1; FLJ14213; CCND2; APOL3; GLS; S100A13; OSBPL10; P2RY14; GIMAP6; RAPGEF3; EMP1; ARHGEF17; ASB13; FAM129A; PRX; IGJ; COL6A3; NPDC1; CASP10; CNN3; PSTPIP2; ECM1; SPARC; SLC2A5; TMEM204; LOC26010; OLFML2A; CTDSPL; NRP1; PON2 |
| Hyperdip>50 | CBX4 | <0.001 | 0 | - | down | 500 | 59 | RNF40; EFHA1; ODC1; MCRS1; ELF1; H1FX; C1orf160; C9orf16; RER1; PSME4; NADK; RMND5A; PRKCSH; SF3A1; IQCC; USP4; VPS37B; MZF1; DDA1; BTBD2; RHOT2; NME3; SFRS12; TSC2; RHOBTB3; RGL1; FGD1; DLG5; CLEC4E; ERG; KIAA1166; EXDL2; IL1B; PPAP2B; MAGT1; OCRL; PECAM1; MRC1; STCH; NGFR; SAT1; CAPN2; LGMN; C10orf10; ASB9; MPP1; RBM47; PLCH1; GSPT2; ALOX5; CYTL1; PIP3-E; SETBP1; PIGP; LOC57228; SH3BP5; ITSN1; TCEAL4; IL3RA |
| Pseudodip | MYST2 | <0.001 | 0 | - | down | 750 | 17 | BACH1; C4orf41; SIRT1; NUP54; RAB5A; TMEM164; COPS4; LAMP2; HNRPH2; GRIK5; ATP1A3; GPR176; PCDH9; LMF1; COL9A3; AEBP1; SLIT2 |
| E2A-PBX1 | MYST3 | <0.001 | 0 | - | down | 1000 | 234 | CIRBP; CTDSP2; KIAA0430; SERINC5; PRKCB1; AMZ2; STX16; SLC35E2; C12orf35; DGAT1; STAT5B; CSNK1D; PPP2CB; C14orf159; GMFB; KIAA1109; RPS19; CBFA2T3; SFRS18; TMEM189-UBE2V1; ZZZ3; CLINT1; UBE2D3; C10orf18; ABCC1; PAFAH1B1; CLSTN1; UNC50; MBTPS1; PARC; RPL17; WSB1; ZNF706; SERTAD2; NARG1L; TMEM131; LOC388969; RAB1A; WASF2; TMEM59; HELZ; JMJD2B; ZDHHC7; FTL; MAPK14; MCM3AP; RHOT1; CHD9; HERC1; GDI1; CMTM6; VAV1; ZMYM1; NBPF1; ISCU; RNF111; CCNT2; ZMIZ1; FOXJ3; ZDHHC17; FNTA; MECP2; ST13; FOXN3; UBR4; FTH1; GPBP1L1; TMEM111; NT5C2; SMCHD1; SNX1; REL; MRFAP1L1; FRYL; MAP4K3; DPYSL2; GAK; BICD2; FKBP1A; MAGED2; KIDINS220; LRRC47; MYT1; ACTR10; GGNBP2; OSBPL2; MAPRE2; ROCK1; WDR37; NISCH; NUDT9; ZKSCAN1; UBE2Z; ZFAND6; SFRS5; RNASEN; TINAGL1; SLC22A5; ADCK2; PLAC4; NEBL; BSPRY; ZNF528; ATP1A3; TAT; NIP7; KMO; COL1A1; C6orf60; NCF1; ANKRD55; VIPR2; RGS5; UCK2; CDC14B; E2F2; SEMA6D; KCTD20; MUC5AC; MARCH3; TPR; PLGLB2; TERT; NR1D2; GINS4; ENTPD3; XYLT1; BCAS4; PRL; CLEC2D; SPPL2B; SORT1; PCSK6; RNF144A; CPM; EZR; MLPH; C6orf124; ADK; KCNJ2; BASP1; KCNJ3; RHOQ; IL8; CD84; FERMT1; CD48; CDKN2D; SEMA5A; MDM4; PTTG3; CXorf21; CD72; EIF2C3; GLDC; TRAK2; RBBP5; PLGLB1; KRAS; ADAM19; TRIM16; C1orf135; NFATC4; ARHGAP8; ST6GALNAC4; ZNF124; ARHGEF11; CSRP1; ZNF385D; TCF3; SPAG6; JAM2; IGHM; GATM; WASF1; SH3PXD2A; PPFIBP1; TNK2; BIK; SYNJ2; FAM152A; VDR; TMSL8; NRGN; PRR5; PPFIA4; ARNTL2; SRGAP2; MAP3K1; PHACTR1; EWSR1; F13A1; CASC1; TRIB2; MAP1B; FGF9; GPR176; PSAT1; PIGZ; SLC15A2; ENDOD1; GNAZ; KCNA3; ALDH1A1; GALNT14; SYT1; TMEM121; RASAL1; CCDC81; IL12RB2; APBB2; EAF2; ELL3; BLK; IRF4; KIAA1305; AOX1; ROR1; SAMD4A; LRMP; KANK1; ADARB1; DACT1; KCNJ12; NP; FHOD3; SLAMF1; GP5; KIAA0802; SYNPO; PSEN2; SLC27A2; MERTK; PBX1 |
| Normal | HAT1 | <0.001 | 0 | - | down | 500 | 64 | PSMA7; MRPL34; TRMT5; NDUFB2; COPS3; COX6C; RBX1; HMGN2; AKR7A2; PSMB3; ATP5J; COPS6; UCRC; TIMM8B; HMGN3; MCM6; RPA3; RFC4; CCDC56; H2AFZ; NDUFA6; NDUFS6; RAN; COX6A1; SFRS2B; MRP63; PSMD2; MRPL13; PSMD4; MDH1; NDUFS1; PSMA3; TP53TG3; CCR5; MYLIP; APOL6; GRIK2; RASL10A; PDE8B; TOX3; KAL1; FLRT3; EFR3B; PRKG2; ZNF239; ZNF192; CSHL1; CDH11; ANGPT2; REEP1; FLT1; PCBP4; FER1L3; SLC6A16; AGMAT; TXNRD3; MPPED2; HEY2; LHFPL2; CHST7; GFOD1; STAP1; PTPRM; CENTG2 |
| Normal | MYST4 | <0.001 | 0 | - | down | 2000 | 84 | LOC552891; HPRT1; C16orf62; PRDX3; GOT1; DPYSL2; NDUFA1; PDHX; TRMT5; COX7B; FRG1; LAGE3; HMGN2; AIF1; SMS; RBBP7; PSMC5; UBE2A; ATPIF1; ATP5J; PGD; HMGN1; ARMCX1; UCRC; SOD1; BCAP31; RCOR1; HMGN3; PECI; NDUFB8; CCDC56; CD93; RWDD1; SLC9A6; PHB; PSMD4; FAM45B; PSMA3; NDUFA8; GPHN; RIMS3; TP53TG3; CCR5; APOL6; GRIK2; CBLB; DLGAP4; RASL10A; PDE8B; TOX3; KAL1; HIP1; AKAP12; EFR3B; PRKG2; ABCB4; ZNF239; PTGDR; CD84; ZNF192; APOBEC3G; CSHL1; ANGPT2; REEP1; RAG2; PRUNE2; LRRC8B; PCDH9; PCBP4; GLDC; RHBDL2; FER1L3; SLC6A16; CAPN3; AGMAT; LGR5; TXNRD3; MPPED2; LHFPL2; CHST7; CHST2; STAP1; PTPRM; CENTG2 |
| Pseudodip | MYST4 | <0.001 | 0 | - | down | 500 | 16 | BACH1; C4orf41; FAM45B; SIRT1; NUP54; RAB5A; PSMC6; LAMP2; TCEAL1; HNRPH2; ATP1A3; GPR176; PCDH9; dJ222E13.2; AEBP1; SLIT2 |
| Normal | MYST1 | <0.001 | 0 | - | down | 2000 | 81 | MRPL34; TRMT5; RBX1; PSMC5; MAGOH; PSMB3; ATPIF1; PGD; COPS6; UCRC; FLII; ENSA; RCOR1; AHSA1; HN1; NDUFB8; RFC4; CCDC56; NDUFA6; NDUFS6; GPI; ARL6IP1; RAN; COX6A1; SFRS2B; MRP63; PSMD2; U2AF1; PSMD4; MDH1; NDUFS1; PSMA3; HSPA5; NDUFA8; MCTP2; CD34; GPHN; TP53TG3; CCR5; APOL6; GRIK2; CBLB; DLGAP4; RASL10A; PDE8B; TOX3; KAL1; HIP1; AKAP12; FLRT3; EFR3B; PRKG2; ABCB4; ZNF239; CD84; ZNF192; CSHL1; RXRA; CDH11; ANGPT2; ITGA6; REEP1; FLT1; PRUNE2; PCDH9; PCBP4; GLDC; FER1L3; SLC6A16; AGMAT; LGR5; PBX2; TXNRD3; MPPED2; HEY2; LHFPL2; CHST7; GFOD1; STAP1; PTPRM; CENTG2 |
| Normal | CTCF | <0.001 | 0 | - | down | 1000 | 56 | TRMT5; COX6C; TYMS; RBX1; HMGN2; PSMC5; PSMB3; ATPIF1; HMGN1; COPS6; UCRC; TUBA1C; MCM6; RPA3; HN1; NDUFB8; RFC4; CCDC56; H2AFZ; NDUFA6; NDUFS6; ARL6IP1; RAN; COX6A1; SFRS2B; MRP63; PSMD2; TUBB2C; PSMD4; MDH1; NDUFS1; CD34; TP53TG3; CCR5; APOL6; GRIK2; DLGAP4; PDE8B; KAL1; HIP1; EFR3B; PRKG2; ZNF239; ZNF192; CSHL1; CDH11; ANGPT2; REEP1; FLT1; FER1L3; SLC6A16; AGMAT; TXNRD3; MPPED2; LHFPL2; STAP1 |
