# Supplementary material for: Mechanism-anchored profiling derived from epigenetic networks predicts outcome in acute lymphoblastic leukemia
Source: BMC Bioinformatics. 2009 Sep 17;10(Suppl 9):S6. doi: 10.1186/1471-2105-10-S9-S6 (PMC2745693; doi:10.1186/1471-2105-10-S9-S6)
Supplement: Additional file 5 — Supplementary Figure 2 – The Design of the two Evaluations (A and B). [file 1471-2105-10-S9-S6-S5.doc]

**(a)**

**(b)**

**Supplementary Figure 2**. **The Design of the Evaluations**. The 87 leukemia patients with “CCR” or “relapse” information were randomly divided into three stratified folds, two of which were used to identify the relapse associated GEMs and ESGs, and the remaining one third of samples was used as a blinded test set. Such 3-fold cross-validation (the processes in the dash box) was repeated 100 times. Panel (a) shows the evaluation with machine-learning while panel (b) without machine learning. Result from Evaluation A was shown in **Fig. 3** in the main document and in **Suppl. Fig. 4** and the result from Evaluation B was shown in **Suppl. Fig. 3**.
