# Supplementary material for: Mechanism-anchored profiling derived from epigenetic networks predicts outcome in acute lymphoblastic leukemia
Source: BMC Bioinformatics. 2009 Sep 17;10(Suppl 9):S6. doi: 10.1186/1471-2105-10-S9-S6 (PMC2745693; doi:10.1186/1471-2105-10-S9-S6)
Supplement: Additional file 6 — Supplementary Figure 3 – The ROC curve of the computational evaluation method B of PGnet-predicted GEMs and ESGs signature associated with ALL relapse. [file 1471-2105-10-S9-S6-S6.doc]

**Supplementary Figure 3**. **The ROC curve of the computational evaluation method B of PGnet-predicted GEMs and ESGs signature associated with ALL relapse**. Computational evaluation method B of PGnet-predicted GEMs and ESGs signature associated with ALL relapse. The 87 leukemia patients with “CCR” or “relapse” information were randomly divided into three stratified folds, two of which were used to identify the outcome (“CCR” vs. “relapse”) associated GEMs and ESGs, and the remaining one was used as a blinded test set using PAM. Such three-fold cross-validation was repeated 100 times. The resulting ROC curve, area under the curve and corresponding p-values were calculated by the Bioconductor package *verification*.
