# Supplementary material for: Mechanism-anchored profiling derived from epigenetic networks predicts outcome in acute lymphoblastic leukemia
Source: BMC Bioinformatics. 2009 Sep 17;10(Suppl 9):S6. doi: 10.1186/1471-2105-10-S9-S6 (PMC2745693; doi:10.1186/1471-2105-10-S9-S6)
Supplement: Additional file 7 — Supplementary Figure 4 – The precision_recall results of the computational evaluation A of PGnet-predicted GEMs and ESGs signature associated with ALL relapse. [file 1471-2105-10-S9-S6-S7.doc]

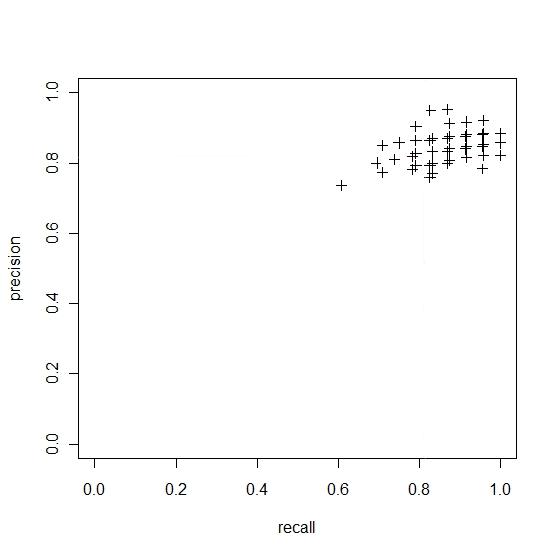

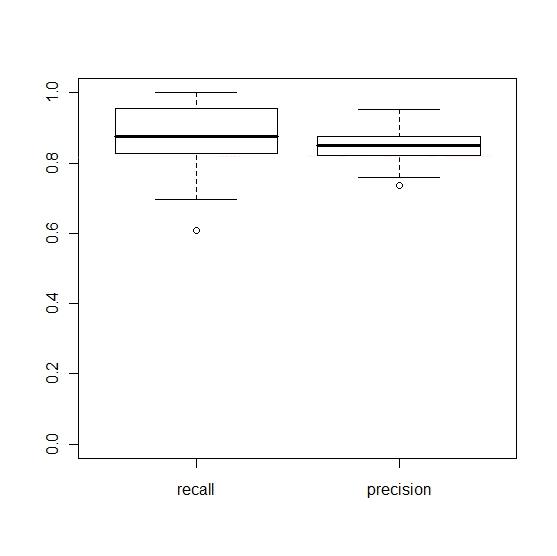


**Supplementary Figure 4**. **The precision_recall results of the computational evaluation A of PGnet-predicted GEMs and ESGs signature associated with ALL relapse**. The 87 leukemia patients with “CCR” or “relapse” information were randomly divided into three folds, two of which were used to identify the outcome (“CCR” vs. “relapse”) associated GEMs and ESGs to train a linear SVM model, and the remaining one was used as a blinded test set. Such three-fold cross-validation was repeated 100 times. The left panel is the correspondind precision-recall graph; while the right panel is the box-and-whisker plot (the smallest observation = circle, the lower quartile, median and upper quartile, and the largest observation, whiskers are the 95% and 5% intervals) showing outliers as single points for the precisions and recalls.
