# Supplementary material for: Mechanism-anchored profiling derived from epigenetic networks predicts outcome in acute lymphoblastic leukemia
Source: BMC Bioinformatics. 2009 Sep 17;10(Suppl 9):S6. doi: 10.1186/1471-2105-10-S9-S6 (PMC2745693; doi:10.1186/1471-2105-10-S9-S6)
Supplement: Additional file 8 — Supplementary Table 5 – Robust GEMs and ESGs Predictive of Leukemia Relapse. [file 1471-2105-10-S9-S6-S8.doc]

**Supplementary Table 5**

**Robust GEMs and ESGs Predictive of Leukemia Relapse**

- The robust 52 GEMs and 3 ESGs associating predictive of leukemia “relapse” that were identified with frequencies of occurrence of 95% or above in the 100 iterations of the three-fold cross-validation (**Methods**).

| **Probe** | **Symbol** | **Description** | **GenBank** | **UniGene** |
| --- | --- | --- | --- | --- |
| [201105_at](https://www.affymetrix.com/LinkServlet?&probeset=201105_at) | LGALS1 | lectin, galactoside-binding, soluble, 1 (galectin 1) | [NM_002305](http://www.ncbi.nlm.nih.gov/entrez/query.fcgi?cmd=search&db=nucleotide&term=NM_002305%5BACCN%5D&doptcmdl=GenBank) | [Hs.445351](http://www.ncbi.nlm.nih.gov/UniGene/clust.cgi?ORG=Hs&CID=445351) |
| [201163_s_at](https://www.affymetrix.com/LinkServlet?&probeset=201163_s_at) | IGFBP7 | insulin-like growth factor binding protein 7 | [NM_001553](http://www.ncbi.nlm.nih.gov/entrez/query.fcgi?cmd=search&db=nucleotide&term=NM_001553%5BACCN%5D&doptcmdl=GenBank) | [Hs.479808](http://www.ncbi.nlm.nih.gov/UniGene/clust.cgi?ORG=Hs&CID=479808) |
| [201292_at](https://www.affymetrix.com/LinkServlet?&probeset=201292_at) | TOP2A | topoisomerase (DNA) II alpha 170kDa | [AL561834](http://www.ncbi.nlm.nih.gov/entrez/query.fcgi?cmd=search&db=nucleotide&term=AL561834%5BACCN%5D&doptcmdl=GenBank) | [Hs.156346](http://www.ncbi.nlm.nih.gov/UniGene/clust.cgi?ORG=Hs&CID=156346) |
| [202377_at](https://www.affymetrix.com/LinkServlet?&probeset=202377_at) | LEPROT | leptin receptor overlapping transcript | [AW026535](http://www.ncbi.nlm.nih.gov/entrez/query.fcgi?cmd=search&db=nucleotide&term=AW026535%5BACCN%5D&doptcmdl=GenBank) | [Hs.705413](http://www.ncbi.nlm.nih.gov/UniGene/clust.cgi?ORG=Hs&CID=705413) |
| [202626_s_at](https://www.affymetrix.com/LinkServlet?&probeset=202626_s_at) | LYN | v-yes-1 Yamaguchi sarcoma viral related oncogene homolog | [NM_002350](http://www.ncbi.nlm.nih.gov/entrez/query.fcgi?cmd=search&db=nucleotide&term=NM_002350%5BACCN%5D&doptcmdl=GenBank) | [Hs.699154](http://www.ncbi.nlm.nih.gov/UniGene/clust.cgi?ORG=Hs&CID=699154) |
| [202705_at](https://www.affymetrix.com/LinkServlet?&probeset=202705_at) | CCNB2 | cyclin B2 | [NM_004701](http://www.ncbi.nlm.nih.gov/entrez/query.fcgi?cmd=search&db=nucleotide&term=NM_004701%5BACCN%5D&doptcmdl=GenBank) | [Hs.194698](http://www.ncbi.nlm.nih.gov/UniGene/clust.cgi?ORG=Hs&CID=194698) |
| [202748_at](https://www.affymetrix.com/LinkServlet?&probeset=202748_at) | GBP2 | guanylate binding protein 2, interferon-inducible | [NM_004120](http://www.ncbi.nlm.nih.gov/entrez/query.fcgi?cmd=search&db=nucleotide&term=NM_004120%5BACCN%5D&doptcmdl=GenBank) | [Hs.386567](http://www.ncbi.nlm.nih.gov/UniGene/clust.cgi?ORG=Hs&CID=386567) |
| [202934_at](https://www.affymetrix.com/LinkServlet?&probeset=202934_at) | HK2 | hexokinase 2 | [AI761561](http://www.ncbi.nlm.nih.gov/entrez/query.fcgi?cmd=search&db=nucleotide&term=AI761561%5BACCN%5D&doptcmdl=GenBank) | [Hs.406266](http://www.ncbi.nlm.nih.gov/UniGene/clust.cgi?ORG=Hs&CID=406266) [Hs.591588](http://www.ncbi.nlm.nih.gov/UniGene/clust.cgi?ORG=Hs&CID=591588) |
| [203005_at](https://www.affymetrix.com/LinkServlet?&probeset=203005_at) | LTBR | lymphotoxin beta receptor (TNFR superfamily, member 3) | [NM_002342](http://www.ncbi.nlm.nih.gov/entrez/query.fcgi?cmd=search&db=nucleotide&term=NM_002342%5BACCN%5D&doptcmdl=GenBank) | [Hs.1116](http://www.ncbi.nlm.nih.gov/UniGene/clust.cgi?ORG=Hs&CID=1116) |
| [203045_at](https://www.affymetrix.com/LinkServlet?&probeset=203045_at) | NINJ1 | ninjurin 1 | [NM_004148](http://www.ncbi.nlm.nih.gov/entrez/query.fcgi?cmd=search&db=nucleotide&term=NM_004148%5BACCN%5D&doptcmdl=GenBank) | [Hs.494457](http://www.ncbi.nlm.nih.gov/UniGene/clust.cgi?ORG=Hs&CID=494457) |
| [203145_at](https://www.affymetrix.com/LinkServlet?&probeset=203145_at) | SPAG5 | sperm associated antigen 5 | [NM_006461](http://www.ncbi.nlm.nih.gov/entrez/query.fcgi?cmd=search&db=nucleotide&term=NM_006461%5BACCN%5D&doptcmdl=GenBank) | [Hs.514033](http://www.ncbi.nlm.nih.gov/UniGene/clust.cgi?ORG=Hs&CID=514033) |
| [203186_s_at](https://www.affymetrix.com/LinkServlet?&probeset=203186_s_at) | S100A4 | S100 calcium binding protein A4 | [NM_002961](http://www.ncbi.nlm.nih.gov/entrez/query.fcgi?cmd=search&db=nucleotide&term=NM_002961%5BACCN%5D&doptcmdl=GenBank) | [Hs.654444](http://www.ncbi.nlm.nih.gov/UniGene/clust.cgi?ORG=Hs&CID=654444) |
| [203362_s_at](https://www.affymetrix.com/LinkServlet?&probeset=203362_s_at) | MAD2L1 | MAD2 mitotic arrest deficient-like 1 (yeast) | [NM_002358](http://www.ncbi.nlm.nih.gov/entrez/query.fcgi?cmd=search&db=nucleotide&term=NM_002358%5BACCN%5D&doptcmdl=GenBank) | [Hs.591697](http://www.ncbi.nlm.nih.gov/UniGene/clust.cgi?ORG=Hs&CID=591697) |
| [203434_s_at](https://www.affymetrix.com/LinkServlet?&probeset=203434_s_at) | MME | membrane metallo-endopeptidase | [AI433463](http://www.ncbi.nlm.nih.gov/entrez/query.fcgi?cmd=search&db=nucleotide&term=AI433463%5BACCN%5D&doptcmdl=GenBank) | [Hs.307734](http://www.ncbi.nlm.nih.gov/UniGene/clust.cgi?ORG=Hs&CID=307734) |
| [204026_s_at](https://www.affymetrix.com/LinkServlet?&probeset=204026_s_at) | ZWINT | ZW10 interactor | [NM_007057](http://www.ncbi.nlm.nih.gov/entrez/query.fcgi?cmd=search&db=nucleotide&term=NM_007057%5BACCN%5D&doptcmdl=GenBank) | [Hs.591363](http://www.ncbi.nlm.nih.gov/UniGene/clust.cgi?ORG=Hs&CID=591363) |
| [204126_s_at](https://www.affymetrix.com/LinkServlet?&probeset=204126_s_at) | CDC45L | CDC45 cell division cycle 45-like (S. cerevisiae) | [NM_003504](http://www.ncbi.nlm.nih.gov/entrez/query.fcgi?cmd=search&db=nucleotide&term=NM_003504%5BACCN%5D&doptcmdl=GenBank) | [Hs.474217](http://www.ncbi.nlm.nih.gov/UniGene/clust.cgi?ORG=Hs&CID=474217) |
| [204444_at](https://www.affymetrix.com/LinkServlet?&probeset=204444_at) | KIF11 | kinesin family member 11 | [NM_004523](http://www.ncbi.nlm.nih.gov/entrez/query.fcgi?cmd=search&db=nucleotide&term=NM_004523%5BACCN%5D&doptcmdl=GenBank) | [Hs.8878](http://www.ncbi.nlm.nih.gov/UniGene/clust.cgi?ORG=Hs&CID=8878) |
| [208939_at](https://www.affymetrix.com/LinkServlet?&probeset=208939_at) | SEPHS1 | selenophosphate synthetase 1 | [AV682679](http://www.ncbi.nlm.nih.gov/entrez/query.fcgi?cmd=search&db=nucleotide&term=AV682679%5BACCN%5D&doptcmdl=GenBank) | [Hs.124027](http://www.ncbi.nlm.nih.gov/UniGene/clust.cgi?ORG=Hs&CID=124027) |
| [208950_s_at](https://www.affymetrix.com/LinkServlet?&probeset=208950_s_at) | ALDH7A1 | aldehyde dehydrogenase 7 family, member A1 | [BC002515](http://www.ncbi.nlm.nih.gov/entrez/query.fcgi?cmd=search&db=nucleotide&term=BC002515%5BACCN%5D&doptcmdl=GenBank) | [Hs.483239](http://www.ncbi.nlm.nih.gov/UniGene/clust.cgi?ORG=Hs&CID=483239) |
| [209026_x_at](https://www.affymetrix.com/LinkServlet?&probeset=209026_x_at) | TUBB | tubulin, beta | [AF141349](http://www.ncbi.nlm.nih.gov/entrez/query.fcgi?cmd=search&db=nucleotide&term=AF141349%5BACCN%5D&doptcmdl=GenBank) | [Hs.636480](http://www.ncbi.nlm.nih.gov/UniGene/clust.cgi?ORG=Hs&CID=636480) [Hs.706772](http://www.ncbi.nlm.nih.gov/UniGene/clust.cgi?ORG=Hs&CID=706772) |
| [209642_at](https://www.affymetrix.com/LinkServlet?&probeset=209642_at) | BUB1 | BUB1 budding uninhibited by benzimidazoles 1 homolog (yeast) | [AF043294](http://www.ncbi.nlm.nih.gov/entrez/query.fcgi?cmd=search&db=nucleotide&term=AF043294%5BACCN%5D&doptcmdl=GenBank) | [Hs.469649](http://www.ncbi.nlm.nih.gov/UniGene/clust.cgi?ORG=Hs&CID=469649) |
| [209715_at](https://www.affymetrix.com/LinkServlet?&probeset=209715_at) | CBX5 | chromobox homolog 5 (HP1 alpha homolog, Drosophila) | [L07515](http://www.ncbi.nlm.nih.gov/entrez/query.fcgi?cmd=search&db=nucleotide&term=L07515%5BACCN%5D&doptcmdl=GenBank) | [Hs.632724](http://www.ncbi.nlm.nih.gov/UniGene/clust.cgi?ORG=Hs&CID=632724) |
| [210052_s_at](https://www.affymetrix.com/LinkServlet?&probeset=210052_s_at) | TPX2 | TPX2, microtubule-associated, homolog (Xenopus laevis) | [AF098158](http://www.ncbi.nlm.nih.gov/entrez/query.fcgi?cmd=search&db=nucleotide&term=AF098158%5BACCN%5D&doptcmdl=GenBank) | [Hs.244580](http://www.ncbi.nlm.nih.gov/UniGene/clust.cgi?ORG=Hs&CID=244580) |
| [212021_s_at](https://www.affymetrix.com/LinkServlet?&probeset=212021_s_at) | MKI67 | antigen identified by monoclonal antibody Ki-67 | [AU132185](http://www.ncbi.nlm.nih.gov/entrez/query.fcgi?cmd=search&db=nucleotide&term=AU132185%5BACCN%5D&doptcmdl=GenBank) | [Hs.80976](http://www.ncbi.nlm.nih.gov/UniGene/clust.cgi?ORG=Hs&CID=80976) |
| [212372_at](https://www.affymetrix.com/LinkServlet?&probeset=212372_at) | MYH10 | myosin, heavy chain 10, non-muscle | [AK026977](http://www.ncbi.nlm.nih.gov/entrez/query.fcgi?cmd=search&db=nucleotide&term=AK026977%5BACCN%5D&doptcmdl=GenBank) | [Hs.16355](http://www.ncbi.nlm.nih.gov/UniGene/clust.cgi?ORG=Hs&CID=16355) |
| [212386_at](https://www.affymetrix.com/LinkServlet?&probeset=212386_at) | TCF4 | transcription factor 4 | [BF592782](http://www.ncbi.nlm.nih.gov/entrez/query.fcgi?cmd=search&db=nucleotide&term=BF592782%5BACCN%5D&doptcmdl=GenBank) | [Hs.644653](http://www.ncbi.nlm.nih.gov/UniGene/clust.cgi?ORG=Hs&CID=644653) |
| [212488_at](https://www.affymetrix.com/LinkServlet?&probeset=212488_at) | COL5A1 | collagen, type V, alpha 1 | [N30339](http://www.ncbi.nlm.nih.gov/entrez/query.fcgi?cmd=search&db=nucleotide&term=N30339%5BACCN%5D&doptcmdl=GenBank) | [Hs.210283](http://www.ncbi.nlm.nih.gov/UniGene/clust.cgi?ORG=Hs&CID=210283) |
| [212688_at](https://www.affymetrix.com/LinkServlet?&probeset=212688_at) | PIK3CB | phosphoinositide-3-kinase, catalytic, beta polypeptide | [BC003393](http://www.ncbi.nlm.nih.gov/entrez/query.fcgi?cmd=search&db=nucleotide&term=BC003393%5BACCN%5D&doptcmdl=GenBank) | [Hs.239818](http://www.ncbi.nlm.nih.gov/UniGene/clust.cgi?ORG=Hs&CID=239818) |
| [212949_at](https://www.affymetrix.com/LinkServlet?&probeset=212949_at) | NCAPH | non-SMC condensin I complex, subunit H | [D38553](http://www.ncbi.nlm.nih.gov/entrez/query.fcgi?cmd=search&db=nucleotide&term=D38553%5BACCN%5D&doptcmdl=GenBank) | [Hs.308045](http://www.ncbi.nlm.nih.gov/UniGene/clust.cgi?ORG=Hs&CID=308045) |
| [213222_at](https://www.affymetrix.com/LinkServlet?&probeset=213222_at) | PLCB1 | phospholipase C, beta 1 (phosphoinositide-specific) | [AL049593](http://www.ncbi.nlm.nih.gov/entrez/query.fcgi?cmd=search&db=nucleotide&term=AL049593%5BACCN%5D&doptcmdl=GenBank) | [Hs.431173](http://www.ncbi.nlm.nih.gov/UniGene/clust.cgi?ORG=Hs&CID=431173) |
| [213241_at](https://www.affymetrix.com/LinkServlet?&probeset=213241_at) | PLXNC1 | plexin C1 | [AF035307](http://www.ncbi.nlm.nih.gov/entrez/query.fcgi?cmd=search&db=nucleotide&term=AF035307%5BACCN%5D&doptcmdl=GenBank) | [Hs.584845](http://www.ncbi.nlm.nih.gov/UniGene/clust.cgi?ORG=Hs&CID=584845) |
| [213283_s_at](https://www.affymetrix.com/LinkServlet?&probeset=213283_s_at) | SALL2 | sal-like 2 (Drosophila) | [BG285616](http://www.ncbi.nlm.nih.gov/entrez/query.fcgi?cmd=search&db=nucleotide&term=BG285616%5BACCN%5D&doptcmdl=GenBank) | [Hs.416358](http://www.ncbi.nlm.nih.gov/UniGene/clust.cgi?ORG=Hs&CID=416358) |
| [213737_x_at](https://www.affymetrix.com/LinkServlet?&probeset=213737_x_at) | GOLGA8G | golgi autoantigen, golgin subfamily a, 8G | [AI620911](http://www.ncbi.nlm.nih.gov/entrez/query.fcgi?cmd=search&db=nucleotide&term=AI620911%5BACCN%5D&doptcmdl=GenBank) | [Hs.169639](http://www.ncbi.nlm.nih.gov/UniGene/clust.cgi?ORG=Hs&CID=169639) [Hs.510812](http://www.ncbi.nlm.nih.gov/UniGene/clust.cgi?ORG=Hs&CID=510812) [Hs.525714](http://www.ncbi.nlm.nih.gov/UniGene/clust.cgi?ORG=Hs&CID=525714) |
| [213836_s_at](https://www.affymetrix.com/LinkServlet?&probeset=213836_s_at) | WIPI1 | WD repeat domain, phosphoinositide interacting 1 | [AW052084](http://www.ncbi.nlm.nih.gov/entrez/query.fcgi?cmd=search&db=nucleotide&term=AW052084%5BACCN%5D&doptcmdl=GenBank) | [Hs.463964](http://www.ncbi.nlm.nih.gov/UniGene/clust.cgi?ORG=Hs&CID=463964) |
| [213911_s_at](https://www.affymetrix.com/LinkServlet?&probeset=213911_s_at) | H2AFZ | H2A histone family, member Z | [BF718636](http://www.ncbi.nlm.nih.gov/entrez/query.fcgi?cmd=search&db=nucleotide&term=BF718636%5BACCN%5D&doptcmdl=GenBank) | [Hs.119192](http://www.ncbi.nlm.nih.gov/UniGene/clust.cgi?ORG=Hs&CID=119192) |
| [215000_s_at](https://www.affymetrix.com/LinkServlet?&probeset=215000_s_at) | FEZ2 | fasciculation and elongation protein zeta 2 (zygin II) | [AL117593](http://www.ncbi.nlm.nih.gov/entrez/query.fcgi?cmd=search&db=nucleotide&term=AL117593%5BACCN%5D&doptcmdl=GenBank) | [Hs.258563](http://www.ncbi.nlm.nih.gov/UniGene/clust.cgi?ORG=Hs&CID=258563) |
| [215221_at](https://www.affymetrix.com/LinkServlet?&probeset=215221_at) | FOXP1 | forkhead box P1 | [AK025064](http://www.ncbi.nlm.nih.gov/entrez/query.fcgi?cmd=search&db=nucleotide&term=AK025064%5BACCN%5D&doptcmdl=GenBank) | [Hs.431498](http://www.ncbi.nlm.nih.gov/UniGene/clust.cgi?ORG=Hs&CID=431498) |
| [215239_x_at](https://www.affymetrix.com/LinkServlet?&probeset=215239_x_at) | ZNF273 | zinc finger protein 273 | [AU132789](http://www.ncbi.nlm.nih.gov/entrez/query.fcgi?cmd=search&db=nucleotide&term=AU132789%5BACCN%5D&doptcmdl=GenBank) | [Hs.520889](http://www.ncbi.nlm.nih.gov/UniGene/clust.cgi?ORG=Hs&CID=520889) |
| [215714_s_at](https://www.affymetrix.com/LinkServlet?&probeset=215714_s_at) | SMARCA4 | SWI/SNF related, matrix associated, actin dependent regulator of chromatin, subfamily a, member 4 | [AF254822](http://www.ncbi.nlm.nih.gov/entrez/query.fcgi?cmd=search&db=nucleotide&term=AF254822%5BACCN%5D&doptcmdl=GenBank) | [Hs.327527](http://www.ncbi.nlm.nih.gov/UniGene/clust.cgi?ORG=Hs&CID=327527) |
| [216026_s_at](https://www.affymetrix.com/LinkServlet?&probeset=216026_s_at) | POLE | polymerase (DNA directed), epsilon | [AL080203](http://www.ncbi.nlm.nih.gov/entrez/query.fcgi?cmd=search&db=nucleotide&term=AL080203%5BACCN%5D&doptcmdl=GenBank) | [Hs.524871](http://www.ncbi.nlm.nih.gov/UniGene/clust.cgi?ORG=Hs&CID=524871) [Hs.657680](http://www.ncbi.nlm.nih.gov/UniGene/clust.cgi?ORG=Hs&CID=657680) |
| [216548_x_at](https://www.affymetrix.com/LinkServlet?&probeset=216548_x_at) | HMG4L | high-mobility group (nonhistone chromosomal) protein 4-like | [AL049709](http://www.ncbi.nlm.nih.gov/entrez/query.fcgi?cmd=search&db=nucleotide&term=AL049709%5BACCN%5D&doptcmdl=GenBank) | [Hs.558624](http://www.ncbi.nlm.nih.gov/UniGene/clust.cgi?ORG=Hs&CID=558624) |
| [217025_s_at](https://www.affymetrix.com/LinkServlet?&probeset=217025_s_at) | DBN1 | drebrin 1 | [AL110225](http://www.ncbi.nlm.nih.gov/entrez/query.fcgi?cmd=search&db=nucleotide&term=AL110225%5BACCN%5D&doptcmdl=GenBank) | [Hs.130316](http://www.ncbi.nlm.nih.gov/UniGene/clust.cgi?ORG=Hs&CID=130316) |
| [217028_at](https://www.affymetrix.com/LinkServlet?&probeset=217028_at) | CXCR4 | chemokine (C-X-C motif) receptor 4 | [AJ224869](http://www.ncbi.nlm.nih.gov/entrez/query.fcgi?cmd=search&db=nucleotide&term=AJ224869%5BACCN%5D&doptcmdl=GenBank) | [Hs.593413](http://www.ncbi.nlm.nih.gov/UniGene/clust.cgi?ORG=Hs&CID=593413) |
| [217547_x_at](https://www.affymetrix.com/LinkServlet?&probeset=217547_x_at) | ZNF675 | zinc finger protein 675 | [BF308250](http://www.ncbi.nlm.nih.gov/entrez/query.fcgi?cmd=search&db=nucleotide&term=BF308250%5BACCN%5D&doptcmdl=GenBank) | [Hs.264345](http://www.ncbi.nlm.nih.gov/UniGene/clust.cgi?ORG=Hs&CID=264345) |
| [218355_at](https://www.affymetrix.com/LinkServlet?&probeset=218355_at) | KIF4A | kinesin family member 4A | [NM_012310](http://www.ncbi.nlm.nih.gov/entrez/query.fcgi?cmd=search&db=nucleotide&term=NM_012310%5BACCN%5D&doptcmdl=GenBank) | [Hs.648326](http://www.ncbi.nlm.nih.gov/UniGene/clust.cgi?ORG=Hs&CID=648326) |
| [218457_s_at](https://www.affymetrix.com/LinkServlet?&probeset=218457_s_at) | DNMT3A | DNA (cytosine-5-)-methyltransferase 3 alpha | [NM_022552](http://www.ncbi.nlm.nih.gov/entrez/query.fcgi?cmd=search&db=nucleotide&term=NM_022552%5BACCN%5D&doptcmdl=GenBank) | [Hs.515840](http://www.ncbi.nlm.nih.gov/UniGene/clust.cgi?ORG=Hs&CID=515840) |
| [218755_at](https://www.affymetrix.com/LinkServlet?&probeset=218755_at) | KIF20A | kinesin family member 20A | [NM_005733](http://www.ncbi.nlm.nih.gov/entrez/query.fcgi?cmd=search&db=nucleotide&term=NM_005733%5BACCN%5D&doptcmdl=GenBank) | [Hs.73625](http://www.ncbi.nlm.nih.gov/UniGene/clust.cgi?ORG=Hs&CID=73625) |
| [219036_at](https://www.affymetrix.com/LinkServlet?&probeset=219036_at) | CEP70 | centrosomal protein 70kDa | [NM_024491](http://www.ncbi.nlm.nih.gov/entrez/query.fcgi?cmd=search&db=nucleotide&term=NM_024491%5BACCN%5D&doptcmdl=GenBank) | [Hs.531962](http://www.ncbi.nlm.nih.gov/UniGene/clust.cgi?ORG=Hs&CID=531962) |
| [219493_at](https://www.affymetrix.com/LinkServlet?&probeset=219493_at) | SHCBP1 | SHC SH2-domain binding protein 1 | [NM_024745](http://www.ncbi.nlm.nih.gov/entrez/query.fcgi?cmd=search&db=nucleotide&term=NM_024745%5BACCN%5D&doptcmdl=GenBank) | [Hs.123253](http://www.ncbi.nlm.nih.gov/UniGene/clust.cgi?ORG=Hs&CID=123253) |
| [219789_at](https://www.affymetrix.com/LinkServlet?&probeset=219789_at) | NPR3 | natriuretic peptide receptor C/guanylate cyclase C (atrionatriuretic peptide receptor C) | [AI628360](http://www.ncbi.nlm.nih.gov/entrez/query.fcgi?cmd=search&db=nucleotide&term=AI628360%5BACCN%5D&doptcmdl=GenBank) | [Hs.237028](http://www.ncbi.nlm.nih.gov/UniGene/clust.cgi?ORG=Hs&CID=237028) [Hs.619466](http://www.ncbi.nlm.nih.gov/UniGene/clust.cgi?ORG=Hs&CID=619466) |
| [219871_at](https://www.affymetrix.com/LinkServlet?&probeset=219871_at) | FLJ13197 | hypothetical FLJ13197 | [NM_024614](http://www.ncbi.nlm.nih.gov/entrez/query.fcgi?cmd=search&db=nucleotide&term=NM_024614%5BACCN%5D&doptcmdl=GenBank) | [Hs.29725](http://www.ncbi.nlm.nih.gov/UniGene/clust.cgi?ORG=Hs&CID=29725) |
| [219978_s_at](https://www.affymetrix.com/LinkServlet?&probeset=219978_s_at) | NUSAP1 | nucleolar and spindle associated protein 1 | [NM_018454](http://www.ncbi.nlm.nih.gov/entrez/query.fcgi?cmd=search&db=nucleotide&term=NM_018454%5BACCN%5D&doptcmdl=GenBank) | [Hs.615092](http://www.ncbi.nlm.nih.gov/UniGene/clust.cgi?ORG=Hs&CID=615092) |
| [220416_at](https://www.affymetrix.com/LinkServlet?&probeset=220416_at) | ATP8B4 | ATPase, class I, type 8B, member 4 | [NM_024837](http://www.ncbi.nlm.nih.gov/entrez/query.fcgi?cmd=search&db=nucleotide&term=NM_024837%5BACCN%5D&doptcmdl=GenBank) | [Hs.511311](http://www.ncbi.nlm.nih.gov/UniGene/clust.cgi?ORG=Hs&CID=511311) |
| [220560_at](https://www.affymetrix.com/LinkServlet?&probeset=220560_at) | C11orf21 | chromosome 11 open reading frame 21 | [NM_014144](http://www.ncbi.nlm.nih.gov/entrez/query.fcgi?cmd=search&db=nucleotide&term=NM_014144%5BACCN%5D&doptcmdl=GenBank) | [Hs.559181](http://www.ncbi.nlm.nih.gov/UniGene/clust.cgi?ORG=Hs&CID=559181) |
| [221505_at](https://www.affymetrix.com/LinkServlet?&probeset=221505_at) | ANP32E | acidic (leucine-rich) nuclear phosphoprotein 32 family, member E | [AW612574](http://www.ncbi.nlm.nih.gov/entrez/query.fcgi?cmd=search&db=nucleotide&term=AW612574%5BACCN%5D&doptcmdl=GenBank) | [Hs.656466](http://www.ncbi.nlm.nih.gov/UniGene/clust.cgi?ORG=Hs&CID=656466) |
