# Supplementary material for: Mechanism-anchored profiling derived from epigenetic networks predicts outcome in acute lymphoblastic leukemia
Source: BMC Bioinformatics. 2009 Sep 17;10(Suppl 9):S6. doi: 10.1186/1471-2105-10-S9-S6 (PMC2745693; doi:10.1186/1471-2105-10-S9-S6)
Supplement: Additional file 9 — Supplementary Table 4 – The significantly enriched GO items among the predicted GEMs that linking to phenotypes of interested. [file 1471-2105-10-S9-S6-S9.doc]

**Supplementary Table 4**

**The significantly enriched GO items among the predicted GEMs that linking to phenotypes of interested**

***LP:*** Leukemia Phenotype

***ESG***: Epigenetic Seed Gene

***MF***: The GO ID for molecular function

***Description***: The description of the GO ID

***#GO***: The counts of Entrez Gene ids from the Hug133a array at each GO ID.

***#Int***: The counts of the Genes co-Expressed with Mechanism genes (GEMs) annotated at each GO term.

**Un-adjusted P**: The un-adjusted empirical vectorial enrichment p-value of every significant (p<0.001) “LP-ESG” pair

***q***: The estimated false discovery rate (proportion of false positives incurred) at given empirical p-value threshold (**Suppl. Methods**).

***GEMs(200)***: The predicted GEMs using a parameter *T*=200.

| **LP-ESG** | **MF** | **Description** | **#GO** | **#Int** | **Un-adjusted P** | **q** | **GEMs(200)** |
| --- | --- | --- | --- | --- | --- | --- | --- |
| T-ALL~DNMT3B  T-ALL~HDAC4  BCR-ABL~DNMT3B  BCR-ABL~HDAC4 | GO:0032395 | MHC class II receptor activity | 12 | 6 | 1.6e-11 | 1.8e-9 | CD74**;** HLA-DRA**;** HLA-DRB1**;** BLNK**;** HLA-DPA1**;** HLA-DPB1**;** HLA-DMA**;** HLA-DRB5**;** CD79B**;** MEF2C**;** TCL1A**;** CD79A**;** SNX2**;** JUP**;** POU2AF1**;** TFEB**;** CHD7**;** GALNAC4S-6ST**;** HLA-DQB1**;** LAMC1**;** STX7**;** SLC27A3**;** PTPN18**;** HLA-DMB**;** BANK1**;** HLA-DRB6**;** PTK2**;** ENG**;** tcag7.1314**;** HLA-F**;** FHL1**;** STK32B**;** SLC25A15**;** INSIG1**;** DSTN**;** HIRA**;** ZAP70**;** NOTCH3**;** MLLT11**;** C9orf78**;** VAT1**;** PELO**;** C14orf135**;** LZTFL1**;** CD247**;**  NUCB2**;** PEX5**;** CHI3L2**;** NBR1**;** USP20**;** LAT**;** BCL11B**;** LCK**;** UBASH3A**;** MAL**;**  CD3D**;** C5orf13**;** GLUL**;** JARID1B**;** MVP**;** STX3**;** GIMAP4**;** S100A13**;** MS4A1**;** RAPGEF3**;**  TBXA2R**;** ECM1 |
| Relapse~CBX5  Relapse~DNMT3A  Relapse~HDAC9  Relapse~ SUV39H1 | GO:0005524 | ATP binding | 1067 | 14 | 3.5e-6 | 0.0002 | TUBB**;** NCAPH**;** TOP2A**;** MKI67**;** TPX2**;** ANP32E**;** KIF20A**;** CCNB2**;** BUB1**;** CCNA2**;** AURKB**;** KIF2C**;** H2AFZ**;** CKS1B**;** ZWINT**;** NPR3**;** FLJ13197**;** SHCBP1**;** ARHGAP19**;** KIF4A**;** SPAG5**;** CDC45L**;** C21orf45**;** BIRC5**;** KIF11**;** PLK4**;** TIMELESS**;** MAD2L1**;** RAD51**;** DBN1**;** SEPHS1**;** ZNF675**;** PRPF4B**;** S100A4**;** MYH10**;** SALL2**;** CEBPE**;** LGALS1**;** IGFBP7 |
|  | GO:0032559 | adenyl ribonucleotide binding | 1080 | 14 | 4.1e-6 | 0.0002 |  |
|  | GO:0030554 | adenyl nucleotide binding | 1139 | 14 | 7.7e-6 | 0.0002 |  |
|  | GO:0032553 | ribonucleotide binding | 1328 | 15 | 8.9e-6 | 0.0002 |  |
|  | GO:0032555 | purine ribonucleotide binding | 1328 | 15 | 8.9e-6 | 0.0002 |  |
|  | GO:0017076 | purine nucleotide binding | 1389 | 15 | 1.5e-5 | 0.0003 |  |
|  | GO:0003774 | motor activity | 109 | 5 | 2.8e-5 | 0.0003 |  |
|  | GO:0003777 | microtubule motor activity | 54 | 4 | 2.9e-5 | 0.0003 |  |
|  | GO:0000166 | nucleotide binding | 1596 | 15 | 8.2e-5 | 0.0008 |  |
| Hyperdip>50**~**BAZ2A Hyperdip>50**~**CBX7  Hyperdip>50**~**HDAC6  Hyperdip>50**~**SMARCA4 | GO:0004896 | cytokine receptor activity | 51 | 3 | 0.0019 | 0.0546 | ELF1; ZNF75; ARMCX5; ASB9; ENOX2; UBE2A; ATP6AP2; MGC39900; MORC3; HNRPH2; PRKAR2B; WDR44; SOD1; SLC9A6; SETD3; FTSJ1; ARMCX1; MTCP1; PIGP; LOC57228; DHRS4; RP6-213H19.1; CRYZL1; PSMD10; TCEAL1; TCEAL4; UPF3B; ECHDC3; ITSN1; MYBPC2; C10orf56; IL13RA1; IL3RA; ZNF185; RAG2; ABCC4; AKAP12; PQBP1; CXorf45; ABCD4; MAGEH1; LAS1L; RRP1B; GPKOW; ABCB7; UBQLN2; USP9X; RNF113A; MED12; COMMD4; ANP32A; BCL2L1; SMARCA4; SPTBN1; PSME4; HDGF; TLR2; IL6R; ALDH3B1; MS4A6A; PLP2 |
|  | GO:0001875 | lipopolysaccharide receptor activity | 1 | 1 | 0.0049 | 0.0546 |  |
|  | GO:0051076 | Gram-positive bacterium binding | 1 | 1 | 0.0049 | 0.0546 |  |
|  | GO:0019955 | cytokine binding | 81 | 3 | 0.0072 | 0.0546 |  |
|  | GO:0004907 | cytokine receptor activity | 30 | 2 | 0.0094 | 0.0546 |  |
|  | GO:0001530 | lipopolysaccharide binding | 2 | 1 | 0.0098 | 0.0546 |  |
|  | GO:0003960 | NADPH:quinone reductase activity | 2 | 1 | 0.0098 | 0.0546 |  |
|  | GO:0004912 | interleukin-3 receptor activity | 2 | 1 | 0.0098 | 0.0546 |  |
|  | GO:0004915 | interleukin-6 receptor activity | 2 | 1 | 0.0098 | 0.0546 |  |
|  | GO:0015232 | heme transporter activity | 2 | 1 | 0.0098 | 0.0546 |  |
|  | GO:0016404 | 15-hydroxyprostaglandin dehydrogenase (NAD+) activity | 2 | 1 | 0.0098 | 0.0546 |  |
|  | GO:0019978 | interleukin-3 binding | 2 | 1 | 0.0098 | 0.0546 |  |
|  | GO:0019981 | interleukin-6 binding | 2 | 1 | 0.0098 | 0.0546 |  |
