# Supplementary material for: Mechanism-anchored profiling derived from epigenetic networks predicts outcome in acute lymphoblastic leukemia
Source: BMC Bioinformatics. 2009 Sep 17;10(Suppl 9):S6. doi: 10.1186/1471-2105-10-S9-S6 (PMC2745693; doi:10.1186/1471-2105-10-S9-S6)
Supplement: Additional file 10 — Supplementary file Figure 5 – Expression pattern of ALL phenotype "Hyperdiploid>50" specific GEMs by PGnet. [file 1471-2105-10-S9-S6-S10.doc]

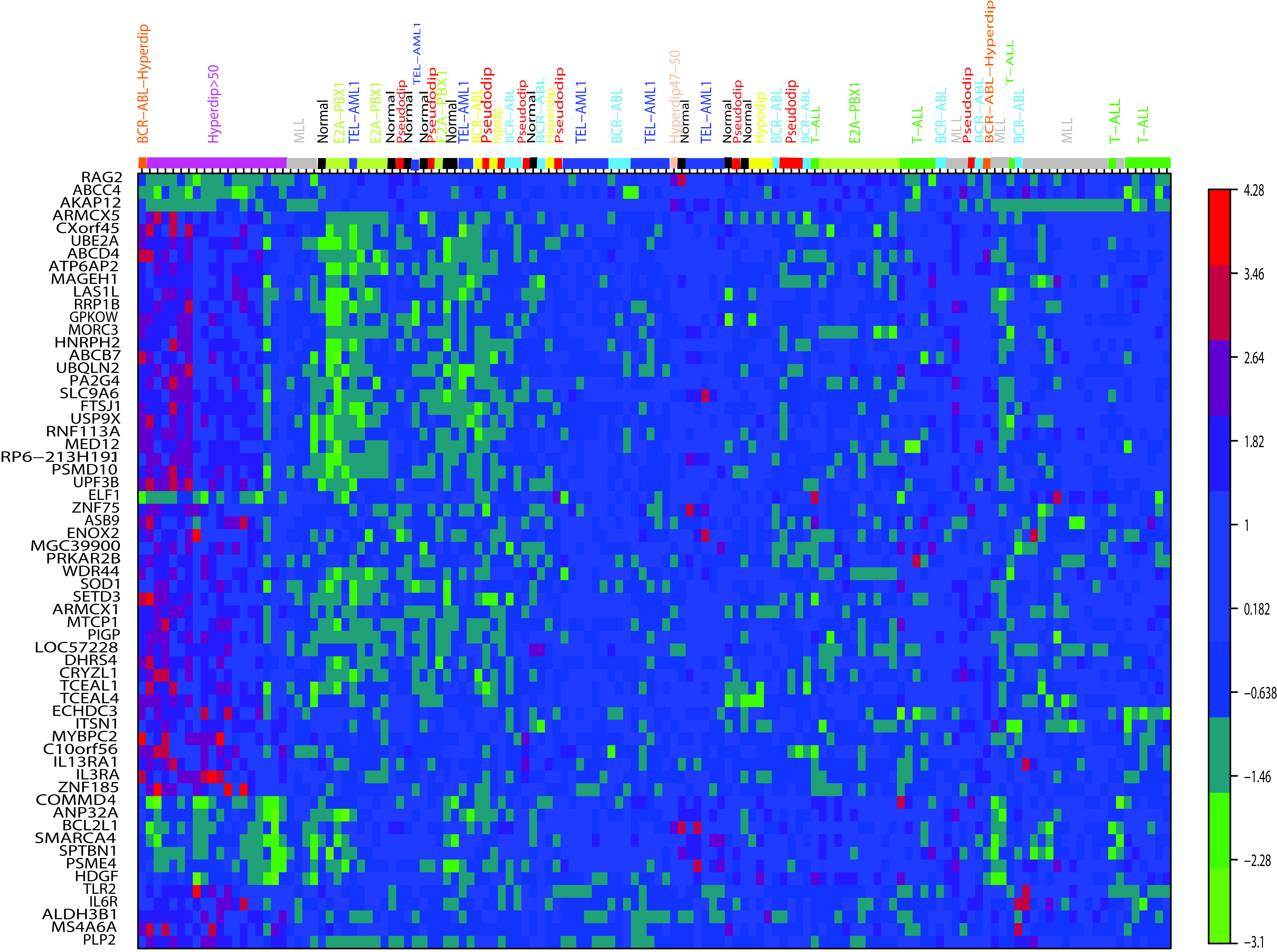


**Supplementary Figure 5. Expression pattern of ALL phenotype “Hyperdiploid>50” specific and co-expressed with known ESGs genes (GEMs) by PGnet.** Four ESGs were associated to this pattern as shown in **Figure 2** in the main document.
