# Supplementary material for: Mechanism-anchored profiling derived from epigenetic networks predicts outcome in acute lymphoblastic leukemia
Source: BMC Bioinformatics. 2009 Sep 17;10(Suppl 9):S6. doi: 10.1186/1471-2105-10-S9-S6 (PMC2745693; doi:10.1186/1471-2105-10-S9-S6)
Supplement: Additional file 11 — Supplementary Table 6 – Previously reported facts about predicted GEMs. [file 1471-2105-10-S9-S6-S11.doc]

**Supplementary Table 6**

**Previously reported facts about predicted GEMs**

- The overlap of our predicted GEMs with reported marker genes for leukemia “Hyperdip>50” (Ross, Zhou et al. 2003) was tested in the first three rows.
- The following rows in the table list the details of the corresponding GEMs.

***Probe-sets:*** The probe-set ID that Affymetrix used for Hgu133a array

***Symbol:*** Symbol of gene

***Description:*** Description of gene

***UniGene:*** Unigene ID of gene

***ESG.dys***: The average change of ESG in expression to “Hyperdip>50” compared to other phenotypes.

***HDAC6***: “Y” annotates the GEMs that associate to “HDAC6-Hyperdip>50” linkage as well were reported by Ross et al as Hyperdip>50 markers.

***BAZ2A***: “Y” annotates the GEMs that associate to “BAZ2A-Hyperdip>50” linkage as well were reported by Ross et al as Hyperdip>50 markers.

***Decision:*** Genes also in the top 100 chi-square probe-sets selected for *Hyperdip>50* in decision tree format that was reported by the data author (Ross, Zhou et al. 2003).

***Parallel:*** Genes also in the top 100 chi-square probe-sets selected for *Hyperdip>50* in parallel format that was reported by the data author (Ross, Zhou et al. 2003).

| **Probe-sets** | **Symbol** | **Description** | **UniGene** | **GEMs. dys** | **HDAC6** | **BAZ2A** | **Decision** | **Parallel** |
| --- | --- | --- | --- | --- | --- | --- | --- | --- |
| #overlapping |  |  |  |  | 24 | 28 | 17 | 25 |
| Fisher’s test ratio |  |  |  |  | 124 | 196 | 58 | 139 |
| Fisher’s test p |  |  |  |  | <2x10^-16^ | <2x10^-16^ | <2x10^-16^ | <2x10^-16^ |
| 212420_at | ELF1 | E74-like factor 1 (ets domain transcription factor) | Hs.135646 | down |  | Y |  |  |
| 213659_at | ZNF75 | zinc finger protein 75 (D8C6) | Hs.533540 Hs.667773 | up |  | Y |  |  |
| 205673_s_at | ASB9 | ankyrin repeat and SOCS box-containing 9 | Hs.19404 | up |  | Y |  |  |
| 204643_s_at | ENOX2 | Ecto-NOX disulfide-thiol exchanger 2 | Hs.171458 | up |  | Y |  |  |
| 214051_at | MGC39900 | hypothetical protein MGC39900 | Hs.496530 Hs.675540 | up |  | Y | Y |  |
| 203680_at | PRKAR2B | protein kinase, cAMP-dependent, regulatory, type II, beta | Hs.433068 | up |  | Y | Y |  |
| 219297_at | WDR44 | WD repeat domain 44 | Hs.98510 | up |  | Y |  | Y |
| 200642_at | SOD1 | superoxide dismutase 1, soluble (amyotrophic lateral sclerosis 1 (adult)) | Hs.443914 | up |  | Y | Y | Y |
| 212465_at | SETD3 | SET domain containing 3 | Hs.510407 | up |  | Y |  |  |
| 218694_at | ARMCX1 | armadillo repeat containing, X--linked 1 | Hs.9728 | up |  | Y | Y | Y |
| 216862_s_at | MTCP1 | mature T-cell proliferation 1 | Hs.6917 | up |  | Y |  |  |
| 221689_s_at | PIGP | phosphatidylinositol glycan anchor biosynthesis, class P | Hs.656565 | up |  | Y |  | Y |
| 209679_s_at | LOC57228 | small trans-membrane and glycosylated protein | Hs.652389 | up |  | Y |  | Y |
| 218021_at | DHRS4 | dehydrogenase/reductase (SDR family) member 4 | Hs.528385 | up |  | Y |  | Y |
| 219767_s_at | CRYZL1 | crystallin, zeta (quinone reductase)-like 1 | Hs.352671 | up |  | Y |  |  |
| 204045_at | TCEAL1 | transcription elongation factor A (SII)-like 1 | Hs.605138 | up |  | Y |  | Y |
| 202371_at | TCEAL4 | transcription elongation factor A (SII)-like 4 | Hs.194329 | up |  | Y |  | Y |
| 219335_at | ARMCX5 | armadillo repeat containing, X-linked 5 | Hs.522729 | up | Y | Y |  |  |
| 201899_s_at | UBE2A | Ubiquitin-conjugating enzyme E2A (RAD6 homolog) | Hs.379466 | up | Y | Y | Y | Y |
| 201443_s_at | ATP6AP2* | ATPase, H+ transporting, lysosomal accessory protein 2 | Hs.495960 | up | Y | Y | Y | Y |
| 213000_at | MORC3 | MORC family CW-type zinc finger 3 | Hs.421150 | up | Y | Y |  | Y |
| 201132_at | HNRPH2 | heterogeneous nuclear ribonucleoprotein H2 (H') | Hs.632828 | up | Y | Y | Y | Y |
| 213289_at | PA2G4 | Proliferation-associated 2G4, 38kDa | Hs.524498 Hs.573018 | up | Y | Y |  | Y |
| 203909_at | SLC9A6 | solute carrier family 9 (sodium/hydrogen exchanger), member 6 | Hs.62185 | up | Y | Y | Y |  |
| 205324_s_at | FTSJ1* | FtsJ homolog 1 (E. coli) | Hs.23170 | up | Y | Y | Y | Y |
| 218499_at | RP6-213H19.1 | serine/threonine protein kinase MST4 | Hs.444247 | up | Y | Y | Y | Y |
| 219485_s_at | PSMD10 | proteasome (prosome, macropain) 26S subunit, non ATPase, 10 | Hs.522752 | up | Y | Y | Y | Y |
| 218757_s_at | UPF3B | UPF3 regulator of nonsense transcripts homolog B (yeast) | Hs.103832 | up | Y | Y | Y | Y |
| 215117_at | RAG2 | recombination activating gene 2 | Hs.159376 | down | Y |  | Y |  |
| 203196_at | ABCC4 | ATP-binding cassette, sub-family C (CFTR/MRP), member 4 | Hs.508423 | down | Y |  |  |  |
| 210517_s_at | AKAP12 | A kinase (PRKA) anchor protein (gravin) 12 | Hs.371240 | down | Y |  |  |  |
| 205583_s_at | CXorf45 | chromosome X open reading frame 45 | Hs.110853 Hs.443061 | up | Y |  |  |  |
| 203981_s_at | ABCD4 | ATP-binding cassette, sub-family D (ALD), member 4 | Hs.94395 | up | Y |  |  |  |
| 218573_at | MAGEH1* | melanoma antigen family H, 1 | Hs.279819 | up | Y |  | Y |  |
| 208117_s_at | LAS1L | LAS1-like (S. cerevisiae) | Hs.522675 | up | Y |  |  | Y |
| 212846_at | RRP1B | ribosomal RNA processing 1 homolog B (S. cerevisiae) | Hs.654727 | up |  |  |  | Y |
| 203776_at | GPKOW | G patch domain and KOW motifs | Hs.503666 | up | Y |  |  | Y |
| 209620_s_at | ABCB7 | ATP-binding cassette, sub-family B (MDR/TAP), member 7 | Hs.370480 | up | Y |  |  | Y |
| 215884_s_at | UBQLN2 | ubiquilin 2 | Hs.179309 | up | Y |  |  | Y |
| 201100_s_at | USP9X | ubiquitin specific peptidase 9, X-linked | Hs.77578 | up | Y |  | Y | Y |
| 209565_at | RNF113A | ring finger protein 113A | Hs.458365 | up | Y |  | Y | Y |
| 216071_x_at | MED12 | mediator complex subunit 12 | Hs.409226 | up | Y |  | Y | Y |

*: Genes that are known as x-linked mental retardation markers (Ross, Zhou et al. 2003).
