# Supplementary material for: Mechanism-anchored profiling derived from epigenetic networks predicts outcome in acute lymphoblastic leukemia
Source: BMC Bioinformatics. 2009 Sep 17;10(Suppl 9):S6. doi: 10.1186/1471-2105-10-S9-S6 (PMC2745693; doi:10.1186/1471-2105-10-S9-S6)
Supplement: Additional file 12 — Supplementary Table 7 – Comparison GEMs in PGnet with genes identified by ARACNE. [file 1471-2105-10-S9-S6-S12.doc]

**Supplementary Table 7**

**Comparison GEMs in PGnet with genes identified by ARACNE**

| **Phenotype** | **Co-expressed genes** | **Reversed co-expressed genes** |
| --- | --- | --- |
| BCR-ABL |  | DNMT3B* HDAC4* CBX1* |
| E2A-PBX1 | PHLDA2 | CBX6 BAZ2B MYST4* MYST2 MCEP2* SMARCA2 HDAC7A |
| Hyperdip>50 | HDAC6 | BAZ2A SMARCA4* SMARCC2 |
| MLL | DNMT3B HDAC9 CBX4 SMARCA2 SMYD3* |  |
| Normal | IGF2 | SUV39H1 CBX5* MYST4* |
| Pseudodip | SMARCD2 |  |
| T-ALL | BAZ2B DNMT3B* HDAC4* MARCAL1 SMYD3* CBX1* | HDAC5 SMARCD2 SMARCA2 PRDM2 |
| TEL-AML1 | MECP2* PRDM2 | MBD2* HDAC9 |
| 2nd_AML |  | CBX6 |
| CCR | CBX6 | HDAC9 |
| Relapse |  | SUV39H1 CBX5* |

*GEMs in direct-transcriptional regulation network according to ARACNE.
