# Supplementary material for: Mechanism-anchored profiling derived from epigenetic networks predicts outcome in acute lymphoblastic leukemia
Source: BMC Bioinformatics. 2009 Sep 17;10(Suppl 9):S6. doi: 10.1186/1471-2105-10-S9-S6 (PMC2745693; doi:10.1186/1471-2105-10-S9-S6)
Supplement: Additional file 13 — Supplementary Table 8 – Comparison of the results from ARACNE and PGnet for leukemia phenotypes. [file 1471-2105-10-S9-S6-S13.doc]

**Supplementary Table 8**

**Comparison of the results from ARACNE and PGnet for leukemia phenotypes**

***LP:*** Leukemia Phenotype

***ESG:*** Epigenetic Seed Gene

***ARACNE:*** The number of predicted genes by ARACNE that associated with corresponding ESG in samples in the corresponding LP.

***PGnet:*** The number of predicted genes by PGnet that associated with corresponding ESG in samples in the corresponding LP.

***Intersection:*** The number of genes that predicted by both ARACNE and PGnet

***Lowest rank*** The lowest rank of multiple information (MI) estimated by ARACNE for “intersection” genes.

***Highest rank:*** The highest rank of multiple information (MI) estimated by ARACNE for “intersection” genes.

| **LP** | **ESG** | **ARACNE** | **PGnet** | **Intersection** | **Lowest rank** | **Highest rank** |
| --- | --- | --- | --- | --- | --- | --- |
| Relapse | CBX5 | 857 | 17 | 7 | 511 | 807 |
|  | SUV39H1 | 1241 | 22 | 6 | 294 | 983 |
|  | DNMT3A | 893 | 6 | 4 | 38 | 681 |
|  | HDAC9 | 1158 | 5 | 3 | 13 | 199 |
| Normal | CBX5 | 795 | 16 | 4 | 5 | 78 |
|  | SUV39H1 | 740 | 23 | 3 | 16 | 729 |
| CCR | HDAC9 | 1284 | 13 | 10 | 14 | 768 |
| MLL | HDAC9 | 684 | 51 | 3 | 364 | 662 |
|  | SMARCA2 | 684 | 31 | 4 | 79 | 639 |
| TEL-AML1 | HDAC9 | >1172 | 47 | 5 | 109 | 1151 |
|  | DNMT3A | 694 | 25 | 3 | 80 | 530 |
|  | MBD2 | >1172 | 20 | 2 | 103 | 475 |
| Hyperdip>50 | HDAC6 | >1128 | 25 | >14 | 55 | 1015 |
|  | SMARCA4 | >1128 | 17 | >5 | 248 | 1049 |
|  | CBX7 | 1024 | 7 | 2 | 50 | 250 |
|  | BAZ2A | >1128 | 27 | >6 | 267 | 974 |
| Pseudodip | BAZ2A | 1321 | 9 | 3 | 42 | 826 |
| BCR-ABL | DNMT3B | 963 | 12 | 3 | 570 | 866 |
|  | HDAC4 | 923 | 31 | 3 | 51 | 501 |
|  | CBX1 | 1047 | 25 | 1 | 770 |  |
| T-ALL | DNMT3B | 904 | 40 | 2 | 343 | 826 |
|  | HDAC4 | >1229 | 56 | >14 | 25 | 1037 |
|  | SMYD3 | 119 | 17 | 0 |  |  |
|  | HDAC5 | >1229 | 25 | >4 | 113 | 1037 |
|  | PRDM2 | 974 | 30 | 4 | 72 | 883 |
| E2A-PBX1 | CBX6 | 659 | 21 | 2 | 335 | 493 |
|  | MECP2 | 937 | 33 | 2 | 108 | 663 |
|  | PHLDA2 | >1021 | 34 | >7 | 73 | 1002 |
|  | BAZ2B | >1021 | 45 | 11 | 22 | 962 |
|  | HDAC7 | >1021 | 38 | >4 | 635 | 1011 |
|  | SMARCA2 | 718 | 30 | 5 | 483 | 716 |
|  | MYST2 | 474 | 26 | 0 |  |  |
|  | MYST4 | >1021 | 19 | >2 | 727 | 909 |
