# Supplementary material for: Mechanism-anchored profiling derived from epigenetic networks predicts outcome in acute lymphoblastic leukemia
Source: BMC Bioinformatics. 2009 Sep 17;10(Suppl 9):S6. doi: 10.1186/1471-2105-10-S9-S6 (PMC2745693; doi:10.1186/1471-2105-10-S9-S6)
Supplement: Additional file 14 — Supplementary Figure 1 – PGnet: significant "phenotypes in ALL – GEMs – epigenetic seed genes" [file 1471-2105-10-S9-S6-S14.doc]

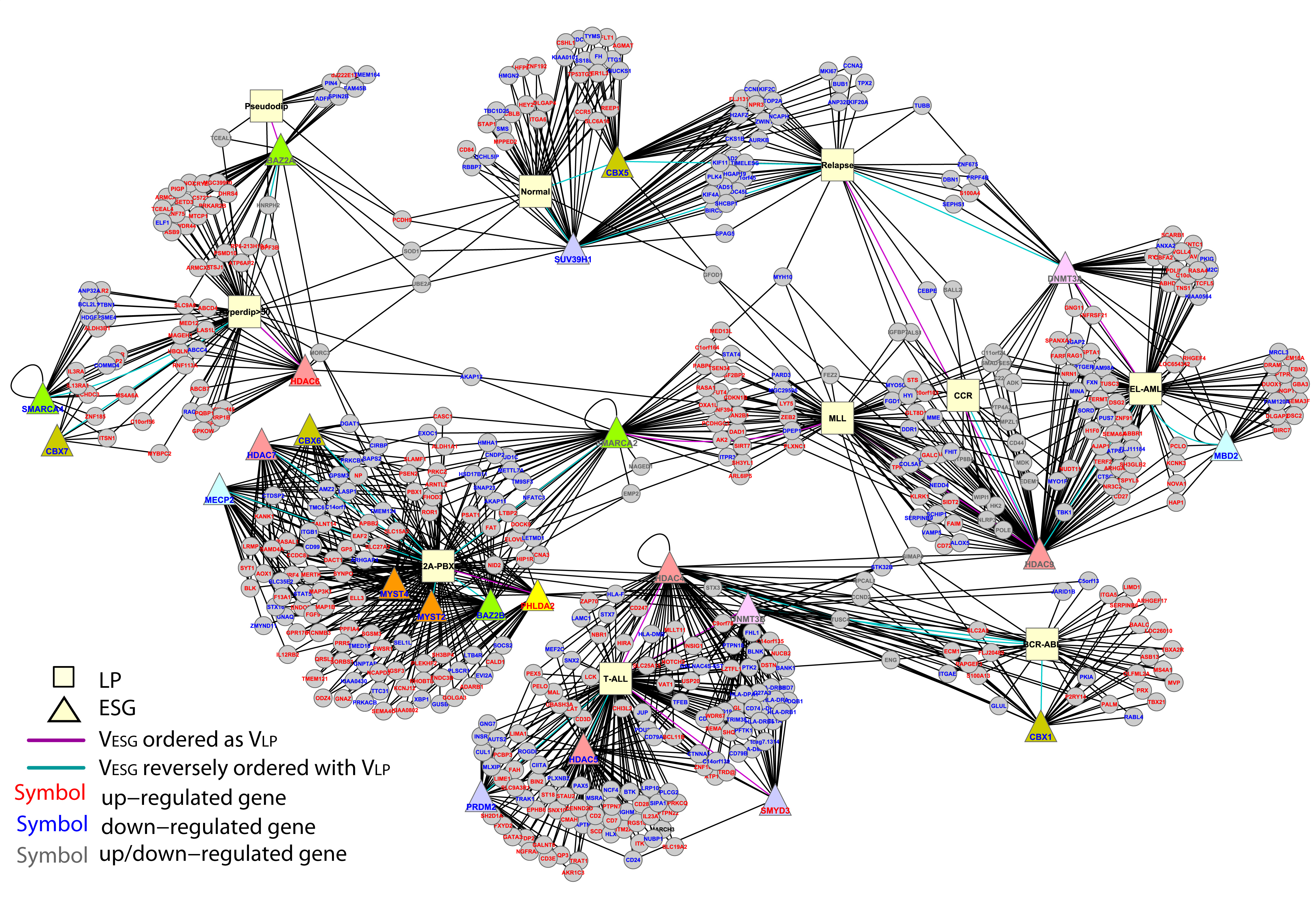


**Supplementary Figure 1.** **PGnet: significant “phenotype in ALL – GEMs - Epigenetic seed genes”.** The predicted “GEMs” (grey circles) are co-regulated with their corresponding epigenetic seed gene (ESGs, triangles) and co-differentially expressed in linked leukemia phenotypes (LP, yellow boxes). Red genes are up-regulated in the associated LP, while blue ones are down-regulated, and grey ones are related to more than one phenotypes with alternate up-down regulations (details of the full network in **Suppl. Table 2**). *Orderedlist* parameter T=200 (**Methods**).
